# Seasonality of suicide: a multi-country multi-community observational study

**DOI:** 10.1017/S2045796020000748

**Published:** 2020-08-24

**Authors:** J. Yu, D. Yang, Y. Kim, M. Hashizume, A. Gasparrini, B. Armstrong, Y. Honda, A. Tobias, F. Sera, A. M. Vicedo-Cabrera, H. Kim, C. Íñiguez, E. Lavigne, M. S. Ragettli, N. Scovronick, F. Acquaotta, B. Chen, Y. L. Guo, M. de Sousa Zanotti Stagliori Coelho, P. Saldiva, A. Zanobetti, J. Schwartz, M. L. Bell, M. Diaz, C. De la Cruz Valencia, I. Holobâcă, S. Fratianni, Y. Chung

**Affiliations:** 1 Department of Mathematical Sciences, Korea Advanced Institute of Science and Technology, Daejeon, South Korea; 2 Department of Global Environmental Health, Graduate School of Medicine, University of Tokyo, Tokyo, Japan; 3 Department of Global Health Policy, Graduate School of Medicine, University of Tokyo, Tokyo, Japan; 4 Department of Public Health, Environments and Society, London School of Hygiene & Tropical Medicine, London, UK; 5 Centre for Statistical Methodology, London School of Hygiene & Tropical Medicine, London, UK; 6 Centre on Climate Change and Planetary Health, London School of Hygiene & Tropical Medicine, London, UK; 7 Faculty of Health and Sport Sciences, University of Tsukuba, Tsukuba, Japan; 8 Institute of Environmental Assessment and Water Research, Spanish Council for Scientific Research, Barcelona, Spain; 9 Institute of Social and Preventive Medicine, University of Bern, Bern, Switzerland; 10 Oeschger Center for Climate Change Research, University of Bern, Bern, Switzerland; 11 Graduate School of Public Health, Seoul National University, Seoul, Republic of Korea; 12 Department of Statistics and Computational Research, Universitat de València, València, Spain; 13 CIBER Epidemiolgia y Salud Publica (CIBERESP), Madrid, Spain; 14 School of Epidemiology & Public Health, University of Ottawa, Ottawa, Canada; 15 Air Health Science Division, Health Canada, Ottawa, Canada; 16 Department of Epidemiology and Public Health, Environmental Exposures and Health Unit, Swiss Tropical and Public Health Institute, Basel, Switzerland; 17 University of Basel, Basel, Switzerland; 18 Gangarosa Department of Environmental Health, Rollins School of Public Health, Emory University, Atlanta, USA; 19 Department of Earth Sciences, University of Torino, Turin, Italy; 20 National Institute of Environmental Health Sciences, National Health Research Institutes, Zhunan, Taiwan; 21 Department of Environmental and Occupational Medicine, National Taiwan University College of Medicine and National Taiwan University Hospital, Taipei, Taiwan; 22 Institute of Advanced Studies, University of São Paulo, São Paulo, Brazil; 23 Department of Environmental Health, Harvard T.H. Chan School of Public Health, Boston, Massachusetts, USA; 24 School of Forestry and Environmental Studies, Yale University, New Haven, Connecticut, USA; 25 Department of Environmental Health, National Institute of Public Health, Cuernavaca, Morelos, Mexico; 26 Faculty of Geography, Babes-Bolay University, Cluj-Napoca, Romania

**Keywords:** Climate, heterogeneity, seasonality, socioeconomic, suicide

## Abstract

**Aims:**

We aimed to investigate the heterogeneity of seasonal suicide patterns among multiple geographically, demographically and socioeconomically diverse populations.

**Methods:**

Weekly time-series data of suicide counts for 354 communities in 12 countries during 1986–2016 were analysed. Two-stage analysis was performed. In the first stage, a generalised linear model, including cyclic splines, was used to estimate seasonal patterns of suicide for each community. In the second stage, the community-specific seasonal patterns were combined for each country using meta-regression. In addition, the community-specific seasonal patterns were regressed onto community-level socioeconomic, demographic and environmental indicators using meta-regression.

**Results:**

We observed seasonal patterns in suicide, with the counts peaking in spring and declining to a trough in winter in most of the countries. However, the shape of seasonal patterns varied among countries from bimodal to unimodal seasonality. The amplitude of seasonal patterns (i.e. the peak/trough relative risk) also varied from 1.47 (95% confidence interval [CI]: 1.33–1.62) to 1.05 (95% CI: 1.01–1.1) among 12 countries. The subgroup difference in the seasonal pattern also varied over countries. In some countries, larger amplitude was shown for females and for the elderly population (≥65 years of age) than for males and for younger people, respectively. The subperiod difference also varied; some countries showed increasing seasonality while others showed a decrease or little change. Finally, the amplitude was larger for communities with colder climates, higher proportions of elderly people and lower unemployment rates (*p*-values < 0.05).

**Conclusions:**

Despite the common features of a spring peak and a winter trough, seasonal suicide patterns were largely heterogeneous in shape, amplitude, subgroup differences and temporal changes among different populations, as influenced by climate, demographic and socioeconomic conditions. Our findings may help elucidate the underlying mechanisms of seasonal suicide patterns and aid in improving the design of population-specific suicide prevention programmes based on these patterns.

## Introduction

Abundant research dating back to the 19th century demonstrates seasonal suicide patterns, with an increase in spring and a decrease in winter (Durkheim, 1897; Maes *et al*., 1993; Chew and McCleary, 1995; Ajdacic-Gross *et al*., 2010; Christodoulou *et al*., 2012; Woo *et al*., 2012; Roehner, 2015; Dixon and Kalkstein, 2018; Yang *et al*., 2019). While the pattern of seasonal suicides has been confirmed in many countries, the mechanisms remain unclear. The most plausible underlying mechanisms for seasonal suicide patterns include seasonal fluctuations of social activities (Souêtre *et al*., 1990; Maes *et al*., 1993; Chew and McCleary, 1995; Ajdacic-Gross *et al*., 2010; Christodoulou *et al*., 2012; Roehner, 2015) and bioclimatic factors, such as temperature or amount of sunshine (Souêtre *et al*., 1987, 1990; Linkowski *et al*., 1992; Maes *et al*., 1994; Deisenhammer *et al*., 2003; Lambert *et al*., 2003; Papadopoulos *et al*., 2005; Ruuhela *et al*., 2009; Muller *et al*., 2011; Vyssoki *et al*., 2012; Moore *et al*., 2018).

Comparison of seasonal suicide patterns across geographically, demographically and socioeconomically heterogeneous populations should help elucidate the underlying factors influencing these patterns and better explain the mechanisms of the phenomenon. However, almost all previous studies on seasonal suicides investigated a study population that was relatively homogeneous (e.g. a single city, a single country or a few communities within a country) (Woo *et al*., 2012). Moreover, these studies differed in terms of experimental design and statistical methodology, making it difficult to directly compare results or quantify heterogeneity among different populations (Woo *et al*., 2012).

To evaluate heterogeneity and underlying determinants systematically, a large-scale, multi-country, multi-community study is necessary as it allows for analysing the time-series data of the number of suicides from multiple populations in a unified modelling framework. One previous study (Chew and McCleary, 1995) evaluated between-country variability in spring suicide peaks and examined how this variability is associated with bioclimatic and socio-demographic factors, but was limited to the use of country-specific, monthly data collected between the 1960s and 1980s, and the analysis of simple, descriptive indices of seasonality. No other previous studies conducted a large-scale multi-population analysis.

Here, we investigated seasonal suicide patterns across 354 communities from 12 countries by analysing weekly time-series data of suicide numbers between 1986 and 2016 using a unified statistical modelling framework. We aimed to (1) compare the shape and amplitude of seasonal suicide patterns across countries and communities; (2) examine how seasonality differs by sex, age group and subperiod and (3) identify community-specific characteristics modifying seasonal suicide patterns. To our knowledge, this is the first large-scale, multi-country, multi-community study to comprehensively examine heterogeneity of seasonality in suicide and its underlying determinants among highly diverse populations.

## Methods

### Data collection

**Fig. 1.**
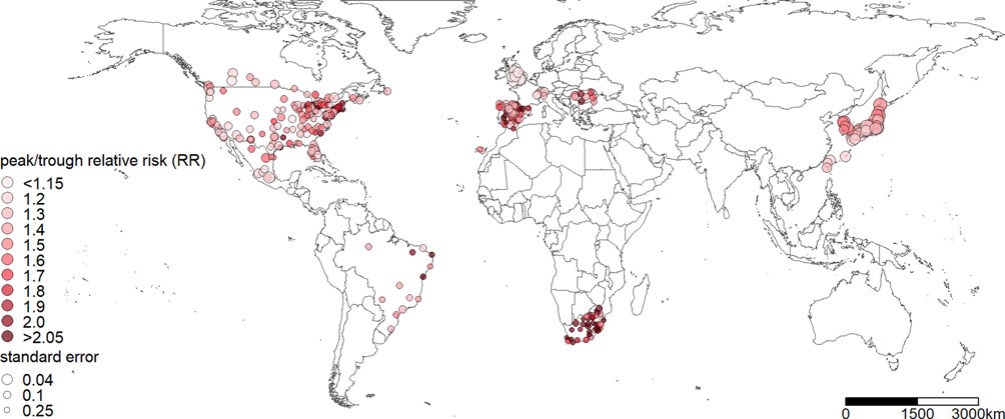
Spatial map of the location of 354 communities in 12 countries with the peak/trough relative risk (RR) of suicide estimated from the first-stage modeling. The size of the points corresponds to the precision of the RR estimate (i.e., the inverse of the standard error of the community-specific RR).

Daily time-series data of suicide numbers were collected across 354 communities in 12 countries. Geographical locations of all communities are displayed in Fig. 1. The collection period varied by country, ranging from 6 (the USA) to 30 years (Canada) (Table 1). Suicide was defined as intentional self-poisoning and self-harm based on the International Statistical Classification of Diseases and Related Health Problems (ICD) (E950.0–E958.9 for the ICD-8 and -9 and X60–X84 for the ICD-10). Data collection details are available in the Supplement. We derived the weekly sum of suicide numbers to increase statistical power, given that numerous zero values were observed in the daily data. A week was defined as an interval of seven days, starting from 1st January of each year. Thus, each year was comprised of 53 weeks, with the final week including only one or two days. For the final week, weekly suicide numbers were adjusted using the number of suicides from the previous week (i.e. 52nd week of the same year) and the following week (i.e. 1st week of the next year). Details for the adjustment are available in the Supplement. Summary statistics of the total number of suicides for each community are available in eTable 1 and histograms of the number of weekly suicides for each community are available in eFig. 1 in the Supplement.

**Table 1.** Summary statistics of the number of suicides for each of the 12 countries

Country	Number of communities	Study period (years)	Total number of suicides	Average number of weekly suicides^a^	Total number of male suicides (%)	Total number of non-elderly suicides (%)
Brazil	13	1997–2005 (9 y)	8801	18.67	6787 (77.12)	8178 (92.92)
Canada	26	1986–2015 (30 y)^b^	51 815	33.06	38 620 (74.53)	44 993 (86.83)
Japan	47	1986–2012 (27 y)	706 840	500.41	485 956 (68.75)	518 932 (73.42)
Mexico	10	1998–2014 (17 y)	26 329	29.71	21 309 (80.93)	24 614 (93.49)
Romania	8	1999–2016 (18 y)	5380	5.70	4281 (79.57)	4263 (79.24)
South Africa	39	2000–2013 (14 y)	5128	7.09	3993 (77.87)	4814 (93.88)
South Korea	6	1992–2013 (22 y)	83 825	72.83	56 390 (67.27)	66 266 (79.05)
Spain	50	1990–2013 (24 y)	21 998	17.56	15 650 (71.14)	15 425 (70.12)
Switzerland	8	1995–2013 (19 y)	16 022	16.16	11 072 (69.10)	10 672 (66.61)
Taiwan	3	1994–2007 (14 y)	17 883	24.44	12 054 (67.40)	14 072 (78.69)
The United Kingdom (UK)	10	1990–2011 (22 y)	78 115	68.01	60 326 (77.22)	64 890 (83.07)
The United States (USA)	134	2001–2006 (6 y)	84 684	270.20	66 110 (78.07)	70 581 (83.35)

a Average number of weekly suicides = (Total number of suicides)/(study years × 53 weeks).

b The study period for the city of Montreal is 1992–2015 (24 years).

Data for community-level indicators were collected from a recent multi-country study (Sera *et al*., 2019). Demographic and socioeconomic indicators were extracted from the Organization for Economic Co-operation and Development (OECD) Regional and Metropolitan Database and the World Cities Database. Demographic variables included total population, population density (population/km^2^), proportion of people aged ≥65 years and life expectancy. Socioeconomic variables included gross domestic product (GDP), unemployment rate and educational level. Data for air pollution and climate indicators were obtained from a previous study (Sera *et al*., 2019); global estimates of the annual average concentrations of fine (diameter <2.5 μm) particulate matter (PM_2.5_) and ground-level measurements of nitrogen dioxide (NO_2_) concentrations were available, and the overall average daily mean temperature over the study period was derived. More information for the indicators is available in eTable 2 in the Supplement.

### Statistical analyses

#### Error/trend/season (ETS) decomposition

To evaluate the portion of the weekly variation in suicide numbers that was explained by the seasonal components, weekly time-series data were decomposed into trend, seasonal and remainder (i.e. error) components by the seasonal-trend decomposition using the Loess (STL) method (Cleveland *et al*., 1990). We used R statistical software (version 3.6.1; R Development Core Team) with the stl() function in the forecast package.

#### Estimation of the seasonality of suicide

A two-stage analysis was conducted to estimate community-specific and country-specific seasonality of suicide. Mathematical details are available in the Supplement. In the first stage, we estimated seasonality for each community using a generalised linear model with a quasi-Poisson distribution. We controlled for the effect of year by including indicators for each year. We modelled nonlinear and cyclic seasonal patterns of suicide through a cyclic B-spline basis function with four degrees of freedom (df) applied to the week variable (using values from 1 to 53). The choice of df = 4 was guided by model evaluation based on the likelihood ratio test, the quasi-Akaike information criterion (QAIC), and the interpretability of the results. Particularly, we considered the largest communities of each country for model evaluation, as the statistical uncertainty for the estimated seasonal patterns was the smallest. Details for model evaluation are presented in the Supplement. From the chosen model, community-specific parameters for seasonality were estimated to generate curves of the relative risk (RR) of suicide over all 53 weeks *v.* the week with the lowest number of suicides. Additionally, the peak/trough RR was derived as a single number summarising the community-specific amplitude of seasonality. In the second stage, community-specific parameters for seasonality were combined using multivariate meta-regression with country indicators included as meta-predictors. From the fitted meta-regression, country-specific parameters for seasonality were extracted to generate country-specific RR curves and peak/trough RR. We used R statistical software (version 3.6.1; R Development Core Team) with the mixmeta() function in the mixmeta package for the second-stage modelling.

#### Estimation of the seasonality of suicide by sex, age group and subperiod

We estimated the seasonality of suicide by sex, age group (<65 or ≥65 years) and subperiod (before or after the year 2000) through the two-stage analysis. For the subperiod analysis, Mexico, Romania, South Africa and the USA were excluded because their data were mostly available only after 2000. To estimate subgroup (or subperiod) differences, we estimated community-specific seasonality for each subgroup in the first-stage analysis, and combined the community-specific parameters from both subgroups using multivariate meta-regression including country indicators, a binary indicator for subgroup, and their interactions as meta-predictors in the second-stage modelling. From the meta-regression, we extracted subgroup-specific seasonality parameters and generated the RR curves for each subgroup and each country. Using multivariate Wald test, we assessed whether the RR curves significantly differed between subgroups for each country.

#### Influence of community-level indicators on the seasonality of suicide

Each community-level indicator was standardised by subtracting country-specific means and scaling by country-specific standard deviations. The impact of all indicators on the seasonality of suicide was investigated by simultaneously controlling for each other indicator through the two-stage analysis. Community-specific seasonality parameters were extracted from the first-stage model and pooled by meta-regression with country indicators and all of the standardised community-level indicators included as meta-predictors. The modelling included 269 communities of six countries (Canada, Japan, South Korea, Spain, the UK and the USA) because of the data availability. A stepwise variable selection was conducted to identify the optimal subset of indicators starting with a minimal model that only included country indicators. Adding an indicator was determined based on the largest reduction in the AIC, and the importance of an added indicator was presented as the AIC, *I*^2^ statistic and *p*-value of the Wald test. We used the step() function in the mixmeta package for variable selection. The effect of the selected indicators on seasonal patterns was illustrated by the RR curve, estimated with each indicator fixed at the 10th and 90th percentiles for each country, controlling for the other indicators set as each country's mean.

#### Sensitivity analysis

We evaluated sensitivity of the results based on the choice of the degrees of freedom specified for the cyclic spline in the first-stage modelling. We conducted the two-stage modelling with df = 2, 3, 5, 6 and 7 to compare with the main results derived with the chosen df = 4.

## Results

**Fig. 2.**
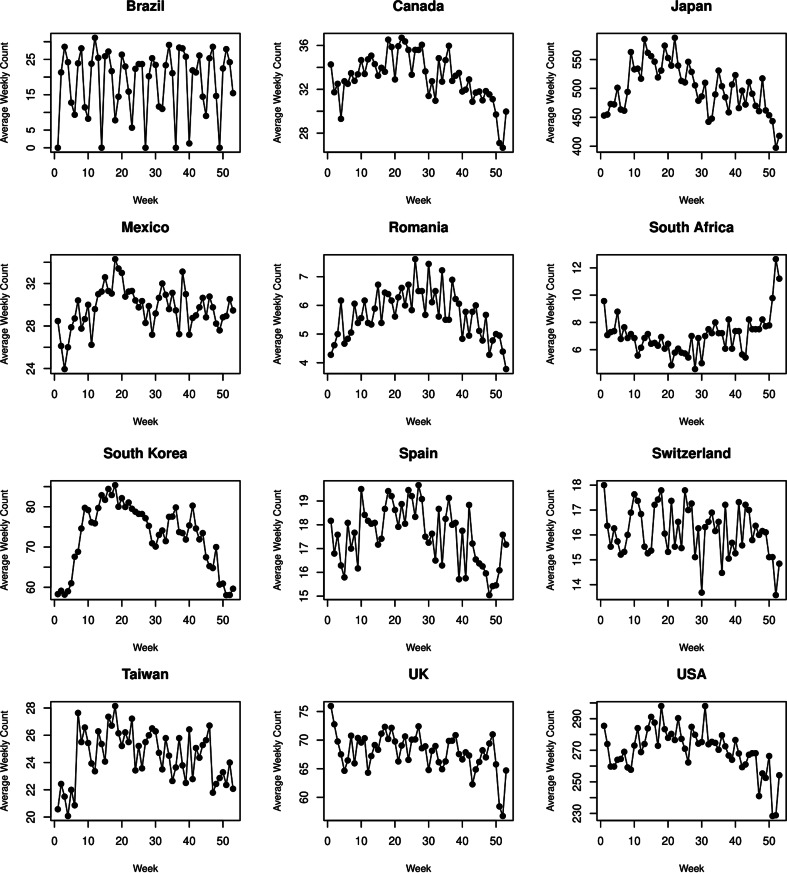
Average weekly number of suicides for each country for the entire study period.

Table 1 presents summary statistics of suicide numbers for each country. The total number of suicides considered was 1 106 820. The proportions of male and non-elderly suicides exceeded 67 and 66%, respectively, with variability among countries. The average weekly suicide counts were presented for each country in Fig. 2. They indicate that there exist strong seasonal patterns of suicide in most of the countries, while the patterns were heterogeneous among the countries. The ETS decomposition revealed that the contribution of seasonal components to the weekly suicide counts varied across countries (eFig. 2 in the Supplement). In Brazil, Japan, South Africa, South Korea, Taiwan and the USA, seasonal and error components seemed to explain the weekly variation with comparable portions, once the trend was removed. In Canada, Mexico, Romania, Switzerland, Spain and the UK, the portion of the weekly variation explained by seasonal components was relatively small.

**Fig. 3.**
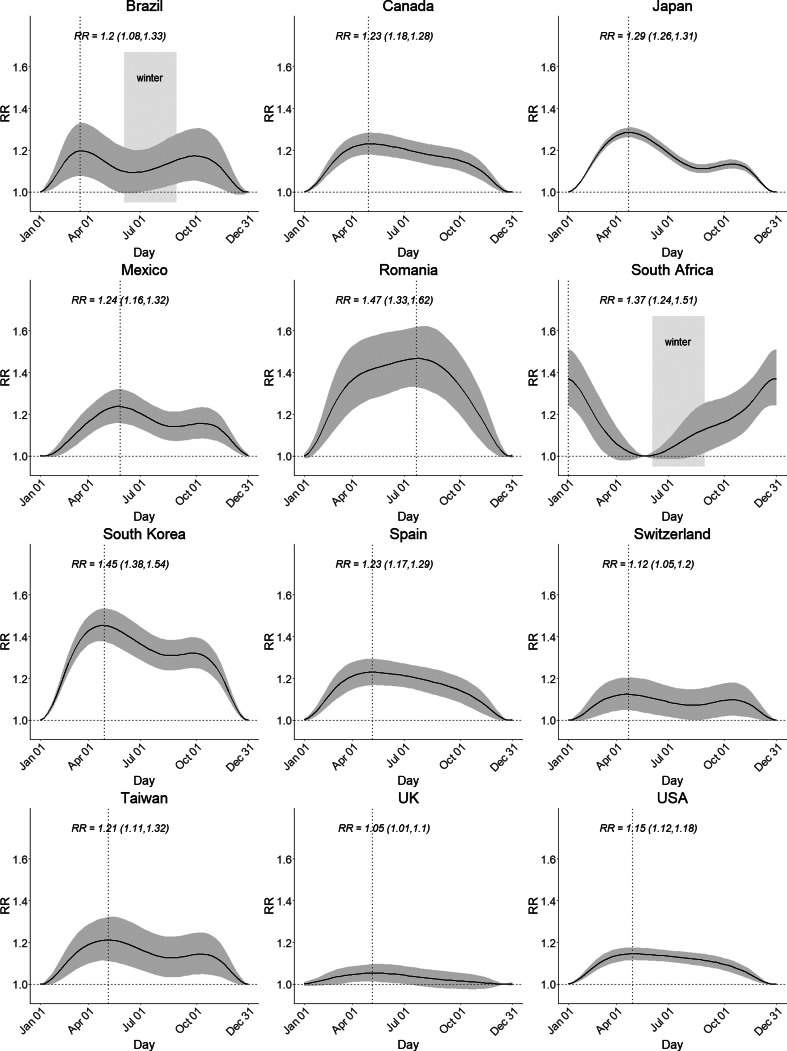
Country-specific seasonality of suicide. The y-axis represents the relative risk (RR) of suicide for all other weeks versus the week in which the estimated number of suicides is lowest. The shaded area indicates the 95% confidence intervals. The dotted lines indicate the week in which the estimated number of suicides was highest. The peak/trough RR is presented with 95% confidence intervals. The winter seasons are marked for the countries in the Southern Hemisphere (Brazil and South Africa).

Figure 3 shows country-specific seasonal patterns of suicide (see eFig. 3 in the Supplement for community-specific seasonal patterns). Northern Hemisphere countries exhibited spring peaks (April–May) and winter troughs (December–January), except for Romania peaking in summer (July). For Southern Hemisphere countries, Brazil exhibited two peaks in the spring and autumn (October and April) and a trough in the summer (January), while South Africa exhibited a peak in the summer (January) and a trough in the late autumn (June). In countries such as Brazil, Japan, Mexico, South Korea, Switzerland and Taiwan, seasonality was bimodal, with the second peak observed in the autumn. In other countries including Canada, Romania, South Africa, Spain, UK and the USA, unimodal seasonality was exhibited. The peak/trough RR varied between countries; Romania showed the highest value of 1.47 (95% CI: 1.33–1.62) followed by South Korea with 1.45 (95% CI: 1.38–1.54) and South Africa with 1.37 (95% CI: 1.24–1.51), respectively. The RRs were between 1.2 and 1.3 for Brazil, Canada, Japan, Mexico, South Africa, Spain and Taiwan, while below 1.2 for Switzerland, the UK and the USA. The country-specific peak and trough weeks and the peak/trough RR are reported in eTable 3 in the Supplement.

**Fig. 4.**
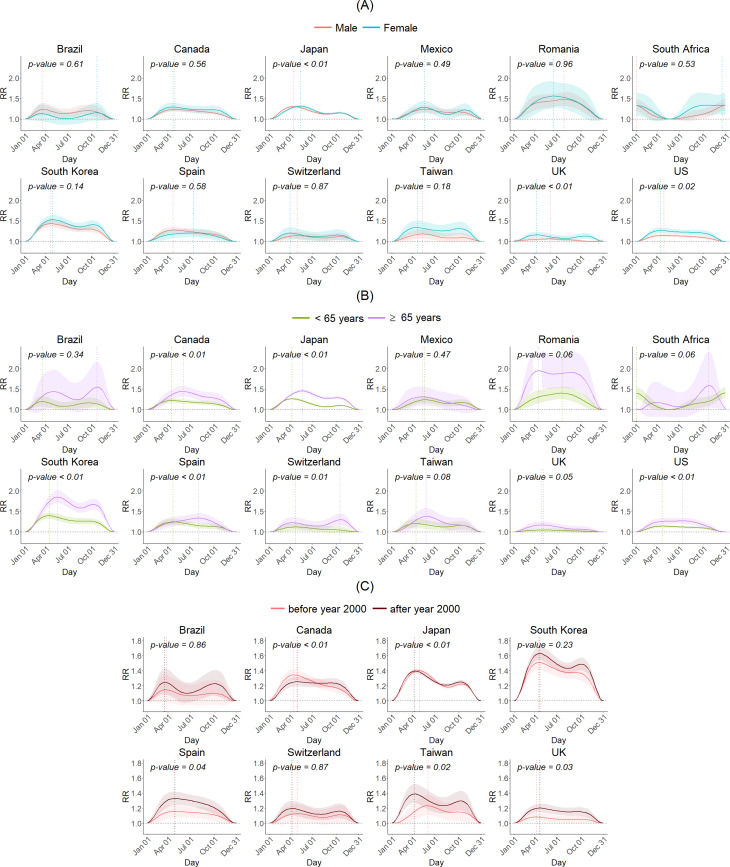
(A) Sex-specific, (B) age group-specific, and (C) subperiod-specific seasonality of suicide for each country. The y-axis represents the relative risk (RR) of suicide for all other weeks versus the week in which the estimated number of suicides is lowest. The shaded areas indicate the 95% confidence intervals. The dotted lines indicate the week of the year in which the estimated number of suicides was highest. The p-value was calculated from the multivariate Wald test, comparing the RR curves between two subgroups or subperiods.

Figure 4 presents the sex-, age group- and subperiod-specific seasonality of suicide for each country. Seasonal patterns differed significantly by sex in Japan, the UK and the USA (*p* < 0.05) (Fig. 4*a*). In Japan, the number of suicides peaked for males 3 weeks earlier than for females. In the UK and the USA, females displayed a seasonal pattern with a larger amplitude than males. Also, the seasonal pattern differed by age groups in several countries (Fig. 4*b*); the amplitude was larger for older than for younger people in Canada, Japan, South Korea, Spain, Switzerland and the USA (*p* < 0.05). Additionally, the seasonality differed between the two subperiods in some countries (Fig. 4*c*); the spring peak was lower after 2000 in Canada (*p* < 0.05), the peak was pushed forward after 2000 in Japan (*p* < 0.05), while the spring peak increased after 2000 in Spain, Taiwan and UK (*p* < 0.05).

Table 2 summarises the impact of the community-level indicators on the seasonality of suicide. Applying a stepwise variable selection in meta-regression, the final model included country indicator, average temperature, unemployment rate and proportion of people aged ≥65 years. The effects of each indicator on the seasonal pattern are illustrated in eFig. 4 in the Supplement. The seasonal suicide pattern was larger for communities with lower temperatures, lower unemployment rates and higher proportions of people aged ≥65, adjusting for each other indicator.

**Table 2.** Model selection for community-specific indicators using 269 communities of six countries

Model	AIC	*I*^2^ (%)	Wald test (*p*-value)	
Country	−296.93	32.22	Country	<0.0001
Country and average temperature	−320.43	26.00	Country Average temperature	<0.0001 <0.0001
Country and average temperature and unemployment rate	−329.57	24.36	Country Average temperature Unemployment rate	<0.0001 <0.0001 0.0004
Country and average temperature and unemployment rate and proportion of people aged ≥65 years	−338.03	21.79	Country	<0.0001
			Average temperature Unemployment rate	<0.0001 0.0004
			Proportion of people ≥65 years	0.0008

We conducted sensitivity analysis using cyclic splines with df = 2, 3, 5, 6 and 7. The country-specific RR curves are presented in eFigs 5–9. The RR curves with df = 2 exhibited unimodal seasonal patterns in all countries, clearly underfitting the seasonality in many countries. RR curves with df = 3 were similar to RR curves with df = 4 in Fig. 3; they captured the bimodal seasonal pattern in countries including Brazil, Japan, South Korea and Taiwan, while preserving the unimodal pattern in the other countries. RR curves with df = 3 showed slightly sharper peaks and lower peak/trough RR than those with df = 4. RR curves with df = 5, 6 and 7 overfit the seasonality in several countries, with ‘wiggly’ patterns complicating interpretation, unnecessarily increasing model complexity.

## Discussion

We investigated heterogeneity in the seasonality of suicide across 354 communities in 12 countries by analysing weekly time-series data of suicide numbers from 1986 to 2016. To our knowledge, this is the first large scale, multi-country, multi-community study to examine heterogeneity and its underlying determinants, particularly among populations that are highly diverse using a long period with up-to-date data. We confirmed that the spring peak in suicide numbers was consistently observed in almost all countries, but the shape and amplitude of seasonality, subgroup difference and temporal changes varied between countries. We observed that seasonal effects on suicide were greater for females and the elderly than for males and younger people in some countries, and the amplitude tended to be larger in communities with colder climates, higher proportions of the elderly and lower unemployment rates. The temporal change of seasonality was inconclusive; the seasonal pattern increased, decreased or remained roughly constant over the subperiods depending on the country.

Our results showed that suicide numbers peaked in spring in most countries replicating those of previous studies. The most substantiated explanation for the spring peak is the seasonal cycle of bioclimatic factors such as temperature and amount of sunshine (Souêtre *et al*., 1987, 1990; Linkowski *et al*., 1992; Maes *et al*., 1993, 1994; Chew and McCleary, 1995; Deisenhammer *et al*., 2003; Lambert *et al*., 2003; Papadopoulos *et al*., 2005; Ruuhela *et al*., 2009; Ajdacic-Gross *et al*., 2010; Muller *et al*., 2011; Christodoulou *et al*., 2012; Vyssoki *et al*., 2012; Moore *et al*., 2018). Specifically, levels of serotonin, a neurotransmitter that regulates emotion, are sensitive to weather variability and light exposure, so springtime changes in expression may be associated with increased suicidal behaviour (Brewerton, 1989; Praschak-Rieder *et al*., 2008). Another well-accepted interpretation for the spring peak is socio-psychiatric mechanisms; high intensity social or occupational activities in the spring may be the underlying driver of spring spikes in suicide numbers (Ajdacic-Gross *et al*., 2010; Christodoulou *et al*., 2012). In many countries, spring is a season of renewal and is, therefore, likely to cause more social stress, which is a possible trigger for suicide. People may also experience disappointment with excessive expectations for a new start, which is a plausible psychiatric mechanism to explain the spring peak in suicides, known as the ‘broken promise effect’ (Gabennesch, 1988; Tsouvelas *et al*., 2019). Though the spring peak was a common feature, two countries were exceptions: Romania and South Africa showed summer peaks. There may exist some country-specific factors that could explain such a discrepancy, though further evidence is needed for confirmation.

Despite the common feature of the spring peak, the seasonal suicide pattern was heterogeneous in overall shape. Many countries exhibited bimodal seasonality, with the highest peak in spring and the second highest peak in autumn in northeast Asian countries (Japan, South Korea and Taiwan) and Mexico (Fig. 2), which was replicated in South Korea, Japan, Finland and Sweden (Likhvar *et al*., 2011; Holopainen *et al*., 2013; Yang *et al*., 2019). Assuming that the previously explained bioclimatic and socio-psychiatric mechanisms underlie the seasonal suicide patterns, the major peak in springtime may be influenced by both mechanisms simultaneously, while the local peak in autumn may be attributed mostly to the bioclimatic mechanisms. This explanation, however, may not apply to several countries in Europe or the North America, for which the seasonal pattern was a unimodal shape, with a plateau from spring to autumn. The countries in the Southern Hemisphere (Brazil and South Africa) showed unique seasonal patterns. Brazil exhibited twin peaks in spring and autumn and a trough in summer, which was consistent with a previous study of Sao Paulo (Bando *et al*., 2009), but differed from reports of spring or early summer peaks observed only in the south of Brazil (Benedito-Silva *et al*., 2007). South Africa exhibited unimodality with summer peak, which was consistent with an Australian study (Rock *et al*., 2003).

We observed sex-related differences in seasonal suicide patterns in Japan, the UK and the USA. Previous studies have reported a single spring peak for males and two peaks for females in the UK, Finland and Italy (Meares *et al*., 1981; Näyhä, 1982; Micciolo *et al*., 1989; Preti and Miotto, 1998), which were replicated in our study in the UK only. The peak appeared later for females in Japan, while the amplitude was larger for females in the USA. In other countries, sex-related differences were not significant. Our results were based on more up-to-date data than previous studies that relied on data collected before or about the 1980s. Recently, gender roles have been changing and social status of women has risen, possibly reducing sex differences in suicide seasonality. Also, the amplitude of seasonality was larger for the elderly population than for younger people in many countries. Although age group differences have been inconclusive in previous studies (Woo *et al*., 2012), our observation could relate to the temperature–suicide association reported as larger in elderly populations in some countries (Kim *et al*., 2019; Sim *et al*., 2020). The elderly may be more sensitive to seasonal temperature changes in terms of the risk of dying by suicide. Alternatively, in spring and summer, younger generations spend more time outside rather than caring for elderly people, possibly contributing to increased suicides in the elderly in the spring or summer (Preti and Miotto, 1998).

Previous studies reported that seasonal changes in suicide have diminished since the 19th century and, eventually, may even disappear (Yip *et al*., 2000; Ajdacic-Gross *et al*., 2005; Bramness *et al*., 2015). A plausible explanation for this trend was that the urbanisation proceeded and people in urban areas are less sensitive to seasonal changes that increase suicide risks (Ajdacic-Gross *et al*., 2005, 2010). However, our results indicate that temporal changes varied by country. In Canada, the spring peak decreased after the year 2000, consistent with previous results showing decreased seasonality in European countries (Oravecz *et al*., 2006; Bramness *et al*., 2015). Meanwhile, peaks increased in Spain, Taiwan and the UK after 2000, consistent with previous studies showing increasing seasonality in countries such as the USA, Ireland and Australia (Rock *et al*., 2003; Casey *et al*., 2012; Schwartz, 2019). The other countries showed little change over the periods, consistent with previous evidence that seasonality did not decrease for Austria and China (Nader *et al*., 2011; Sun *et al*., 2011). These suggest that the temporal change in seasonal suicide patterns is specific to each country, possibly related to country-specific macroeconomic indicators that fluctuate over a long period of time. No previous study investigated the temporal change in suicide seasonality relating to those indicators, for which further research is merited.

The amplitude of seasonality was larger for the communities with colder climates, higher proportions of the elderly and lower unemployment rates. This finding aligns with literature suggesting a latitudinal effect on seasonal suicide rhythms (Benedito-Silva *et al*., 2007; Schwartz, 2019), though specific causes of this latitudinal effect remain unknown. From a climatic perspective, larger seasonality in colder regions may relate to acclimatisation, as people living in colder areas are less adapted to warmer seasons (i.e. spring in the Northern Hemisphere) and more sensitive to seasonal temperature changes that may increase suicide risks. Such acclimatisation has been observed in the studies that investigated the association between temperature and all-cause mortality (Chung *et al*., 2018; Sera *et al*., 2019) and the temperature–suicide association also (Kim *et al*., 2019; Sim *et al*., 2020). In addition, larger seasonality in communities with higher proportions of elderly people is consistent with larger amplitudes observed for older age groups in most countries. Alternatively, it may reflect the urban/rural difference in seasonal suicide (Durkheim, 1897; Gabennesch, 1988; Chew and McCleary, 1995; Ajdacic-Gross *et al*., 2005; Sun *et al*., 2011) as a higher proportion of the elderly is one of the rural characteristics. In rural areas, people are typically engaged in agricultural activities and the intensity of social stress tends to fluctuate seasonally, while urban people may be more resilient to seasonal changes. Finally, higher unemployment rates may increase the overall suicide rate, which may decrease the seasonal pattern because people who are depressed tend to die by suicide regardless of season.

The current study investigated a broad definition of the seasonality of suicide. However, as different mechanisms (e.g. bioclimatic, socio-psychiatric, physical or others) possibly contribute simultaneously to seasonal suicide patterns, it would be more informative to decompose overall seasonal fluctuations into different mechanistic components. However, no previous study has examined how seasonal fluctuations are decomposed. One way would be to examine a residual seasonal pattern after controlling for bioclimate factors such as temperature or sunshine. Such residual seasonality, if observed, represents the portion driven by non-bioclimatic mechanisms, possibly socio-psychiatric or others. Identifying components that contribute to seasonal fluctuations the most and are potentially treatable would help reduce future suicide burdens (Woo *et al*., 2012).

Our results provide important public health implications for suicide prevention. In countries such as Brazil, South Africa, Japan, South Korea, Taiwan and the USA, a large portion of the weekly suicide counts was explained by seasonal effects (eFig. 2). Also, in these countries, the amplitude of the seasonality was relatively larger, except for the USA (Fig. 3), and higher suicide rates than the global average have been reported as 11.6–26.9 per 100 000 people in 2016 (WHO, 2016) except for Brazil. Therefore, suicide prevention strategies targeting the peak seasons and vulnerable subgroups (e.g. elderly people, females, regions with colder climates or lower unemployment rates), would help reduce the seasonal and overall suicide burden. As an example, Japan has enacted a ‘Suicide Prevention Week’ and ‘Suicide Countermeasures Strengthening Month’ as part of its suicide prevention policies, which have been helpful in promoting public awareness and in monitoring potential suicide victims (WHO, 2018). Other countries where season explains a large portion of the suicide burden may adopt similar strategies targeting specific seasons and subgroups. On the other hand, in countries such as Canada, Mexico, Romania, Switzerland, Spain and the UK, the contribution of the seasonal component to the weekly variation was relatively small, implying that targeting risk factors other than season is more important for reducing the suicide burden. Even so, Romania showed the largest amplitude (1.47) and Switzerland recorded a high suicide rate of 17.2 per 100 000 people (WHO, 2016), suggesting that seasonal prevention strategies would still help decrease seasonal suicide burdens.

We acknowledge several limitations. First, the data collection period did not precisely coincide with the collection period of community-level indicators because of the difficulty in obtaining data across different communities and countries over long periods. Fortunately, the correlation between indicators in different years was high; thus, errors that may impact risk factors should be minor (Sera *et al*., 2019). Second, the seasonality of most countries was estimated based on relatively urbanised communities, except for two countries (Japan and the UK) for which rural and urban regions across the entire country were included. The lack of rural data from certain locations may result in biased estimates for seasonality in those countries. Third, our analysis of temporal change was based on two dichotomised periods, limiting the ability to assess continuous changes over time.

## Conclusions

Our results suggest that the seasonality of suicide is largely heterogeneous across geographically, demographically and socioeconomically diverse populations. The shape and amplitude of seasonality, sex and age group differences, temporal changes and the contribution of seasonal effects to the weekly variation largely varied across countries, implying that country-specific factors modify seasonal patterns of suicide, as do community-level characteristics such as climate, demographic structure and socioeconomic condition. This study contributes to the understanding of the potential mechanisms that underlie the seasonality of suicide. Furthermore, our findings suggest that future suicide prevention programmes can be better designed by considering seasonal suicide patterns in vulnerable populations.

## Supplementary material

For supplementary material accompanying this paper visit http://dx.doi.org/10.1017/S2045796020000748. click here to view supplementary material

## Availability of data and materials

Data analysed in this study will not be available to the public. However, at any time, researchers may contact the 4th author (Masahiro Hashizume: hashizume@m.u-tokyo.ac.jp) or the principal investigator (Yeonseung Chung: dolyura@kaist.edu) for data sharing.
