# Supplementary material for: Seasonality of suicide: a multi-country multi-community observational study
Source: Epidemiol Psychiatr Sci. 2020 Aug 24;29:e163. doi: 10.1017/S2045796020000748 (PMC7503188; doi:10.1017/S2045796020000748)

**Supplemental Materials**

**Seasonality of suicide: a multi-country multi-community observational study**

**List of Contents**

**Details for Data Collection**

**Details for Adjustment of the Number of Suicides in the 53rd week**

**Details for Two-Stage Modeling**

**Details for Model Evaluation in the First Stage Modeling**

**eTable 1.** Summary statistics for the number of suicides for 354 communities

**eTable 2.** Information of community-level indicators

**eTable 3.** Country-specific summary of the seasonality of suicide

**eFigure 1.** Histogram of the weekly number of suicides for each community

**eFigure 2.** Error/Trend/Season decomposition for each country

**eFigure 3.** Community-specific RR curve from the first stage modeling

**eFigure 4.** Country-specific RR curve by different levels of community-level indicators; average temperature, unemployment rate, and the proportion of the elderly people (≥65 yrs).

**eFigure 5.** Country-specific RR curve obtained with the choice of df=2 for the cyclic spline.

**eFigure 6.** Country-specific RR curve obtained with the choice of df=3 for the cyclic spline.

**eFigure 7.** Country-specific RR curve obtained with the choice of df=5 for the cyclic spline.

**eFigure 8.** Country-specific RR curve obtained with the choice of df=6 for the cyclic spline.

**eFigure 9.** Country-specific RR curve obtained with the choice of df=7 for the cyclic spline.

**Details for Data Collection**

**Brazil**

We collected data from 15 cities from 1 January 1997 to 31 December 2005 and excluded two cities (Belem with no suicide since 2002 and Rio de Janeiro with no temperature data before 2002). A total of 13 cities were included in our study (see the full list in eTable 1). Daily suicide counts were obtained from the Ministry of Health, Brazil. Daily mean temperature and relative humidity were obtained from the National Institute of Meteorology of Brazil. The missing rates for the daily series of temperature ranged 0.0%–8.6% (0.0%–10.4% after removing stratum without suicide) across the cities.

**Canada**

We collected data from 26 cities (see the full list in eTable 1) from 1 January 1986 to 31 December 1999. Daily suicide counts were obtained from Statistics Canada. Daily mean temperature and relative humidity were obtained from Environment Canada. The missing rates for the daily series of temperature ranged 0.0%–2.4% (0.0%–3.2% after removing stratum without suicide) across the cities.

**Japan**

We collected data from 47 prefectures (see the full list in eTable 1) from 1 January 1973 to 31 December 2012. Daily suicide counts were obtained from the Ministry of Health, Labour and Welfare, Japan. Daily mean temperature and relative humidity and the daily total of sunshine duration were obtained from the Japan Meteorological Agency. The missing rates for the daily series of temperature ranged 0.0%–0.1% (0.0%–0.1% after removing stratum without suicide) across the prefectures.

**Mexico**

We collected data from 10 cities (see the full list in eTable 1) from 1 January 1998 to 31 December 2014.

**Romania**

We collected data from 8 cities (see the full list in eTable 1) from 1 January 1999 to 31 December 2016. Daily suicide data were provided by the National Institute for Statistics (NIS). Daily mean temperature and relative humidity were obtained from Romanian National Meteorological Administration (RNMA) and the daily total of sunshine duration were obtained from ROCADA 10 km resolution gridded dataset.

**South Korea**

We collected data from 6 cities (see the full list in eTable 1) from 1 January 1992 to 31 December 2013. Daily suicide counts were obtained from Statistics Korea, Ministry of Strategy and Finance in South Korea. Daily mean temperature and relative humidity and the daily total of sunshine duration were obtained from the Korea Meteorological Administration. The missing rates for the daily series of temperature ranged 0.0%–0.0% across the cities.

**South Africa**

We collected data from 52 districts from 1 January 2000 to 31 December 2013 and excluded 13 districts (6 with substantial missing values of temperature above 30% and 7 with a few suicides less than 30 for the entire study period). Therefore, a total of 39 districts were included in our study (see the full list in eTable 1). Daily suicide counts were kindly supplied by Statistics South Africa, who had no role in the data analysis or interpretation. It is likely that the suicide data was underreported^1,2^. Daily mean temperature was obtained from the Agricultural Research Council of South Africa and the National Oceanographic and Atmospheric Association (NOAA) of the United States; the latter was also the source of the daily total of sunshine duration. The missing rates for the daily series of temperature ranged 0.0%–28.9% (0.0%–22.2% after removing stratum without suicide) across the districts.

**Spain**

We collected data from 52 provincial capital cities from 1 January 1990 to 31 December 2013 and excluded two cities (Palencia with no temperature data since 1991 and Ceuta with no temperature data before 2003). A total of 50 cities were included in our study (see the full list in eTable 1). Daily suicide counts were obtained from Spain National Institute of Statistics. Daily mean temperature and the daily total of sunshine duration were obtained from Spain National Meteorology Agency. The missing rates for the daily series of temperature ranged 0.0%–16.8% (0.0%–15.2% after removing stratum without suicide) across the cities.

**Switzerland**

We collected data from 8 regions (cantons; see the full list in eTable 1) from 1 January 1995 to 31 December 2013. Daily suicide counts were obtained from the Federal Office of Statistics. The suicide data includes in part assisted suicide from 1995 to 2008. Daily mean temperature, relative humidity, and the daily total of sunshine duration were collected from the IDAWEB web database (a service provided by MeteoSwiss, the Swiss Federal Office of Meteorology and Climatology, MeteoSwiss). A single weather station within each canton was selected (Basel-Stadt: Basel/Binningen; Berne: Berne/Zollikofen;

Geneva: Genève/Cointrin; Ticino: Lugano; Lucerne: Lucerne; Vaud: Pully; St. Gallen: St. Gallen; Zurich: Zurich/Fluntern). The missing rates for the daily series of temperature ranged 0.0%–0.0% across the regions.

**Taiwan**

We collected data from 3 cities (see the full list in eTable 1) from 1 January 1994 to 31 December 2007. Daily suicide counts were obtained from the Department of Statistics, Ministry of Health and Welfare in Taiwan. Daily mean temperature, relative humidity, and the daily total of sunshine duration were obtained from the Taiwan Environmental Protection Administration. The missing rates for the daily series of temperature ranged 0.0%–0.0% across the cities.

**UK**

We collected data from 10 regions in England and Wales (see the full list in eTable 1) from 1 January 1990 to 31 December 2011. Daily suicide counts were obtained from the Office of National Statistics. Daily mean temperature and the daily total of sunshine duration were obtained from the British Atmospheric Data Centre. The missing rates for the daily series of temperature ranged 0.0%–0.0% across the regions.

**US**

We collected data from 135 cities from 1 January 2001 to 31 December 2005 and excluded Honolulu with no suicide in the study period. A total of 134 cities were included in our study (see the full list in eTable 1). Daily suicide counts were obtained from the National Center for Health Statistics. Daily mean temperature and relative humidity were obtained from the National Climate Data Center of the National Oceanic and Atmospheric Administration. The missing rates for the daily series of temperature ranged 0.0%–11.2% (0.0%–11.7% after removing stratum without suicide) across the cities.

**Details for Adjustment of the Suicide Count of the 53rd Week**

Due to the smaller number of days included in the 53rd week, we adjusted the suicide count of the 53rd week as follows.

$$Y_{53}^{*}=\left( Y_{53}+Y_{52}+Y_{1} \right)*\frac{7}{7+7+n_{53}}$$

where $Y_{53}^{*}$ is the adjusted suicide count in the 53rd week, $Y_{53}$ is the raw suicide count in the 53rd week, $Y_{52}$ is the raw suicide count in the 52nd week, $Y_{1}$ is the raw suicide count in the 1st week of the following year, and $n_{53}$ is the number of days included in the 53rd week. (i.e., $n_{53}$=1 or 2 depending on whether the current year is a leap year or not.)

**Details for Two-Stage Modeling**

*First stage modeling*

The first stage models the seasonal pattern of the number of suicides for each community. For each community, let $y_{t}$ be the weekly suicide count at time $t$, $year_{t}$ be the categorical variable indicating the year at time $t$, and $week_{t}$ be the week variable at time $t$ (taking the values from 1 to 53). We use the following generalized linear model with a quasi-Poisson distribution.

$$y_{t}\sim quasiPoisson(\lambda_{t})$$

|  | $\log\left( \lambda_{t} \right)=\alpha_{0}+factor(year_{t}) +s(week_{t};\boldsymbol{\beta})$ | (1) |
| --- | --- | --- |

where $\lambda_{t}\equiv E\left( y_{t} \right)$ is the expected weekly number of suicide at time t, $\alpha_{0}$ is an intercept, $factor(year_{t})$forms the dummy variables for indicating each of the years, and s( ) is a flexible function of week. For s$\left( \cdot\right)$, we used a cyclic B-spline with $\nu$ degree of freedom. Then, s$\left( \cdot\right)$ is expressed as follows,

|  | $s\left( week_{t};\boldsymbol{\beta} \right)=\sum_{k=1}^{\nu} \beta_{k}f_{k}\left( \mathrm{wee}k_{t} \right)$ | (2) |
| --- | --- | --- |

where $\boldsymbol{\beta}=\left( \boldsymbol{\beta}_{1},\ldots,\boldsymbol{\beta}_{\nu} \right)^{'}$ is the basis coefficient and $f_{k}\left( \mathrm{wee}k_{t} \right)$’s are the basis functions. Consequently, $\boldsymbol{\beta}$ is the parameter that represents the seasonal patterns of suicide for each community. For the value of $\nu$, we conducted model evaluation as described in the Details for Model Evaluation. For main results, we used $\nu=4$ while we conducted sensitivity analysis with $\nu=2, 3, 4, 5, 6$.

*Second stage modeling*

In the second stage, we pooled the community-specific parameter estimate for the seasonal pattern to obtain country-specific estimate. Let ${\hat{\boldsymbol{\beta}}}_{i}$ be the seasonality parameter estimate for ith community as described in the first stage modeling. To combine ${\hat{\boldsymbol{\beta}}}_{i}$ over all communities, we apply a multivariate meta-regression. Let $\hat{V}\left( {\hat{\boldsymbol{\beta}}}_{i} \right)$ be the covariance matrix for ${\hat{\boldsymbol{\beta}}}_{i}$ obtained from the first stage modeling. With $p$ meta-predictors $\boldsymbol{x}_{i}=\left( x_{i1}, \ldots,x_{ip} \right)^{'}$for $i$-th community, multivariate meta-regression model is expressed as follows:

|  | ${\hat{\boldsymbol{\beta}}}_{i}\sim N_{\nu}\left( \mathbf{X}_{i}\boldsymbol{\theta},\hat{V}\left( {\hat{\boldsymbol{\beta}}}_{i} \right)+\boldsymbol{\Psi} \right)$ | (3) |
| --- | --- | --- |
|  |  |  |

where $\mathbf{X}_{i}$ is a $\nu\times p\nu$ block-diagonal matrix derived by the Kronecker product between an identity matrix $I_{\nu}$ of dimension $\nu$ and the meta-predictor $\boldsymbol{x}_{i}$ (i.e., $\mathbf{X}_{i}=I_{\nu}\otimes\boldsymbol{x}_{i}$), $\hat{V}\left( {\hat{\boldsymbol{\beta}}}_{i} \right)$ is the within-community covariance matrix, and $\boldsymbol{\Psi}$ is the between-community covariance matrix. The $p\nu$-dimensional coefficient $\boldsymbol{\theta}$ shows the association between ${\hat{\boldsymbol{\beta}}}_{i}$’s and meta-predictors $\boldsymbol{x}_{i}$.

To obtain country-specific pooled estimates, we include the country indicators as meta-predictors (i.e. $\boldsymbol{x}_{i}=\left( x_{i1}, \ldots,x_{iN} \right)^{'}$ where $x_{\mathrm{ij}}=1$if $i$-th community belongs the j-th country and $x_{\mathrm{ij}}=0$, otherwise). The $N$ is the total number of countries. Then, $\boldsymbol{\theta}$ contains country-specific pooled coefficients to represent the seasonal pattern. Using the estimate of $\boldsymbol{\theta}$ and the basis $f_{k}\left( \mathrm{wee}k_{t} \right)$, we can estimate the relative risk (RR) curve for each country as described in the following.

Additionally, to investigate the impact of meta-predictors on the seasonal pattern of suicide, we include the country indicators and all other community-level indicators (i.e., meta-predictors) in $\boldsymbol{x}_{i}$. To evaluate the statistical significance of the meta-predictors, we used the stepwise variable selection method starting with a minimal model that includes country indicators only.

*Estimating the RR curve*

Using the country-specific pooled coefficient estimate from the second stage, we can obtain the RR curve to represent the seasonal pattern of suicide for each country. Let ${\hat{\boldsymbol{\theta}}}^{(j)}$ be a $\nu$-dimensional vector and ${\hat{\boldsymbol{V}}}^{(j)}\left( \boldsymbol{\theta} \right)$ be a $\nu\times\nu$ covariance matrix that corresponds to ${\hat{\boldsymbol{\theta}}}^{(j)}$. First, we generate cyclic B-spline basis functions with $\nu$ degree of freedom, $f_{k}\left( week_{t} \right)$, $k=1,\ldots, \nu$ for $week_{t}=1, 2, \ldots, 53$. Then, we calculate the minimum of RR for $j$-th country, denoted by $week_{t}^{j*}$ as follows:

|  | $week_{t}^{j*}={argmin}_{week_{t}}( \sum_{k=1}^{\nu} \theta_{k}^{(j)}f_{k}\left( week_{t} \right))$ | (4) |
| --- | --- | --- |

where ${\hat{\boldsymbol{\theta}}}^{(j)}=(\theta_{1}^{\left( j \right)},\ldots,\theta_{\nu}^{(j)})$. Then, we can obtain RR at $week_{t}$ for $j$-th country as follows:

|  | $RR^{j}(week_{t})=exp( \sum_{k=1}^{\nu} \theta_{k}^{\left( j \right)}f_{k}\left( week_{t} \right)- \sum_{k=1}^{\nu} \theta_{k}^{\left( j \right)}f_{k}\left( week_{t}^{j*} \right) )$ | (5) |
| --- | --- | --- |

To obtain the confidence interval of RR at $week_{t}$ for $j$-th country, we calculate standard error of RR at $week_{t}$ for $j$-th country, denoted by ${sd}^{j}({week}_{t})$ as follows:

|  | ${sd}^{j}\left( {week}_{t} \right)=\left( \boldsymbol{f}_{\boldsymbol{t}}^{\mathbf{j}} \right)^{'}{\hat{\boldsymbol{V}}}^{(j)}\left( \boldsymbol{\theta} \right) \boldsymbol{f}_{\boldsymbol{t}}^{\mathbf{j}}$ | (6) |
| --- | --- | --- |

where $\boldsymbol{f}_{\boldsymbol{t}}^{\mathbf{j}}=\left( f_{1}\left( week_{t} \right), \ldots,f_{\nu}\left( week_{t} \right) \right)^{'}- \left( f_{1}\left( week_{t}^{j*} \right), \ldots,f_{\nu}\left( week_{t}^{j*} \right) \right)^{'}$. Then, the the confidence interval of RR at $week_{t}$ for $j$-th country is $( RR^{j}\left( week_{t} \right)-1.96\cdot{sd}^{j}\left( {week}_{t} \right), RR^{j}\left( week_{t} \right)+1.96\cdot{sd}^{j}\left( {week}_{t} \right) )$.

**Details for Model Evaluation in the First Stage Modeling**

In order to select the optimal degree of freedom for the cyclic spline, we conducted model evaluation based on the likelihood ratio test (LRT) and Quasi-Akaike Information Criterion (QAIC). We considered different degrees of freedom (df) by allocating different numbers of knots. First, the locations of seven knots were determined such that they are equally spaced over the interval from 1 to 53, which led to the cyclic spline with df=7. Then, the cyclic splines with df less than 7 were formulated by removing the knots one by one to ensure the nested structure so that the LRT test is valid. We considered the largest community (i.e., the community with the largest average weekly suicide counts) of each country for model evaluation because the statistical uncertainty (i.e., standard errors) in estimating the seasonal pattern was smaller in large locations. They include Sao Paulo, Toronto, Tokyo, Valley of Mexico, Bucuresti, City of Cape Town, Seoul, Barcelona, Zurich, Taipei, South East, and Los Angeles. For each of these communities, we fit the first stage model with different df for the cyclic spline and conducted the LRT for the pairs of models.

**Table. The allocation of the knots for different degrees of freedom**

| **Degrees of freedom** | **Allocation of the knots** |
| --- | --- |
| 2 | 1st, 7th |
| 3 | 1st, 5th, 7th |
| 4 | 1st, 3rd, 5th, 7th |
| 5 | 1st, 2nd, 3rd, 5th, 7th |
| 6 | 1st, 2nd, 3rd, 5th, 6th, 7th |
| 7 | 1st, 2nd, 3rd, 4th, 5th, 6th, 7th equally spaced knots |

**Table. p-values from the likelihood ratio test for comparing the models with different df**

| **Country** | **City** | **Models Compared** | | | | |
| --- | --- | --- | --- | --- | --- | --- |
|  |  | **df=2 vs 3** | **df=3 vs 4** | **df=4 vs 5** | **df=5 vs 6** | **df=6 vs 7** |
| Brazil | Sao Paulo | 0.9337 | **0.0394** | 0.8918 | 0.7377 | 0.7164 |
| Canada | Toronto | 0.7067 | 0.1820 | 0.2614 | 0.5457 | 0.3875 |
| Japan | Tokyo | **<0.0001** | 0.1035 | **0.0005** | 0.1027 | 0.1146 |
| Mexico | Valley of Mexico | **0.0003** | 0.4131 | 0.0681 | 0.8338 | 0.1362 |
| Romania | Bucuresti | 0.6761 | 0.4414 | 0.172 | **0.0222** | 0.0784 |
| South Africa | City of Cape Town | 0.7570 | **0.0377** | 0.1037 | 0.4896 | **0.0344** |
| South Korea | Seoul | **<0.0001** | 0.0636 | 0.2538 | 0.0739 | 0.4112 |
| Spain | Barcelona | 0.4494 | 0.4889 | 0.1165 | 0.7252 | 0.1333 |
| Switzerland | Zurich | 0.5845 | 0.5179 | 0.5692 | 0.2879 | 0.9038 |
| Taiwan | Taipei | **0.0261** | 0.3366 | 0.1845 | 0.6413 | 0.5112 |
| UK | South East | 0.2471 | 0.7092 | 0.2127 | 0.8266 | 0.4693 |
| US | Los Angeles | 0.5218 | 0.0537 | 0.2397 | 0.4056 | 0.8980 |

The above table showed the p-values of the LRT test for each community. We considered p-value<0.05 as statistically significant. For Toronto, Barcelona, Zurich, South East, and Los Angeles, df=2 seems to be optimal as none of the higher df was found to be significant. For Valley of Mexico, Seoul, and Taipei, df=3 was found to be optimal. For Sau Paulo, Tokyo, Bucuresti, and City of Cape Town, df=4, 5, 6, and 7 were found to be optimal, respectively.

In addition to the LRT, we calculated the QAIC for each df for each community as presented in the following figure. For Tokyo, Valley of Mexico, Seoul, and Taipei, the model with df=2 yielded the smallest QAIC. For Sao Paulo, Toronto, and Los Angeles, the model with df=3 yielded the smallest QAIC. For the other communities including Bucuresti, City of Cape Town, Barcelona, Zurich, and South East, the model with df≥4 yielded the smallest QAIC.

**Figure. QAIC calculated in the first stage model with different df for cyclic spline for each community**

We found that there was no unique choice of the df agreed by the LRT and QAIC for all communities. Therefore, we determined df=4 as the optimal choice because the model with df=4 led to sufficiently flexible models to capture the nonlinear and cyclic seasonal patterns of suicide while ensuring the interpretability of the results in all communities.

**eTable 1.** Summary statistics for the number of suicides for 354 communities

| Community | Country | Study period | Total number of suicides | Average number of weekly suicides | Total number of male suicides (%) | Total number of non-elderly suicides (%) |
| --- | --- | --- | --- | --- | --- | --- |
| Maceio | Brazil | 1997-2005 | 169 | 0.35 | 84.02 | 93.49 |
| Manaus | Brazil | 1997-2005 | 442 | 0.92 | 81.22 | 96.83 |
| Salvador | Brazil | 1997-2005 | 178 | 0.37 | 70.79 | 89.33 |
| Fortaleza | Brazil | 1997-2005 | 886 | 1.87 | 80.02 | 93.91 |
| Brasilia | Brazil | 1997-2005 | 622 | 1.30 | 77.81 | 94.69 |
| Vitoria | Brazil | 1997-2005 | 218 | 0.46 | 76.61 | 96.79 |
| Belo Horizonte | Brazil | 1997-2005 | 843 | 1.77 | 74.61 | 94.42 |
| Campo Grande | Brazil | 1997-2005 | 266 | 0.56 | 81.2 | 91.73 |
| Teresina | Brazil | 1997-2005 | 301 | 0.63 | 70.43 | 94.02 |
| Curitiba | Brazil | 1997-2005 | 635 | 1.32 | 75.43 | 91.02 |
| Natal | Brazil | 1997-2005 | 140 | 0.29 | 72.86 | 95 |
| Porto Alegre | Brazil | 1997-2005 | 800 | 1.67 | 77.5 | 90.75 |
| Sao Paulo | Brazil | 1997-2005 | 3301 | 6.98 | 77.01 | 92.12 |
| Vancouver | Canada | 1986-2015 | 2450 | 3.44 | 76.45 | 83.59 |
| Victoria | Canada | 1986-2015 | 496 | 0.69 | 73.19 | 76.81 |
| Abbotsford | Canada | 1986-2015 | 253 | 0.45 | 80.63 | 88.14 |
| Edmonton | Canada | 1986-2015 | 2290 | 2.89 | 74.54 | 91.14 |
| Calgary | Canada | 1986-2015 | 1656 | 2.40 | 78.44 | 91.91 |
| Saskatoon | Canada | 1986-2015 | 456 | 0.59 | 77.85 | 91.01 |
| Regina | Canada | 1986-2015 | 426 | 0.54 | 81.46 | 90.14 |
| Winnipeg | Canada | 1986-2015 | 1284 | 1.71 | 76.79 | 84.58 |
| Kitchener - Waterloo | Canada | 1986-2015 | 426 | 0.68 | 79.58 | 84.51 |
| Toronto | Canada | 1986-2015 | 4896 | 6.65 | 71.9 | 84.33 |
| Thunder bay | Canada | 1986-2015 | 328 | 0.42 | 84.76 | 84.45 |
| Sudbury | Canada | 1986-2015 | 429 | 0.48 | 81.82 | 86.71 |
| Windsor | Canada | 1986-2015 | 524 | 0.62 | 76.53 | 85.88 |
| London | Canada | 1986-2015 | 581 | 0.75 | 73.32 | 85.89 |
| Kingston | Canada | 1986-2015 | 313 | 0.41 | 81.15 | 88.5 |
| Hamilton | Canada | 1986-2015 | 536 | 0.76 | 73.32 | 86.19 |
| Ottawa | Canada | 1986-2015 | 1281 | 1.45 | 73.38 | 87.28 |
| Montreal | Canada | 1992-2015 | 4022 | 4.83 | 75.14 | 89.93 |
| Saint_john_NB | Canada | 1986-2015 | 276 | 0.36 | 81.52 | 91.3 |
| Halifax | Canada | 1986-2015 | 566 | 0.72 | 79.33 | 88.87 |
| St_john_NFL | Canada | 1986-2015 | 275 | 0.40 | 84 | 86.55 |
| Oakville | Canada | 1986-2015 | 384 | 0.57 | 75.26 | 81.77 |
| Oshawa | Canada | 1986-2015 | 453 | 0.72 | 76.16 | 86.31 |
| Sault_Ste_Marie | Canada | 1986-2015 | 218 | 0.27 | 76.15 | 87.61 |
| Sarnia | Canada | 1986-2015 | 179 | 0.25 | 85.47 | 83.24 |
| Niagara | Canada | 1986-2015 | 621 | 0.79 | 78.26 | 80.84 |
| Hokkaido | Japan | 1986-2012 | 33874 | 23.98 | 69.67 | 74.64 |
| Aomori | Japan | 1986-2012 | 10987 | 7.77 | 72.33 | 71.17 |
| Iwate | Japan | 1986-2012 | 11335 | 8.01 | 68.31 | 65.2 |
| Miyagi | Japan | 1986-2012 | 12942 | 9.15 | 70.17 | 74.94 |
| Akita | Japan | 1986-2012 | 10763 | 7.60 | 66.49 | 63.11 |
| Yamagata | Japan | 1986-2012 | 8311 | 5.87 | 68.01 | 64.79 |
| Fukushima | Japan | 1986-2012 | 12870 | 9.11 | 69.06 | 70.53 |
| Ibaraki | Japan | 1986-2012 | 16325 | 11.56 | 69.24 | 74.59 |
| Tochigi | Japan | 1986-2012 | 12072 | 8.54 | 67.75 | 71.1 |
| Gunma | Japan | 1986-2012 | 12280 | 8.69 | 67.1 | 68.36 |
| Saitama | Japan | 1986-2012 | 35032 | 24.81 | 67.57 | 76.72 |
| Chiba | Japan | 1986-2012 | 28822 | 20.41 | 69.34 | 76.99 |
| Tokyo | Japan | 1986-2012 | 63075 | 44.65 | 67.24 | 78.65 |
| Kanagawa | Japan | 1986-2012 | 40420 | 28.63 | 69.13 | 78.62 |
| Niigata | Japan | 1986-2012 | 19014 | 13.45 | 65.45 | 62.63 |
| Toyama | Japan | 1986-2012 | 7392 | 5.23 | 66.26 | 66.19 |
| Ishikawa | Japan | 1986-2012 | 6308 | 4.46 | 69.51 | 73.49 |
| Fukui | Japan | 1986-2012 | 4521 | 3.19 | 69.08 | 66.8 |
| Yamanashi | Japan | 1986-2012 | 5271 | 3.72 | 70.4 | 72.95 |
| Nagano | Japan | 1986-2012 | 12733 | 9.01 | 66.13 | 68.28 |
| Gifu | Japan | 1986-2012 | 11607 | 8.20 | 65.4 | 67.39 |
| Shizuoka | Japan | 1986-2012 | 18483 | 13.09 | 70.65 | 73.06 |
| Aichi | Japan | 1986-2012 | 33810 | 23.94 | 67.23 | 73.87 |
| Mie | Japan | 1986-2012 | 9242 | 6.54 | 66.81 | 69.76 |
| Shiga | Japan | 1986-2012 | 6601 | 4.67 | 67.87 | 71.82 |
| Kyoto | Japan | 1986-2012 | 13629 | 9.65 | 66.59 | 73.84 |
| Osaka | Japan | 1986-2012 | 48795 | 34.55 | 69.38 | 77.13 |
| Hyogo | Japan | 1986-2012 | 29738 | 21.04 | 67.81 | 74.14 |
| Nara | Japan | 1986-2012 | 6530 | 4.62 | 66.13 | 72.62 |
| Wakayama | Japan | 1986-2012 | 6851 | 4.85 | 66.63 | 67.57 |
| Tottori | Japan | 1986-2012 | 3687 | 2.59 | 70.82 | 70.14 |
| Shimane | Japan | 1986-2012 | 5599 | 3.96 | 71.1 | 65.48 |
| Okayama | Japan | 1986-2012 | 9702 | 6.86 | 69.32 | 73.37 |
| Hiroshima | Japan | 1986-2012 | 15436 | 10.91 | 68.88 | 72.71 |
| Yamaguchi | Japan | 1986-2012 | 9432 | 6.68 | 69.55 | 69.56 |
| Tokushima | Japan | 1986-2012 | 4176 | 2.95 | 66.81 | 69.35 |
| Kagawa | Japan | 1986-2012 | 5324 | 3.77 | 68.63 | 73.33 |
| Ehime | Japan | 1986-2012 | 8907 | 6.31 | 68.73 | 72.58 |
| Kochi | Japan | 1986-2012 | 5522 | 3.91 | 71.01 | 69.45 |
| Fukuoka | Japan | 1986-2012 | 29373 | 20.81 | 71.91 | 76.47 |
| Saga | Japan | 1986-2012 | 5048 | 3.57 | 73.22 | 74.5 |
| Nagasaki | Japan | 1986-2012 | 8873 | 6.29 | 72.37 | 73.9 |
| Kumamoto | Japan | 1986-2012 | 10913 | 7.73 | 70.46 | 71.63 |
| Oita | Japan | 1986-2012 | 7121 | 5.04 | 69.37 | 70.79 |
| Miyazaki | Japan | 1986-2012 | 8565 | 6.06 | 69.62 | 66.77 |
| Kagoshima | Japan | 1986-2012 | 11741 | 8.31 | 70.33 | 68.76 |
| Okinawa | Japan | 1986-2012 | 7788 | 5.51 | 77.95 | 84.63 |
| Guadalajara | Mexico | 1998-2014 | 3910 | 4.41 | 81.69 | 94.35 |
| Ciudad Juarez | Mexico | 1998-2014 | 1200 | 1.34 | 85.17 | 93.75 |
| Comarca Lagunera | Mexico | 1998-2014 | 858 | 0.96 | 85.78 | 91.14 |
| Leon | Mexico | 1998-2014 | 1315 | 1.48 | 83.42 | 96.12 |
| Monterrey | Mexico | 1998-2014 | 2936 | 3.29 | 84.20 | 91.69 |
| Puebla-Tlaxcala | Mexico | 1998-2014 | 1831 | 2.06 | 76.79 | 93.66 |
| San Luis Potosi | Mexico | 1998-2014 | 910 | 1.02 | 82.53 | 94.51 |
| Tijuana | Mexico | 1998-2014 | 985 | 1.10 | 87.51 | 95.43 |
| Toluca de Lerdo | Mexico | 1998-2014 | 726 | 0.81 | 75.07 | 95.18 |
| Valley of Mexico | Mexico | 1998-2014 | 11658 | 13.14 | 79.12 | 93.12 |
| Brasov | Romania | 1999-2016 | 534 | 0.56 | 81.65 | 79.59 |
| Bucuresti | Romania | 1999-2016 | 1841 | 1.94 | 81.15 | 76.32 |
| Cluj-Napoca | Romania | 1999-2016 | 485 | 0.51 | 77.53 | 78.97 |
| Constanta | Romania | 1999-2016 | 573 | 0.60 | 77.31 | 78.01 |
| Craiova | Romania | 1999-2016 | 374 | 0.39 | 74.06 | 79.68 |
| Galati | Romania | 1999-2016 | 541 | 0.57 | 82.26 | 84.66 |
| Iasi | Romania | 1999-2016 | 556 | 0.58 | 80.04 | 88.13 |
| Timisoara | Romania | 1999-2016 | 476 | 0.50 | 76.68 | 75.00 |
| Alfred Nzo | South Africa | 2000-2013 | 89 | 0.12 | 80.9 | 95.51 |
| Amathole | South Africa | 2000-2013 | 200 | 0.27 | 80.5 | 94.5 |
| Buffalo City | South Africa | 2000-2013 | 37 | 0.05 | 62.16 | 100 |
| Bojanala | South Africa | 2000-2013 | 69 | 0.09 | 78.26 | 95.65 |
| Chris Hani | South Africa | 2000-2013 | 59 | 0.08 | 66.1 | 88.14 |
| City of Cape Town | South Africa | 2000-2013 | 439 | 0.60 | 82.23 | 93.85 |
| Capricorn | South Africa | 2000-2013 | 30 | 0.04 | 53.33 | 90 |
| Cape Winelands | South Africa | 2000-2013 | 85 | 0.11 | 76.47 | 96.47 |
| City of Johannesburg | South Africa | 2000-2013 | 74 | 0.10 | 58.11 | 90.54 |
| City of Tshwane | South Africa | 2000-2013 | 117 | 0.16 | 82.05 | 94.02 |
| Dr Kenneth Kaunda | South Africa | 2000-2013 | 60 | 0.08 | 75 | 88.33 |
| Dr Ruth Segomotsi Mompati | South Africa | 2000-2013 | 35 | 0.05 | 74.29 | 97.14 |
| Eden | South Africa | 2000-2013 | 174 | 0.24 | 76.44 | 93.1 |
| Ehlanzeni | South Africa | 2000-2013 | 255 | 0.34 | 80.78 | 90.59 |
| Ekurhuleni | South Africa | 2000-2013 | 49 | 0.07 | 71.43 | 93.88 |
| eThekwini | South Africa | 2000-2013 | 96 | 0.13 | 70.83 | 96.88 |
| Frances Baard | South Africa | 2000-2013 | 135 | 0.18 | 69.63 | 95.56 |
| Gert Sibande | South Africa | 2000-2013 | 197 | 0.27 | 83.76 | 94.92 |
| Joe Gqabi | South Africa | 2000-2013 | 45 | 0.06 | 75.56 | 91.11 |
| Lejweleputswa | South Africa | 2000-2013 | 130 | 0.18 | 81.54 | 90.77 |
| Mangaung | South Africa | 2000-2013 | 113 | 0.15 | 84.07 | 96.46 |
| Mopani | South Africa | 2000-2013 | 293 | 0.39 | 77.47 | 84.98 |
| Ngaka Modiri Molema | South Africa | 2000-2013 | 240 | 0.32 | 87.08 | 93.75 |
| Nkangala | South Africa | 2000-2013 | 65 | 0.09 | 81.54 | 89.23 |
| Nelson Mandela Bay | South Africa | 2000-2013 | 149 | 0.20 | 73.83 | 95.3 |
| Namakwa | South Africa | 2000-2013 | 47 | 0.06 | 91.49 | 100 |
| O.R.Tambo | South Africa | 2000-2013 | 59 | 0.08 | 61.02 | 89.83 |
| Overberg | South Africa | 2000-2013 | 73 | 0.10 | 87.67 | 97.26 |
| Pixley ka Seme | South Africa | 2000-2013 | 77 | 0.11 | 80.52 | 96.1 |
| Sisonke | South Africa | 2000-2013 | 326 | 0.45 | 81.6 | 96.63 |
| Siyanda | South Africa | 2000-2013 | 312 | 0.43 | 72.44 | 96.15 |
| Thabo Mofutsanyane | South Africa | 2000-2013 | 46 | 0.06 | 80.43 | 93.48 |
| Ugu | South Africa | 2000-2013 | 110 | 0.15 | 69.09 | 96.36 |
| uMgungundlovu | South Africa | 2000-2013 | 175 | 0.24 | 81.14 | 97.14 |
| uMkhanyakude | South Africa | 2000-2013 | 90 | 0.12 | 67.78 | 92.22 |
| uMzinyathi | South Africa | 2000-2013 | 208 | 0.28 | 76.44 | 96.15 |
| uThukela | South Africa | 2000-2013 | 58 | 0.08 | 75.86 | 89.66 |
| uThungulu | South Africa | 2000-2013 | 274 | 0.37 | 76.64 | 94.53 |
| West Coast | South Africa | 2000-2013 | 38 | 0.05 | 81.58 | 97.37 |
| Seoul | South Korea | 1992-2013 | 35817 | 31.09 | 66.61 | 79.14 |
| BUSn | South Korea | 1992-2013 | 16111 | 13.98 | 69.28 | 79.03 |
| Incheon | South Korea | 1992-2013 | 11535 | 10.01 | 68.31 | 76.31 |
| Daegu | South Korea | 1992-2013 | 9637 | 8.36 | 66.37 | 80.63 |
| Daejeon | South Korea | 1992-2013 | 5812 | 5.05 | 66.12 | 78.18 |
| Gwangju | South Korea | 1992-2013 | 4913 | 4.26 | 66.17 | 82.9 |
| Almeria | Spain | 1990-2013 | 321 | 0.25 | 76.95 | 73.21 |
| Cadiz | Spain | 1990-2013 | 242 | 0.19 | 69.42 | 75.62 |
| Cordoba | Spain | 1990-2013 | 489 | 0.39 | 74.85 | 75.66 |
| Granada | Spain | 1990-2013 | 520 | 0.41 | 70 | 74.04 |
| Huelva | Spain | 1990-2013 | 186 | 0.14 | 68.28 | 76.34 |
| Jaen | Spain | 1990-2013 | 233 | 0.18 | 74.68 | 69.53 |
| Malaga | Spain | 1990-2013 | 1113 | 0.88 | 71.88 | 71.34 |
| Sevilla | Spain | 1990-2013 | 1173 | 0.93 | 71.78 | 72.21 |
| Huesca | Spain | 1990-2013 | 103 | 0.08 | 74.76 | 55.34 |
| Teruel | Spain | 1990-2013 | 67 | 0.05 | 79.1 | 50.75 |
| Zaragoza | Spain | 1990-2013 | 1076 | 0.85 | 68.59 | 65.33 |
| Oviedo | Spain | 1990-2013 | 517 | 0.41 | 66.15 | 58.22 |
| Mallorca | Spain | 1990-2013 | 553 | 0.44 | 80.11 | 75.95 |
| LasPalmas | Spain | 1990-2013 | 671 | 0.53 | 77.2 | 80.48 |
| Tenerife | Spain | 1990-2013 | 337 | 0.26 | 76.85 | 78.04 |
| Santander | Spain | 1990-2013 | 207 | 0.16 | 76.81 | 73.43 |
| Albacete | Spain | 1990-2013 | 227 | 0.18 | 72.69 | 74.01 |
| CiudadReal | Spain | 1990-2013 | 88 | 0.07 | 76.14 | 69.32 |
| Cuenca | Spain | 1990-2013 | 87 | 0.07 | 78.16 | 68.97 |
| Guadalajara | Spain | 1990-2013 | 94 | 0.07 | 68.09 | 69.15 |
| Toledo | Spain | 1990-2013 | 82 | 0.06 | 68.29 | 69.51 |
| Avila | Spain | 1990-2013 | 63 | 0.05 | 84.13 | 71.43 |
| Burgos | Spain | 1990-2013 | 308 | 0.24 | 73.7 | 72.73 |
| Leon | Spain | 1990-2013 | 223 | 0.17 | 69.06 | 67.71 |
| Salamanca | Spain | 1990-2013 | 242 | 0.19 | 67.77 | 73.14 |
| Segovia | Spain | 1990-2013 | 78 | 0.06 | 78.21 | 53.85 |
| Soria | Spain | 1990-2013 | 105 | 0.08 | 74.29 | 51.43 |
| Valladolid | Spain | 1990-2013 | 605 | 0.48 | 71.74 | 67.27 |
| Zamora | Spain | 1990-2013 | 110 | 0.09 | 71.82 | 55.45 |
| Barcelona | Spain | 1990-2013 | 2578 | 2.05 | 68.81 | 68.43 |
| Girona | Spain | 1990-2013 | 117 | 0.09 | 76.07 | 68.38 |
| Lleida | Spain | 1990-2013 | 238 | 0.19 | 72.27 | 67.23 |
| Tarragona | Spain | 1990-2013 | 226 | 0.18 | 74.34 | 75.22 |
| Melilla | Spain | 1990-2013 | 66 | 0.05 | 83.33 | 78.79 |
| Badajoz | Spain | 1990-2013 | 141 | 0.11 | 73.05 | 82.98 |
| Caceres | Spain | 1990-2013 | 99 | 0.08 | 78.79 | 70.71 |
| ACoruna | Spain | 1990-2013 | 532 | 0.42 | 67.86 | 69.17 |
| Lugo | Spain | 1990-2013 | 228 | 0.18 | 71.49 | 64.47 |
| Ourense | Spain | 1990-2013 | 233 | 0.18 | 63.52 | 66.09 |
| Pontevedra | Spain | 1990-2013 | 133 | 0.10 | 75.19 | 69.92 |
| Logrono | Spain | 1990-2013 | 296 | 0.23 | 46.28 | 40.2 |
| Madrid | Spain | 1990-2013 | 2180 | 1.73 | 73.76 | 71.79 |
| Murcia | Spain | 1990-2013 | 585 | 0.46 | 75.73 | 75.9 |
| Pamplona | Spain | 1990-2013 | 413 | 0.32 | 69.49 | 74.82 |
| Bilbao | Spain | 1990-2013 | 662 | 0.52 | 69.03 | 69.64 |
| SanSebastian | Spain | 1990-2013 | 344 | 0.27 | 66.28 | 65.7 |
| Vitoria | Spain | 1990-2013 | 447 | 0.35 | 75.84 | 69.57 |
| Alicante | Spain | 1990-2013 | 585 | 0.46 | 71.11 | 69.91 |
| Castellon | Spain | 1990-2013 | 317 | 0.25 | 68.14 | 59.31 |
| Valencia | Spain | 1990-2013 | 1458 | 1.16 | 65.91 | 72.57 |
| Zurich | Switzerland | 1995-2013 | 4577 | 4.60 | 66.46 | 62.4 |
| Berne | Switzerland | 1995-2013 | 3366 | 3.40 | 72.91 | 66.25 |
| Lucerne | Switzerland | 1995-2013 | 1019 | 1.02 | 72.23 | 75.07 |
| Basel-Stadt | Switzerland | 1995-2013 | 1435 | 1.43 | 69.06 | 63.9 |
| St. Gallen | Switzerland | 1995-2013 | 1533 | 1.54 | 73.97 | 73.65 |
| Ticino | Switzerland | 1995-2013 | 670 | 0.67 | 71.04 | 67.76 |
| Vaud | Switzerland | 1995-2013 | 2081 | 2.08 | 68.96 | 67.95 |
| Geneva | Switzerland | 1995-2013 | 1341 | 1.34 | 59.96 | 67.64 |
| Taipei | Taiwan | 1994-2007 | 9481 | 12.98 | 66.52 | 78.83 |
| Taichung | Taiwan | 1994-2007 | 3352 | 4.57 | 68.91 | 79.56 |
| Kaohsiung | Taiwan | 1994-2007 | 5050 | 6.90 | 68.06 | 77.84 |
| East | UK | 1990-2011 | 7502 | 6.53 | 77.15 | 80.3 |
| East Midlands | UK | 1990-2011 | 6120 | 5.33 | 78.89 | 83.35 |
| London | UK | 1990-2011 | 8976 | 7.79 | 72.85 | 84.08 |
| North East | UK | 1990-2011 | 3806 | 3.31 | 78.9 | 85.52 |
| North West | UK | 1990-2011 | 11074 | 9.64 | 78.6 | 85.76 |
| South East | UK | 1990-2011 | 12089 | 10.50 | 75.44 | 81.07 |
| South West | UK | 1990-2011 | 8011 | 6.97 | 76.07 | 80.38 |
| Wales | UK | 1990-2011 | 5329 | 4.63 | 80.39 | 84.67 |
| West Midlands | UK | 1990-2011 | 7438 | 6.47 | 78.69 | 82.99 |
| Yorkshire & Humber | UK | 1990-2011 | 7770 | 6.76 | 78.67 | 84.18 |
| Akron, OH | US | 2001-2006 | 367 | 1.16 | 76.29 | 83.65 |
| Albuquerque, NM | US | 2001-2006 | 620 | 1.97 | 79.52 | 84.68 |
| Allentown-Bethlehem, PA | US | 2001-2006 | 227 | 0.72 | 81.5 | 85.9 |
| Atlanta, GA | US | 2001-2006 | 1603 | 5.10 | 78.54 | 88.02 |
| Atlantic City, NJ | US | 2001-2006 | 134 | 0.43 | 78.36 | 83.58 |
| Austin, TX | US | 2001-2006 | 605 | 1.92 | 73.06 | 87.44 |
| Bakersfield, CA | US | 2001-2006 | 419 | 1.32 | 79.24 | 82.1 |
| Baltimore, MD | US | 2001-2006 | 731 | 2.33 | 81.53 | 83.72 |
| Barnstable-Yarmouth, MA | US | 2001-2006 | 120 | 0.38 | 77.5 | 82.5 |
| Bergen-Passaic, NJ | US | 2001-2006 | 474 | 1.50 | 76.79 | 77.85 |
| Birmingham, AL | US | 2001-2006 | 649 | 2.07 | 80.74 | 81.97 |
| Boston, MA | US | 2001-2006 | 957 | 3.07 | 74.82 | 86.1 |
| Baton Rouge, LA | US | 2001-2006 | 221 | 0.70 | 81 | 86.43 |
| Brownsville, TX | US | 2001-2006 | 116 | 0.37 | 79.31 | 67.24 |
| Buffalo, NY | US | 2001-2006 | 381 | 1.20 | 88.45 | 84.25 |
| Canton-Massillon, OH | US | 2001-2006 | 213 | 0.67 | 80.28 | 79.81 |
| Charleston, WV | US | 2001-2006 | 173 | 0.55 | 79.19 | 84.97 |
| Charlotte, NC | US | 2001-2006 | 420 | 1.33 | 78.1 | 86.43 |
| Chattanooga, TN | US | 2001-2006 | 206 | 0.65 | 77.67 | 85.44 |
| Chicago, IL | US | 2001-2006 | 2886 | 9.19 | 78.31 | 85.2 |
| Cincinnati, OH | US | 2001-2006 | 513 | 1.64 | 76.22 | 82.65 |
| Cleveland, OH | US | 2001-2006 | 1197 | 3.81 | 79.2 | 81.95 |
| Columbia, SC | US | 2001-2006 | 326 | 1.03 | 77.91 | 85.89 |
| Columbus, OH | US | 2001-2006 | 658 | 2.08 | 79.03 | 89.06 |
| Dallas, TX | US | 2001-2006 | 1263 | 4.01 | 76.72 | 87.25 |
| Daytona Beach, FL | US | 2001-2006 | 537 | 1.70 | 74.86 | 69.83 |
| Dayton, OH | US | 2001-2006 | 395 | 1.25 | 77.22 | 84.3 |
| Denver, CO | US | 2001-2006 | 1419 | 4.51 | 74.91 | 87.88 |
| Des Moines, IA | US | 2001-2006 | 263 | 0.83 | 78.33 | 87.45 |
| Detroit, MI | US | 2001-2006 | 2386 | 7.59 | 79.21 | 86.76 |
| Dutchess County, NY | US | 2001-2006 | 96 | 0.31 | 83.33 | 86.46 |
| El Paso, TX | US | 2001-2006 | 291 | 0.92 | 81.79 | 81.1 |
| Erie, PA | US | 2001-2006 | 158 | 0.50 | 86.71 | 84.81 |
| Flint, MI | US | 2001-2006 | 287 | 0.91 | 78.05 | 84.67 |
| Fresno, CA | US | 2001-2006 | 436 | 1.39 | 82.8 | 85.09 |
| Ft. Lauderdale, FL | US | 2001-2006 | 1267 | 4.00 | 73.8 | 81.69 |
| Fort Myers-Cape Coral, FL | US | 2001-2006 | 494 | 1.56 | 76.32 | 74.7 |
| Fort Pierce-Port St. Lucie, FL | US | 2001-2006 | 327 | 1.03 | 79.2 | 73.39 |
| Fort Worth-Arlington, TX | US | 2001-2006 | 917 | 2.91 | 77.75 | 89.42 |
| Galveston, TX | US | 2001-2006 | 208 | 0.65 | 79.33 | 87.5 |
| Gary, IN | US | 2001-2006 | 274 | 0.86 | 82.48 | 87.96 |
| Grand Rapids, MI | US | 2001-2006 | 292 | 0.93 | 78.42 | 89.38 |
| Greensboro, NC | US | 2001-2006 | 269 | 0.85 | 72.86 | 85.13 |
| Greenville, SC | US | 2001-2006 | 303 | 0.96 | 74.92 | 88.45 |
| Hamilton, OH | US | 2001-2006 | 227 | 0.71 | 77.97 | 85.9 |
| Harrisburg-Carlisle, PA | US | 2001-2006 | 140 | 0.44 | 81.43 | 86.43 |
| Hartford, CT | US | 2001-2006 | 393 | 1.24 | 79.13 | 84.22 |
| Houston, TX | US | 2001-2006 | 2072 | 6.59 | 76.5 | 87.55 |
| Indianapolis, IN | US | 2001-2006 | 618 | 1.96 | 79.29 | 89.48 |
| Jacksonville, FL | US | 2001-2006 | 674 | 2.15 | 77.15 | 85.61 |
| Jersey City, NJ | US | 2001-2006 | 162 | 0.51 | 82.72 | 82.1 |
| Kansas City, MO-KS | US | 2001-2006 | 1077 | 3.41 | 79.02 | 84.87 |
| Knoxville, TN | US | 2001-2006 | 410 | 1.31 | 80.49 | 80.49 |
| Lakeland-Winter Haven, FL | US | 2001-2006 | 389 | 1.23 | 76.61 | 79.18 |
| Lancaster, PA | US | 2001-2006 | 250 | 0.78 | 85.2 | 87.6 |
| Lansing, MI | US | 2001-2006 | 145 | 0.46 | 74.48 | 89.66 |
| Las Vegas, NV-AZ | US | 2001-2006 | 1891 | 6.06 | 78.95 | 79.32 |
| Los Angeles, CA | US | 2001-2006 | 4167 | 13.28 | 77.85 | 80.95 |
| Louisville, KY | US | 2001-2006 | 525 | 1.66 | 78.67 | 85.52 |
| Little Rock, AR | US | 2001-2006 | 272 | 0.86 | 80.88 | 90.81 |
| Lubbock, TX | US | 2001-2006 | 172 | 0.54 | 77.33 | 84.3 |
| Madison, WI | US | 2001-2006 | 300 | 0.94 | 76.67 | 89 |
| McAllen-Edinburg-Mission, TX | US | 2001-2006 | 176 | 0.55 | 86.36 | 80.11 |
| Melbourne-Titusville-Palm Bay, FL | US | 2001-2006 | 542 | 1.72 | 75.28 | 76.2 |
| Memphis, TN | US | 2001-2006 | 519 | 1.64 | 80.92 | 83.04 |
| Miami, FL | US | 2001-2006 | 1207 | 3.84 | 81.19 | 71.17 |
| Middlesex, NJ | US | 2001-2006 | 244 | 0.77 | 74.59 | 87.7 |
| Milwaukee, WI | US | 2001-2006 | 784 | 2.48 | 77.68 | 86.61 |
| Minneapolis-St. Paul, MN | US | 2001-2006 | 887 | 2.82 | 77.68 | 86.25 |
| Mobile, AL | US | 2001-2006 | 233 | 0.74 | 79.83 | 88.41 |
| Monmouth-Ocean, NJ | US | 2001-2006 | 515 | 1.64 | 80.78 | 80 |
| Myrtle Beach, SC | US | 2001-2006 | 186 | 0.58 | 79.03 | 83.87 |
| Naples, FL | US | 2001-2006 | 210 | 0.66 | 82.86 | 72.38 |
| Nashua, NH | US | 2001-2006 | 237 | 0.75 | 75.95 | 93.25 |
| Nashville, TN | US | 2001-2006 | 458 | 1.44 | 77.73 | 85.81 |
| Nassau-Suffolk, NY | US | 2001-2006 | 827 | 2.64 | 82.47 | 84.28 |
| Newark, NJ | US | 2001-2006 | 393 | 1.25 | 81.42 | 78.37 |
| Newburgh, NY | US | 2001-2006 | 125 | 0.39 | 84 | 81.6 |
| New Haven-Meriden, CT | US | 2001-2006 | 398 | 1.26 | 78.39 | 85.18 |
| New London, CT | US | 2001-2006 | 151 | 0.47 | 76.16 | 86.75 |
| New York, NY | US | 2001-2006 | 2642 | 8.42 | 73.96 | 82.51 |
| Oakland, CA | US | 2001-2006 | 1184 | 3.77 | 74.66 | 78.8 |
| Ocala, FL | US | 2001-2006 | 302 | 0.97 | 75.83 | 69.21 |
| Oklahoma City, OK | US | 2001-2006 | 570 | 1.81 | 77.37 | 87.54 |
| Omaha, NE | US | 2001-2006 | 277 | 0.87 | 81.59 | 85.92 |
| Orange County, CA | US | 2001-2006 | 1376 | 4.38 | 74.35 | 81.18 |
| Orlando, FL | US | 2001-2006 | 851 | 2.71 | 76.15 | 85.19 |
| Pensacola, FL | US | 2001-2006 | 239 | 0.76 | 76.99 | 82.01 |
| Philadelphia, PA-NJ | US | 2001-2006 | 2580 | 8.24 | 78.6 | 85.78 |
| Phoenix, AZ | US | 2001-2006 | 2744 | 8.76 | 79.41 | 83.24 |
| Pittsburgh, PA | US | 2001-2006 | 868 | 2.76 | 80.3 | 83.06 |
| Portland, ME | US | 2001-2006 | 165 | 0.53 | 78.18 | 88.48 |
| Portland, OR | US | 2001-2006 | 1132 | 3.61 | 77.03 | 86.57 |
| Providence-Fall River, RI-MA | US | 2001-2006 | 116 | 0.36 | 81.03 | 83.62 |
| Punta Gorda, FL | US | 2001-2006 | 160 | 0.50 | 76.25 | 71.25 |
| Raleigh, NC | US | 2001-2006 | 337 | 1.07 | 73 | 85.16 |
| Reading, PA | US | 2001-2006 | 271 | 0.86 | 82.66 | 80.44 |
| Riverside-San Bernardino, CA | US | 2001-2006 | 2037 | 6.52 | 79.68 | 80.51 |
| Rochester, NY | US | 2001-2006 | 277 | 0.87 | 81.23 | 83.39 |
| Rockford, IL | US | 2001-2006 | 173 | 0.54 | 77.46 | 86.13 |
| Sacramento, CA | US | 2001-2006 | 917 | 2.93 | 76.77 | 84.51 |
| Saginaw, MI | US | 2001-2006 | 125 | 0.39 | 83.2 | 85.6 |
| Salinas, CA | US | 2001-2006 | 201 | 0.64 | 74.13 | 79.6 |
| Salt Lake City, UT | US | 2001-2006 | 805 | 2.56 | 80.5 | 90.68 |
| San Antonio, TX | US | 2001-2006 | 870 | 2.76 | 78.85 | 86.78 |
| Sarasota-Bradenton, FL | US | 2001-2006 | 594 | 1.88 | 75.42 | 72.73 |
| Scranton--Wilkes-Barre--Hazleton, PA | US | 2001-2006 | 435 | 1.37 | 81.84 | 87.59 |
| San Diego, CA | US | 2001-2006 | 1758 | 5.62 | 76.96 | 78.67 |
| Seattle, WA | US | 2001-2006 | 1189 | 3.80 | 78.05 | 83.77 |
| San Francisco, CA | US | 2001-2006 | 897 | 2.84 | 77.03 | 78.93 |
| Shreveport, LA | US | 2001-2006 | 166 | 0.52 | 75.3 | 79.52 |
| San Jose, CA | US | 2001-2006 | 737 | 2.33 | 74.76 | 82.5 |
| Spokane, WA | US | 2001-2006 | 396 | 1.25 | 77.27 | 83.59 |
| Springfield, MA | US | 2001-2006 | 212 | 0.67 | 79.72 | 81.6 |
| Stamford-Norwalk, CT | US | 2001-2006 | 337 | 1.07 | 79.53 | 82.2 |
| St. Louis, MO-IL | US | 2001-2006 | 1027 | 3.28 | 76.14 | 83.15 |
| Stockton-Lodi, CA | US | 2001-2006 | 300 | 0.95 | 82 | 84.67 |
| Syracuse, NY | US | 2001-2006 | 201 | 0.64 | 82.59 | 86.57 |
| Tacoma, WA | US | 2001-2006 | 549 | 1.74 | 81.6 | 85.43 |
| Tampa-St. Petersburg-Clearwater, FL | US | 2001-2006 | 811 | 2.59 | 76.7 | 80.89 |
| Toledo, OH | US | 2001-2006 | 316 | 1.00 | 77.85 | 83.86 |
| Trenton, NJ | US | 2001-2006 | 108 | 0.34 | 82.41 | 89.81 |
| Tucson, AZ | US | 2001-2006 | 867 | 2.76 | 76.47 | 77.62 |
| Tulsa, OK | US | 2001-2006 | 507 | 1.61 | 81.07 | 85.21 |
| Utica-Rome, NY | US | 2001-2006 | 122 | 0.39 | 84.43 | 88.52 |
| Ventura County, CA | US | 2001-2006 | 445 | 1.41 | 77.75 | 80.45 |
| Virginia Beach, VA | US | 2001-2006 | 766 | 2.43 | 76.11 | 82.38 |
| Washington, DC-MD-VA | US | 2001-2006 | 244 | 0.77 | 80.33 | 84.02 |
| Wichita, KS | US | 2001-2006 | 334 | 1.05 | 80.84 | 87.43 |
| Wilmington, DE | US | 2001-2006 | 311 | 0.98 | 77.81 | 89.07 |
| Worcester, MA | US | 2001-2006 | 319 | 1.01 | 83.39 | 84.95 |
| West Palm Beach-Boca Raton, FL | US | 2001-2006 | 921 | 2.95 | 73.29 | 74.48 |
| York, PA | US | 2001-2006 | 264 | 0.84 | 85.23 | 80.3 |
| Youngstown-Warren, OH | US | 2001-2006 | 258 | 0.81 | 84.5 | 82.56 |

**eTable 2.** Information of community-level indicators

| **Indicator** | **Year** | **Number of communities** | **Countries** |
| --- | --- | --- | --- |
| Total Population | 2000 | 292 | Brazil, Canada, Japan, South Korea, Spain, Switzerland, UK, US |
| Population Density (population/km2) | 2000 | 292 | Brazil, Canada, Japan, South Korea, Spain, Switzerland, UK, US |
| Proportion of people aged over 65 years (%) | 2000 | 292 | Brazil, Canada, Japan, South Korea, Spain, Switzerland, UK, US |
| Life expectancy (years) | 2005-06; 2010-11 | 280 | Canada, Japan, South Korea, Spain, Switzerland, UK, US |
| GDP (US$) | 2001; 2010 | 331 | Brazil, Canada, Japan, South Africa, South Korea, Spain, Switzerland, UK, US |
| Unemployment rate (%) | 2001; 2010 | 319 | Canada, Japan, South Africa, South Korea, Spain, Switzerland, UK, US |
| Educational level (%) | 2000 | 272 | Canada, Japan, South Korea, Spain, UK, US |
| PM_2.5_ (μg/m^3^) | 2014 | 334 | Brazil, Canada, Japan, South Africa, South Korea, Spain, Switzerland, Taiwan, UK, US |
| NO_2_ (ppb) | 1999-2001 | 331 | Brazil, Canada, Japan, South Africa, South Korea, Spain, Switzerland, Taiwan, UK, US |
| Average temperature (℃) | community-specific observational period of suicide data | 354 | Brazil, Canada, Japan, Mexico, Romania, South Africa, South Korea, Spain, Switzerland, Taiwan, UK, US |

**eTable 3.** Country-specific summary of the seasonality of suicide

| **Country** | **Week of**  **1^st^ Peak** | **1^st^ Peak/Trough RR**  **(95% CI)** | **Week of**  **2^nd^ Peak** | **2^nd^ Peak/Trough RR**  **(95% CI)** | **Week of Trough** |
| --- | --- | --- | --- | --- | --- |
| Brazil | 11 | 1.20 (1.08-1.33) | 40 | 1.17 (1.05-1.31) | 1 |
| Canada | 17 | 1.23 (1.18-1.28) | - | - | 1 |
| Japan | 16 | 1.29 (1.26-1.31) | 42 | 1.13 (1.11-1.16) | 1 |
| Mexico | 21 | 1.24 (1.16, 1.32) | 41 | 1.16 (1.08, 1.23) | 2 |
| Romania | 29 | 1.47 (1.33, 1.62) | - | - | 52 |
| South Africa | 1 | 1.37 (1.24-1.51) | - | - | 20 |
| South Korea | 17 | 1.45 (1.38-1.54) | 39 | 1.32 (1.25-1.40) | 1 |
| Spain | 18 | 1.23 (1.17-1.29) | - | - | 52 |
| Switzerland | 16 | 1.12 (1.05-1.20) | 42 | 1.10 (1.02-1.18) | 1 |
| Taiwan | 18 | 1.21 (1.11-1.32) | 41 | 1.14 (1.05-1.25) | 1 |
| UK | 18 | 1.05 (1.01-1.10) | - | - | 51 |
| US | 17 | 1.15 (1.12-1.18) | - | - | 52 |

The table shows the weeks of a year in which the number of suicides is the highest, the second highest (week of peak & week of second peak) and the lowest (week of trough). RR, relative risk; CI, confidence interval; UK, United Kingdom; US, United States.

**eFigure 1.**


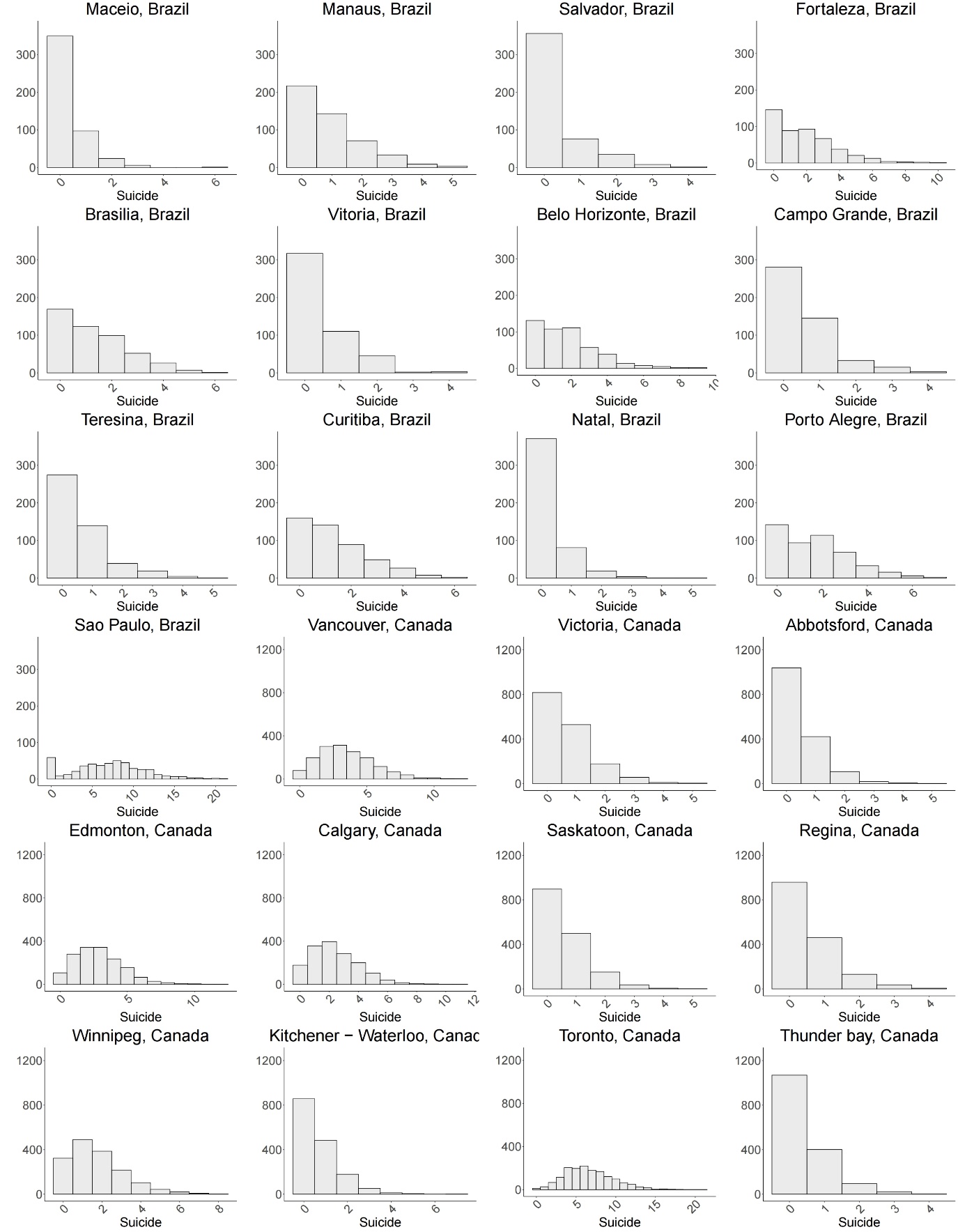

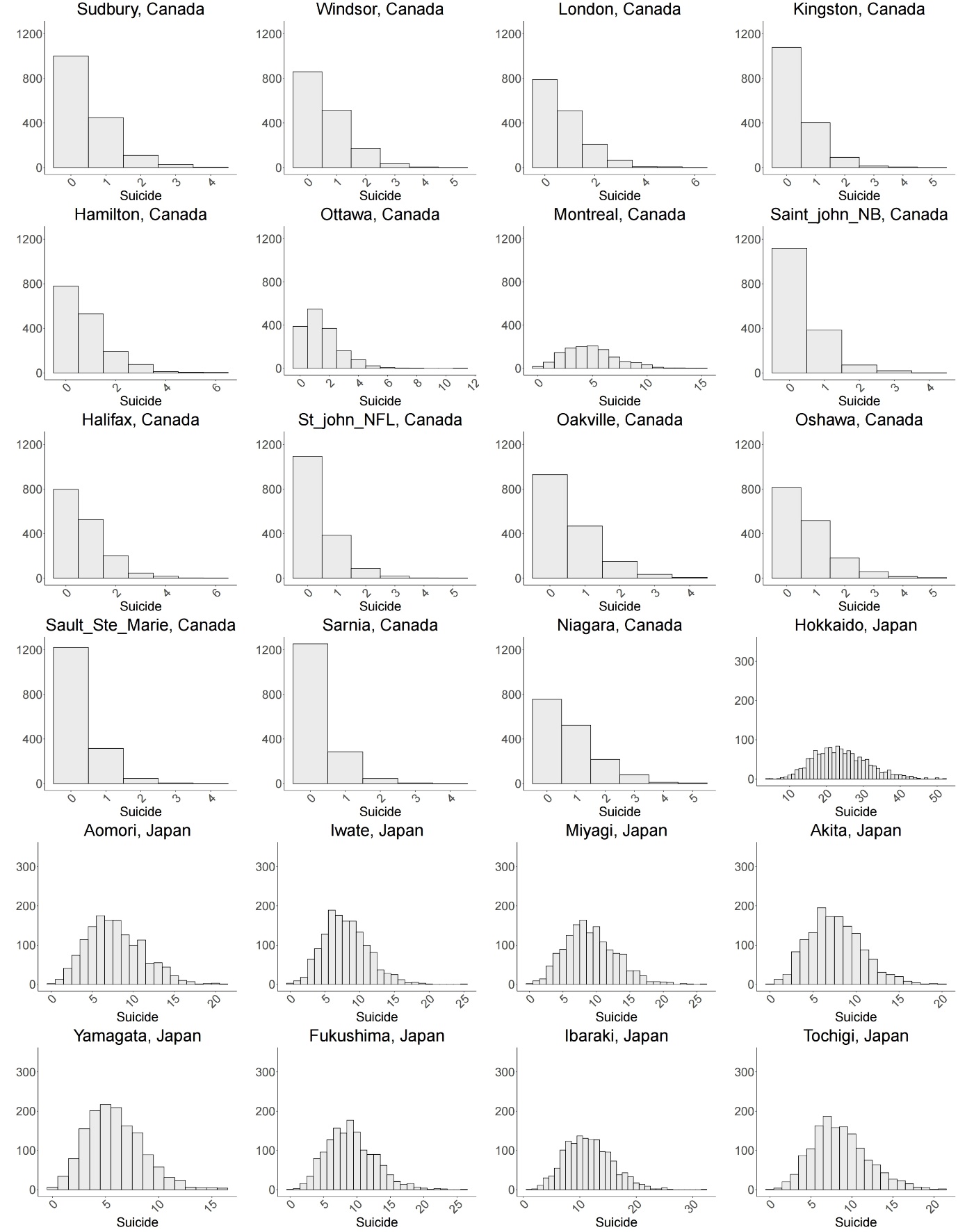

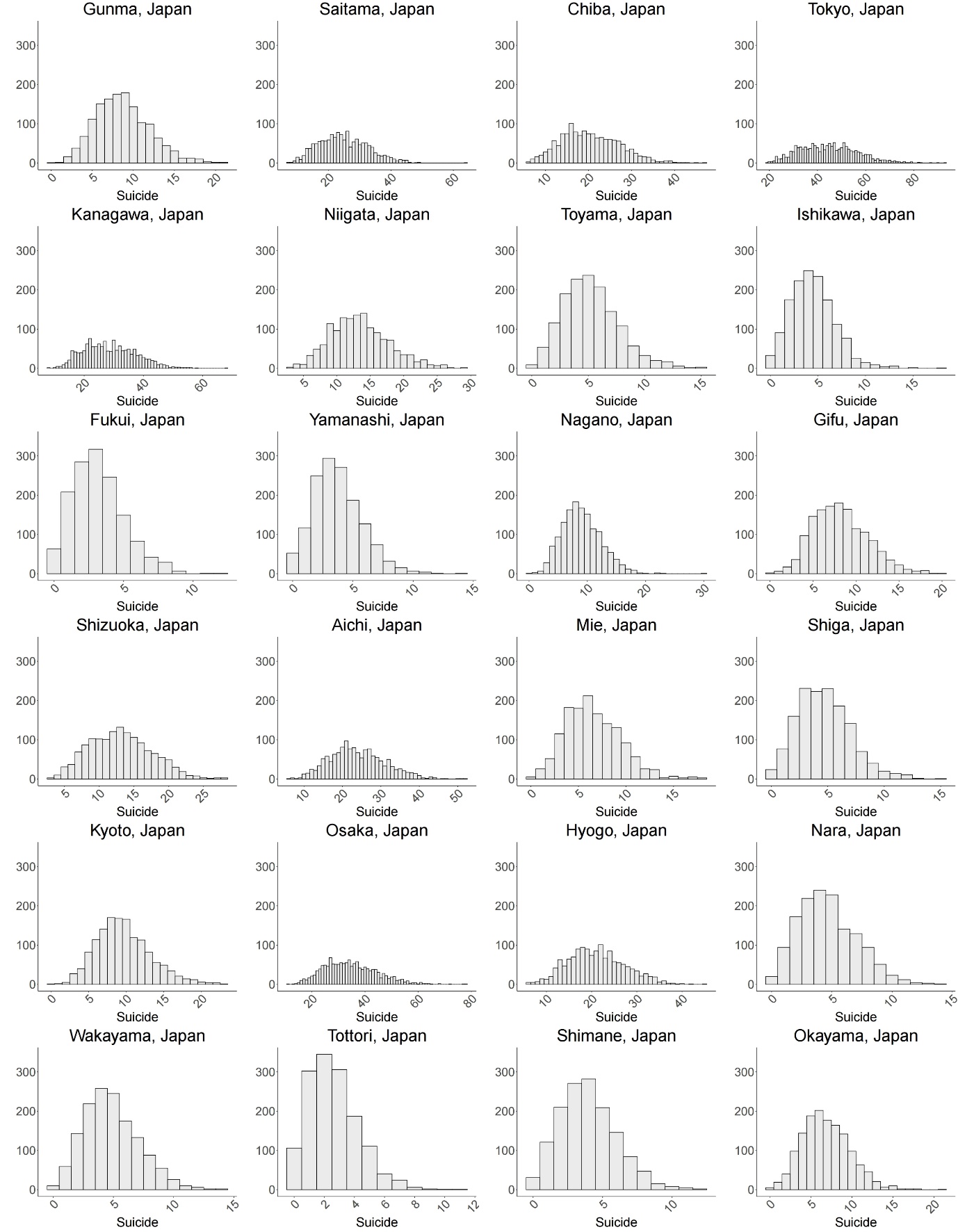

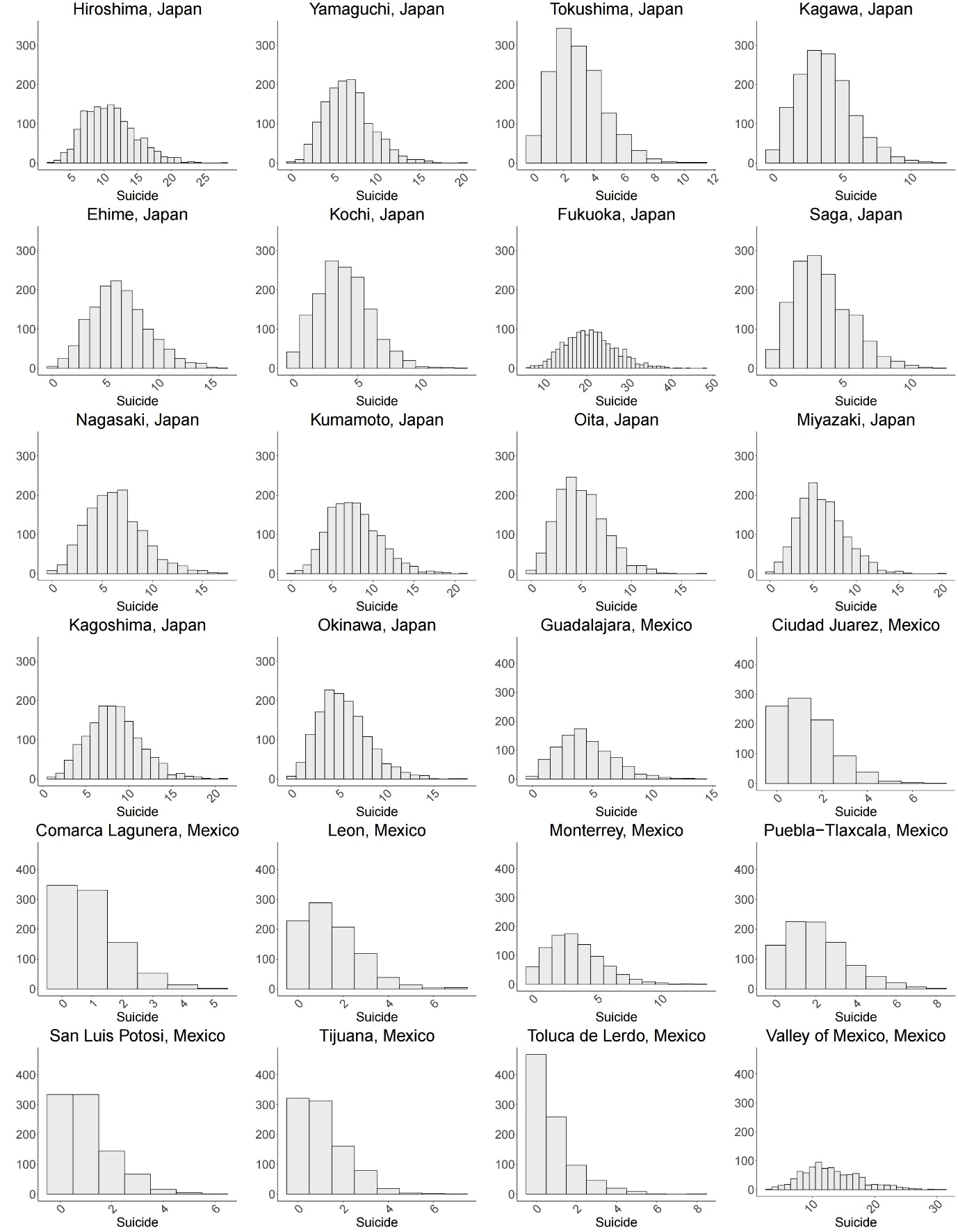

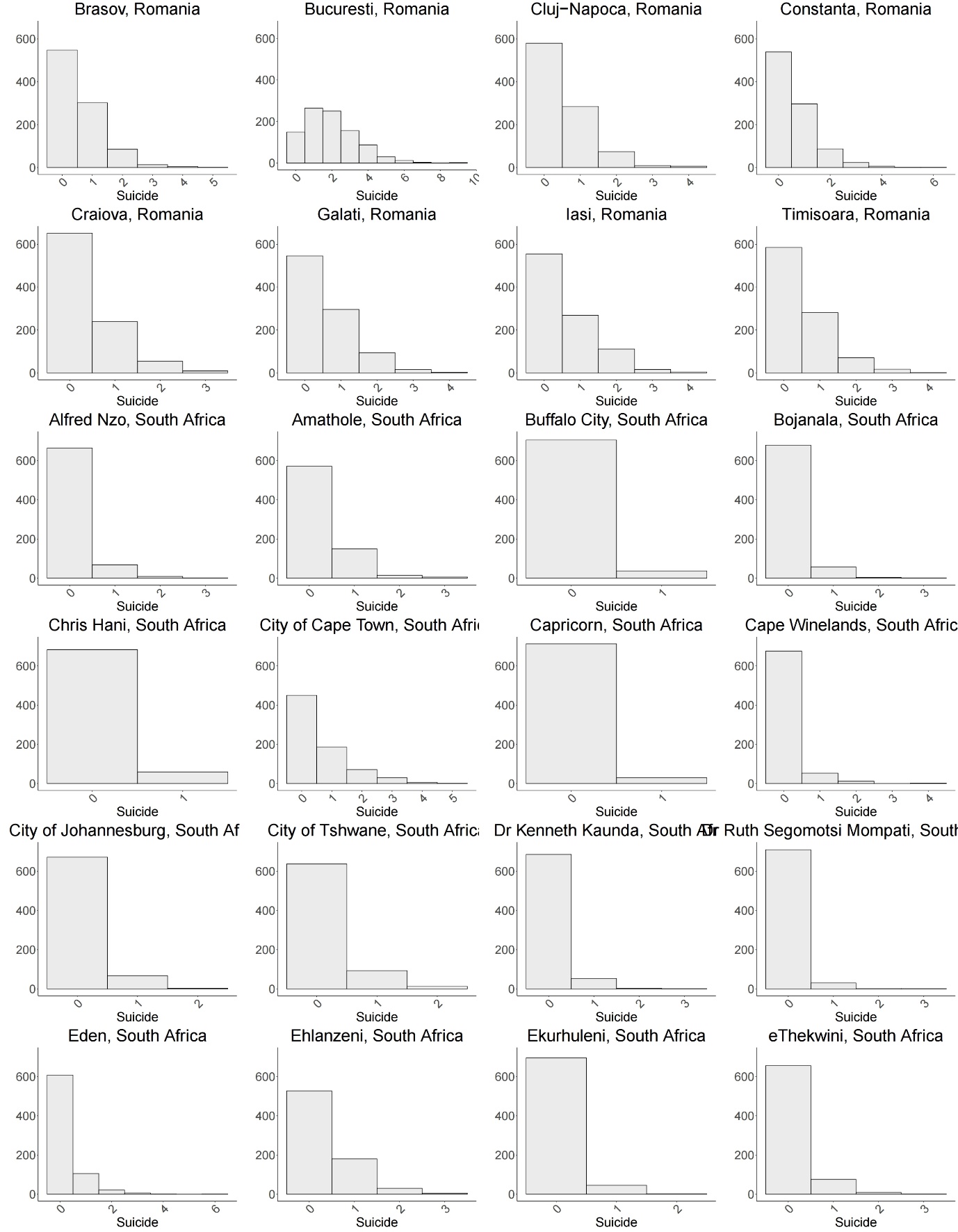

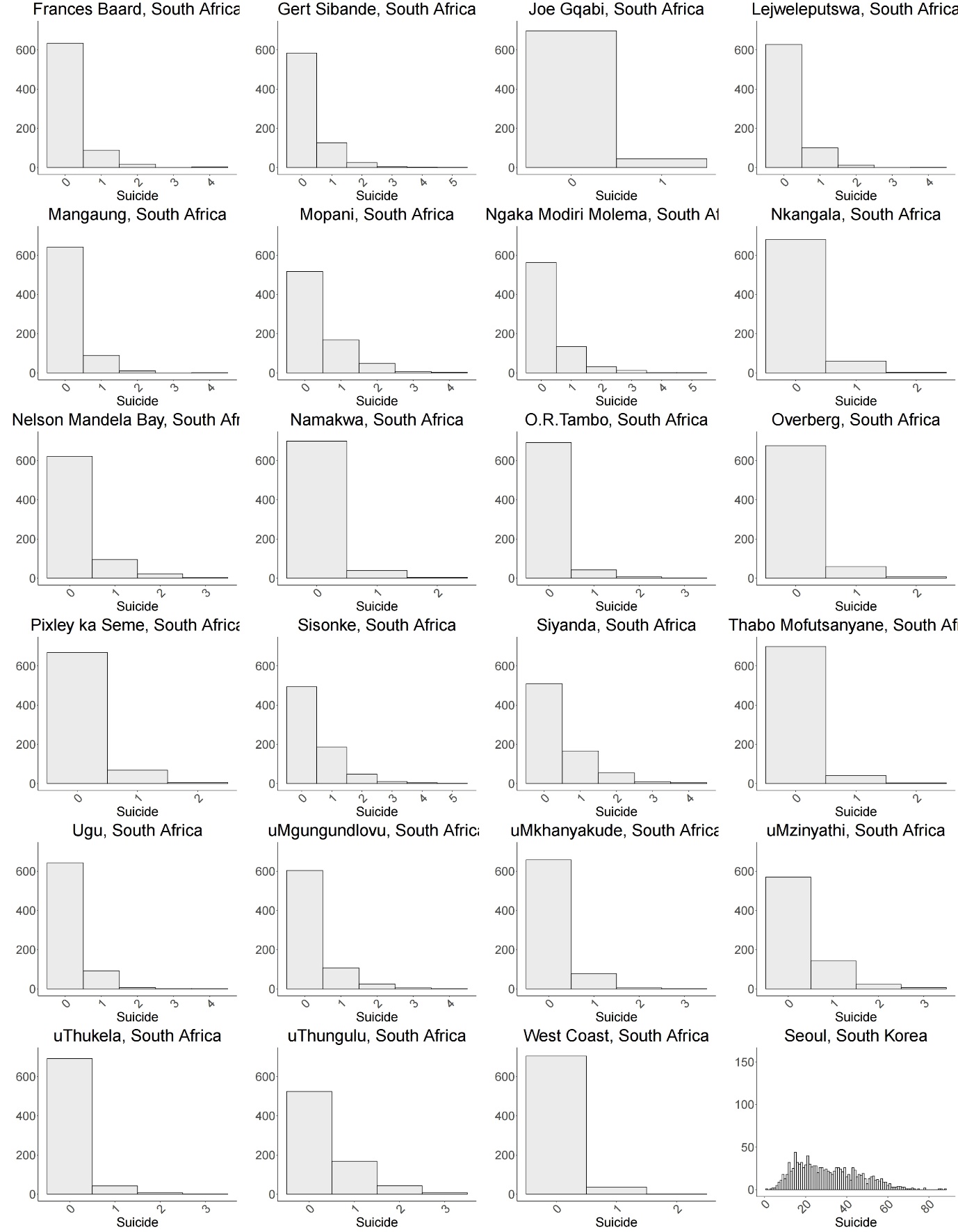

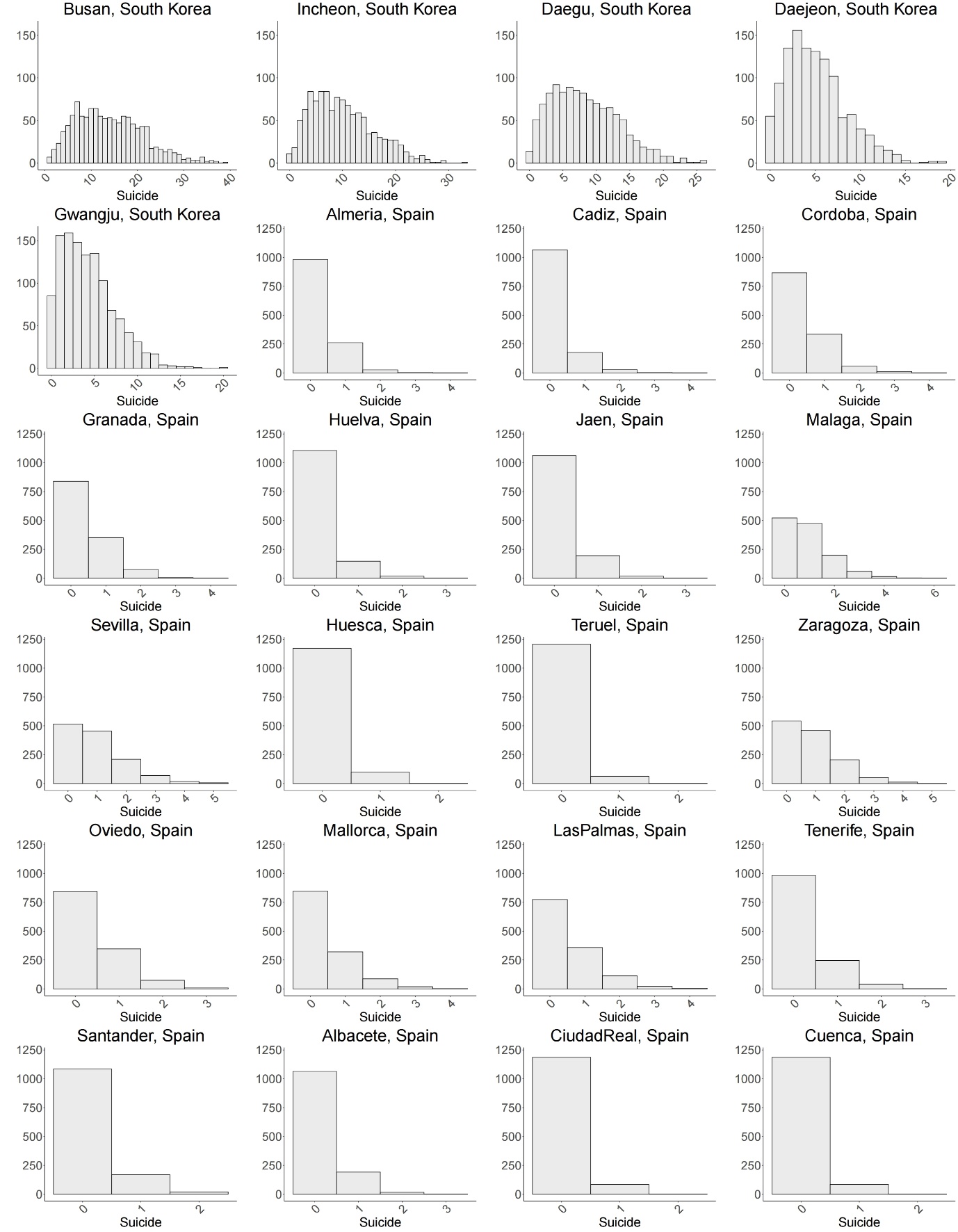

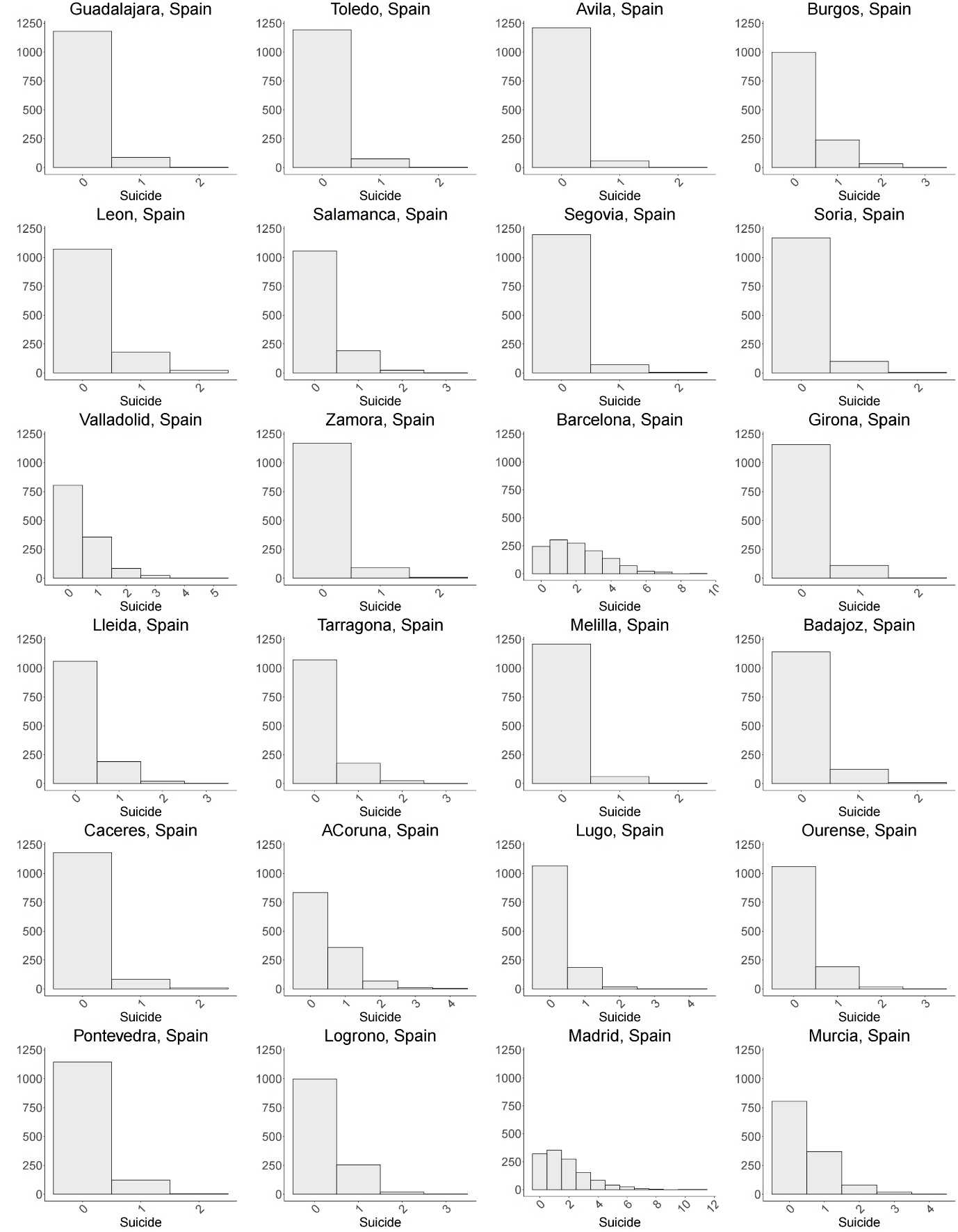

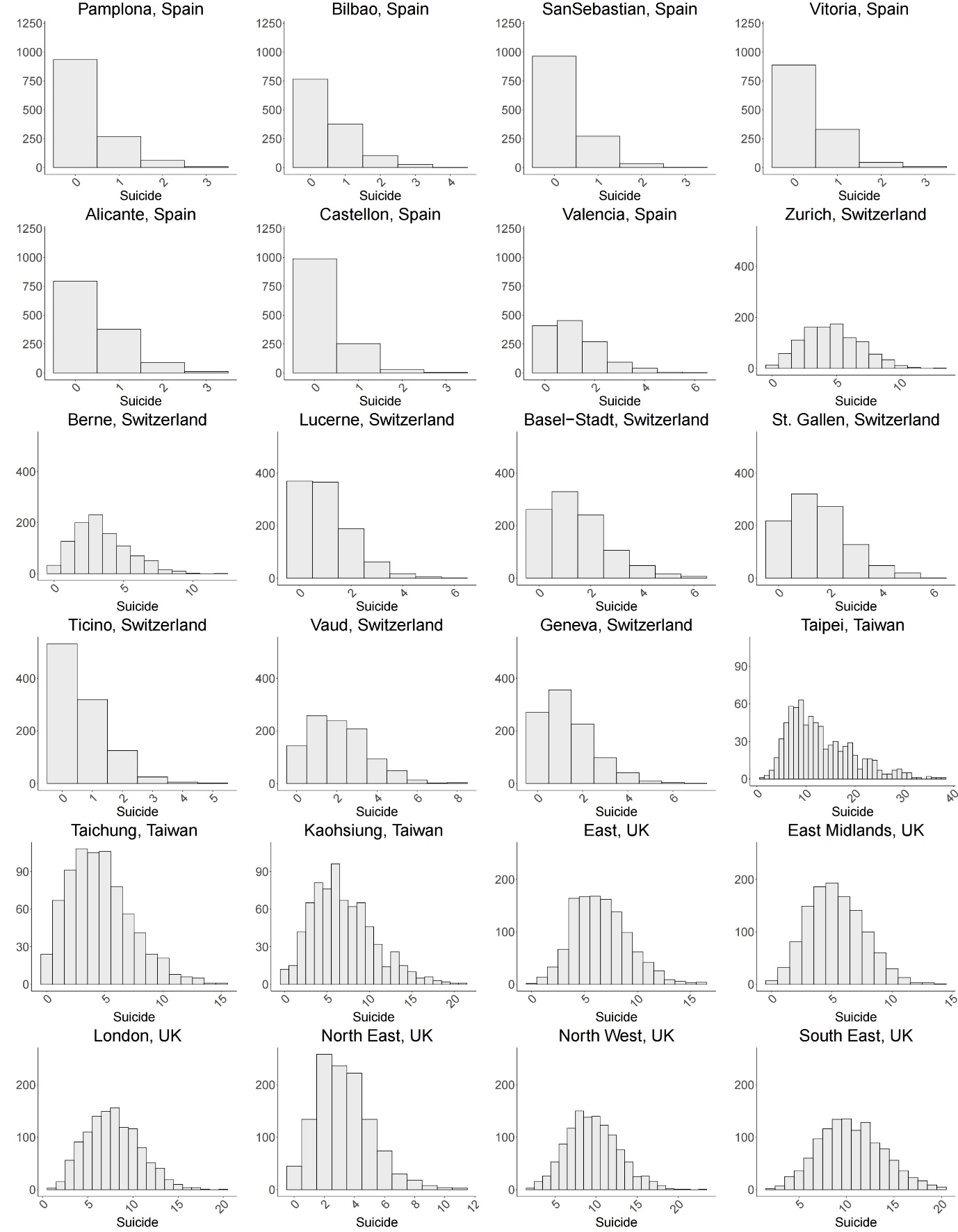

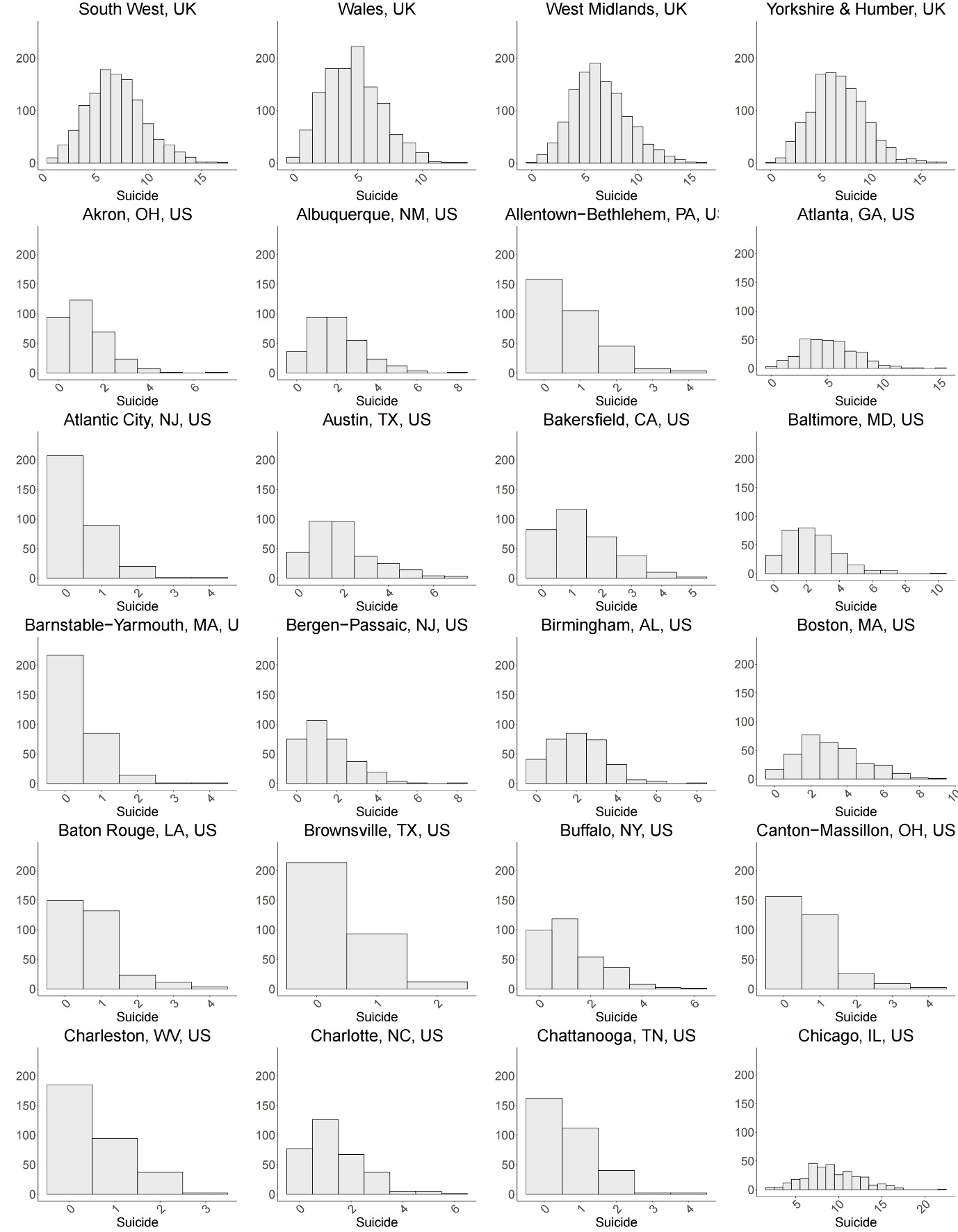

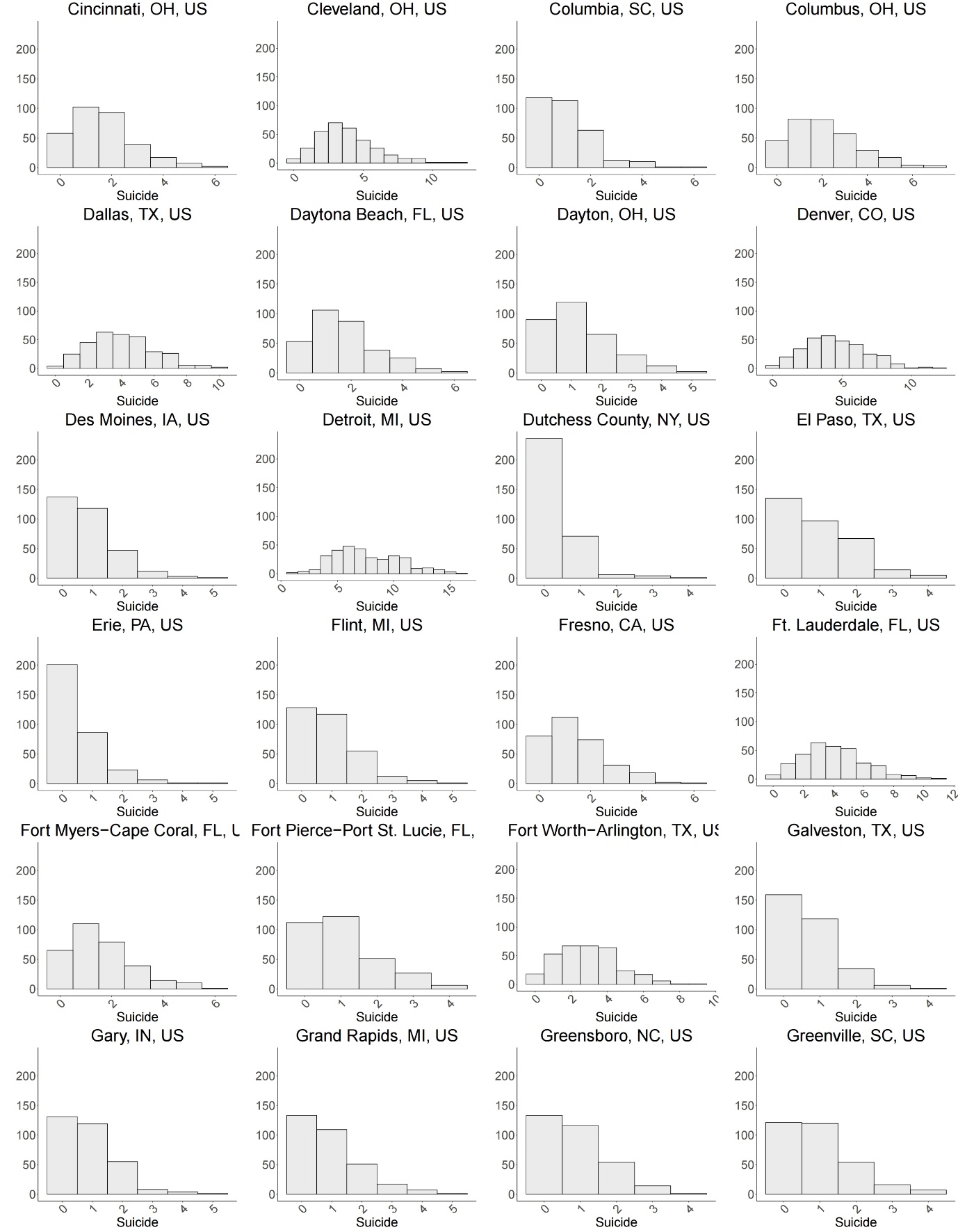

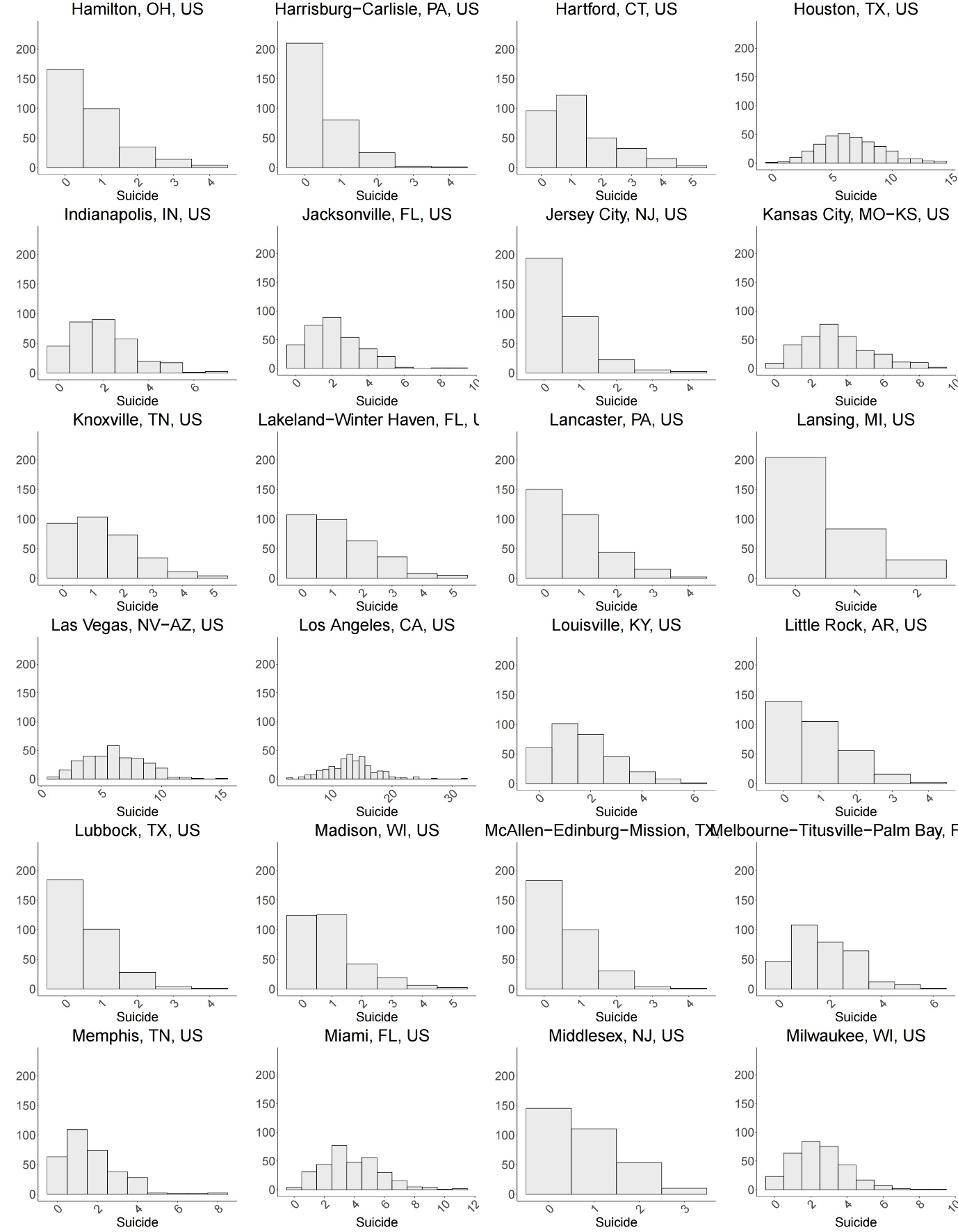

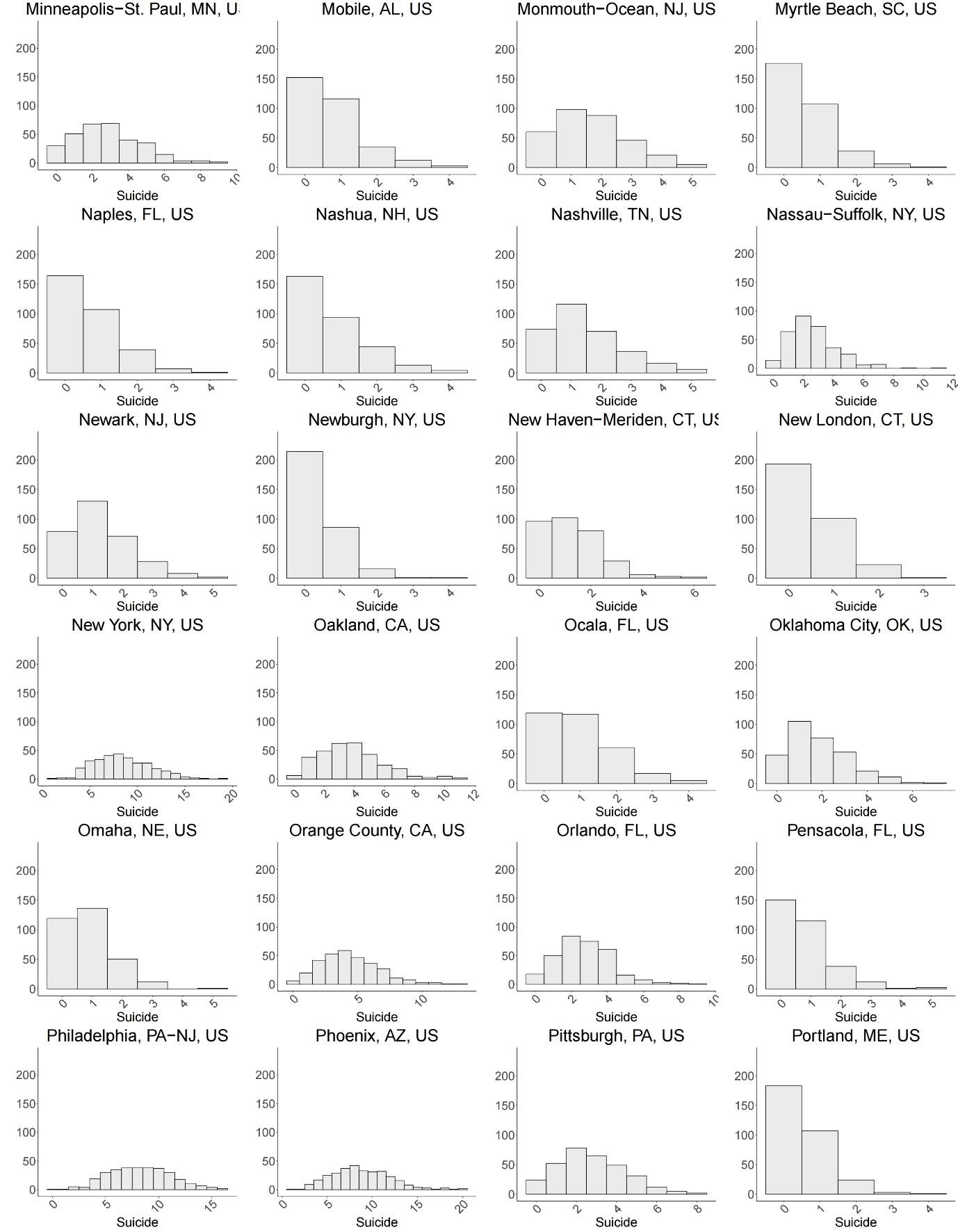

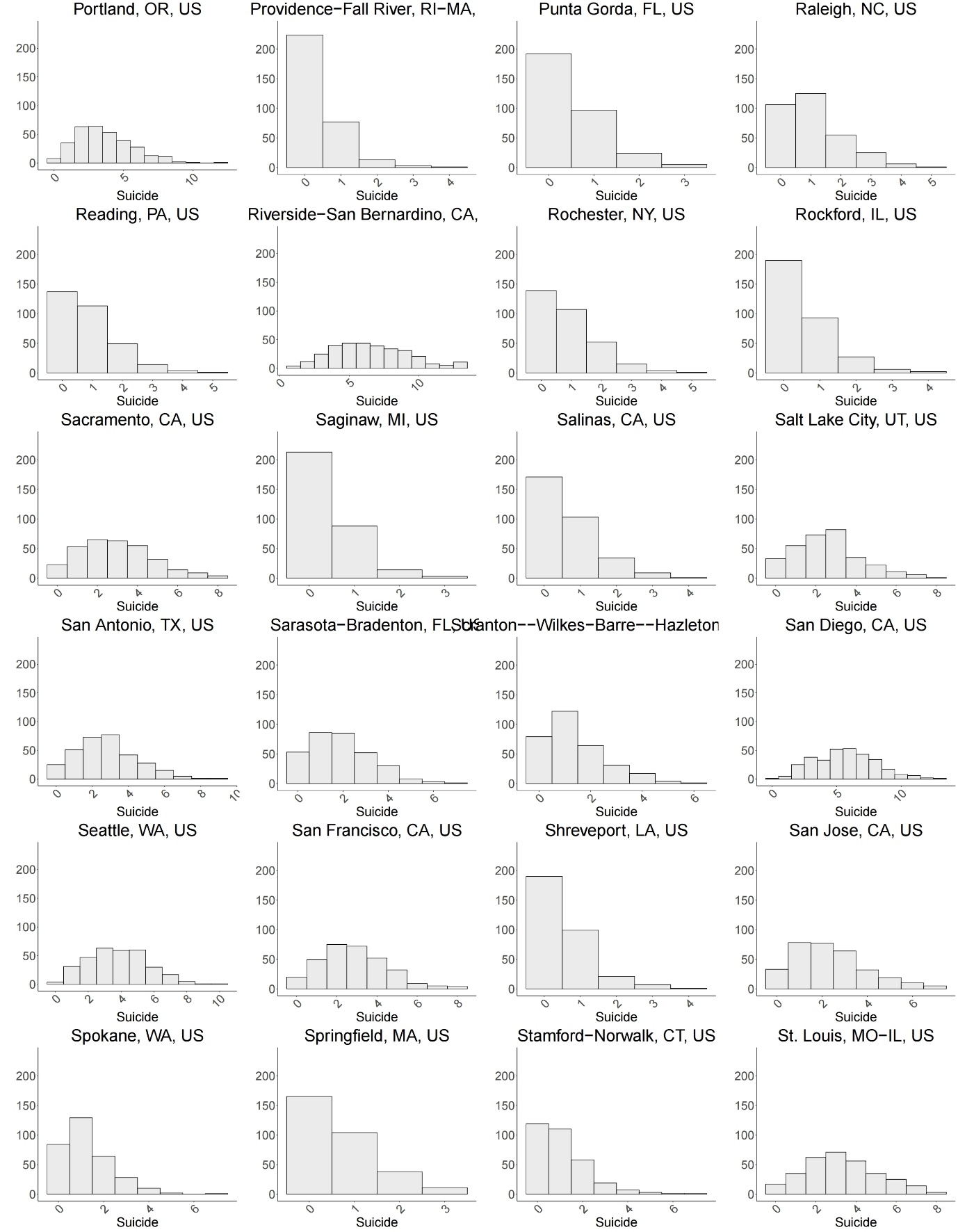

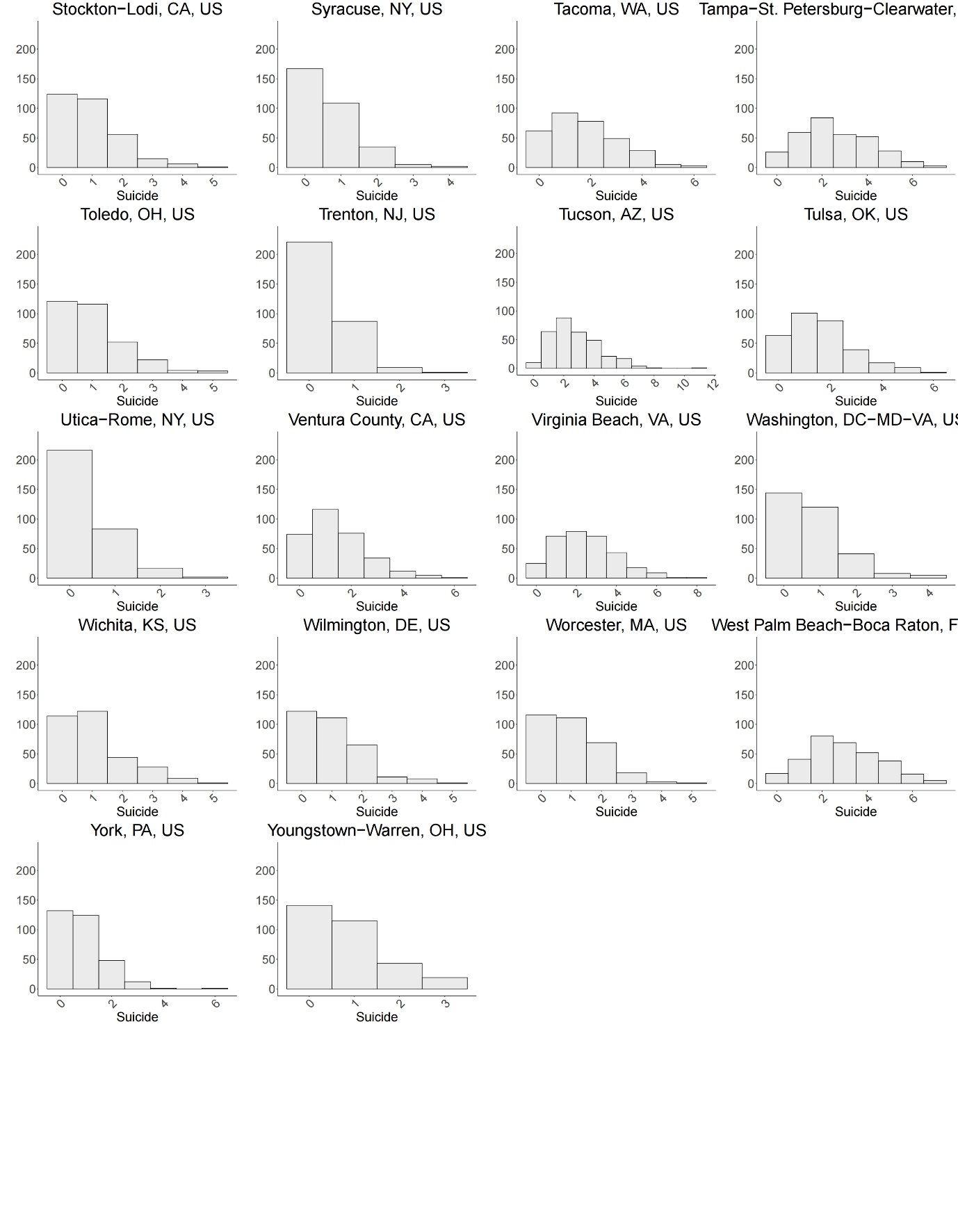


**eFigure 2.**


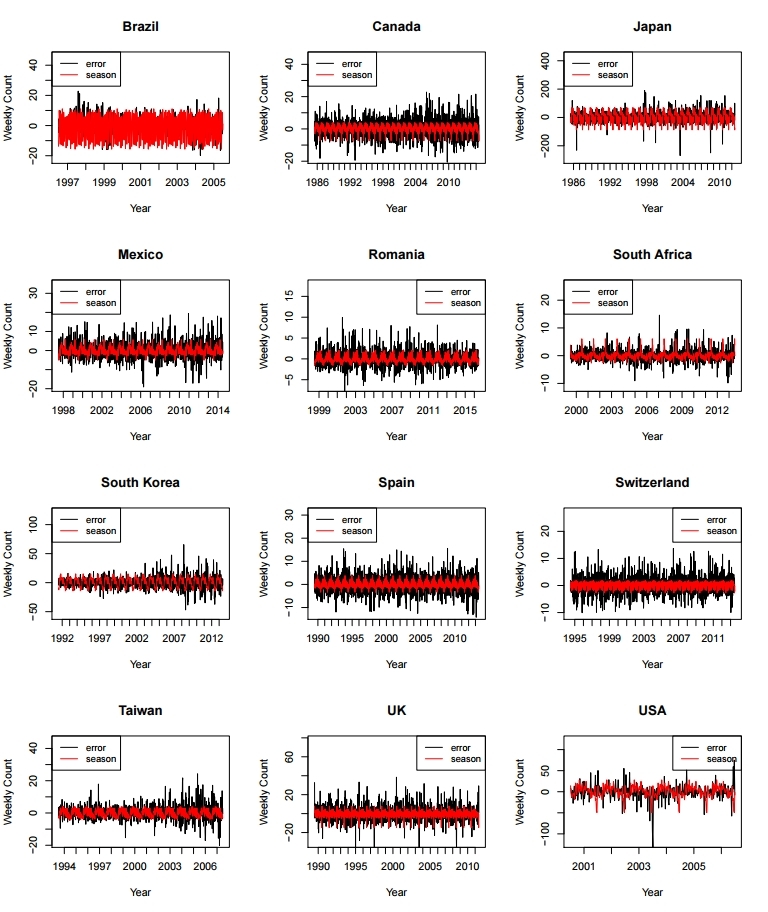


**eFigure 3.**


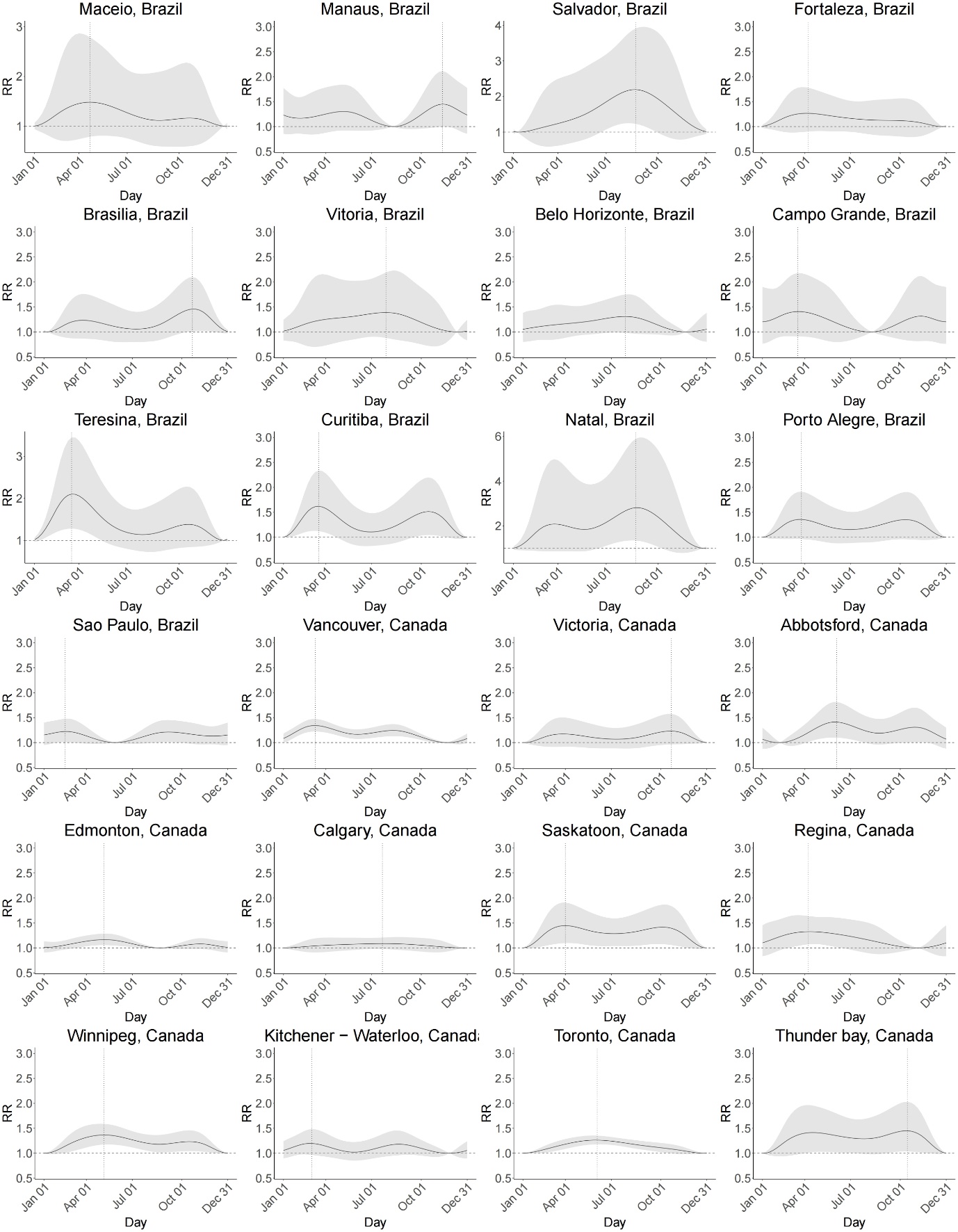

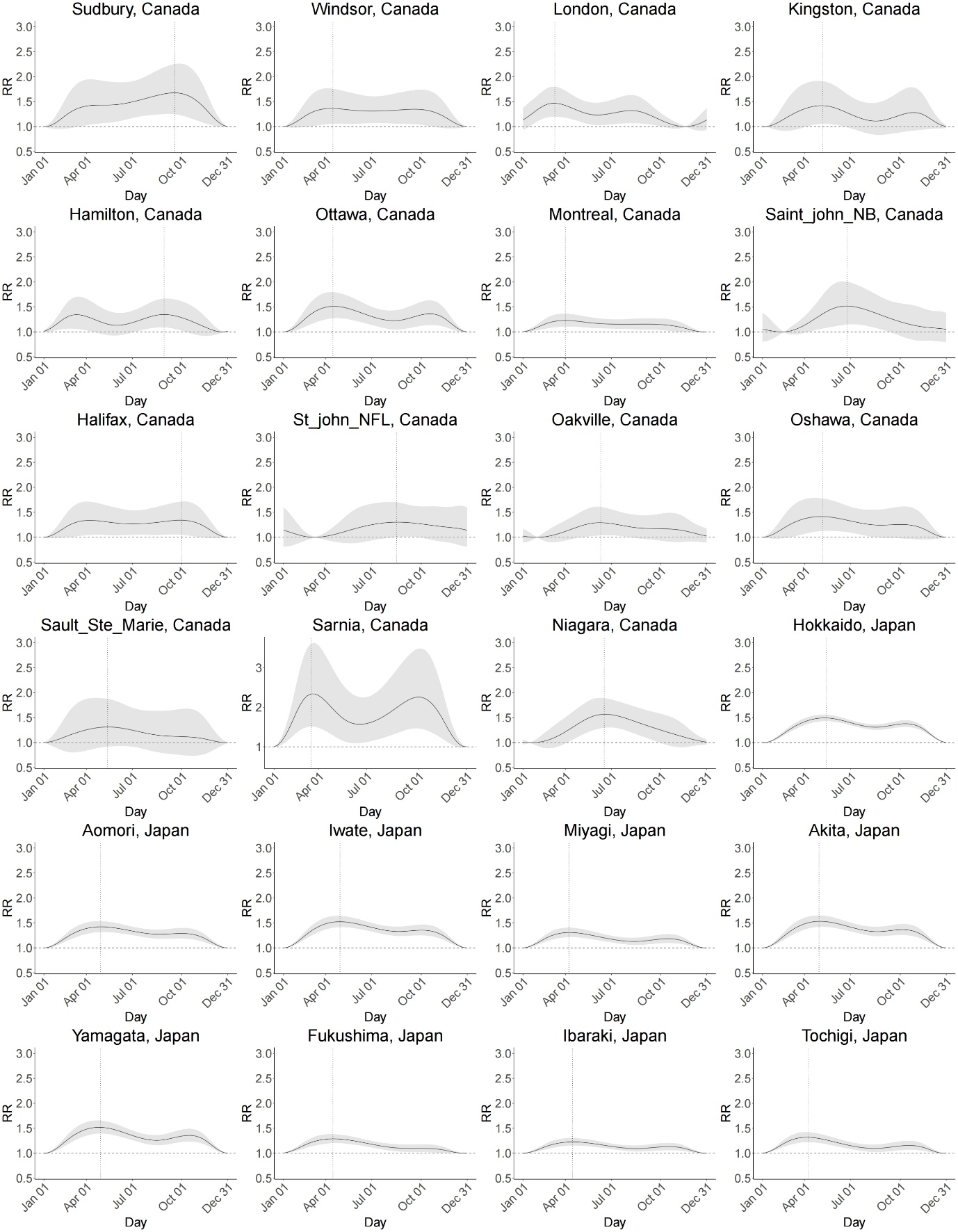

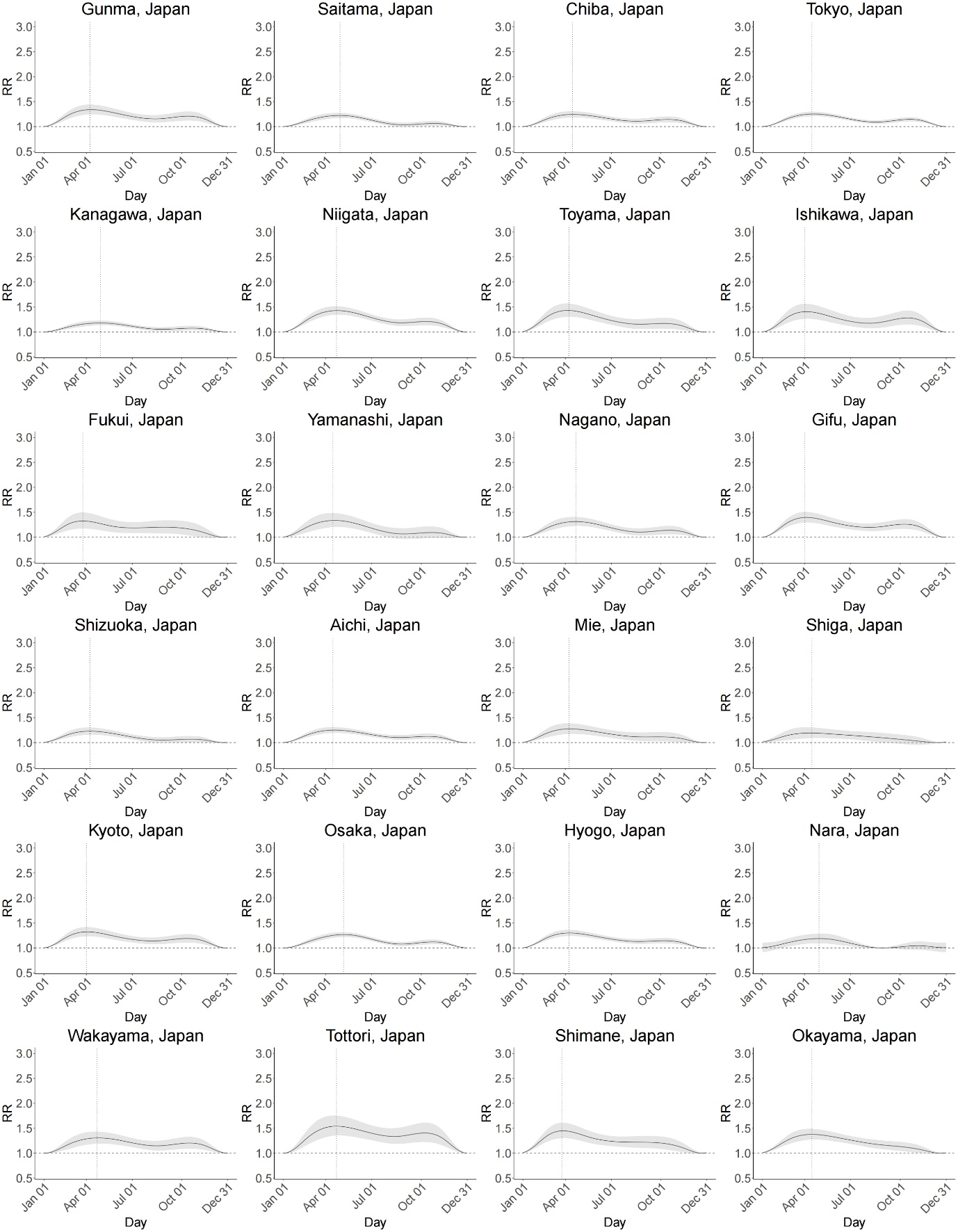

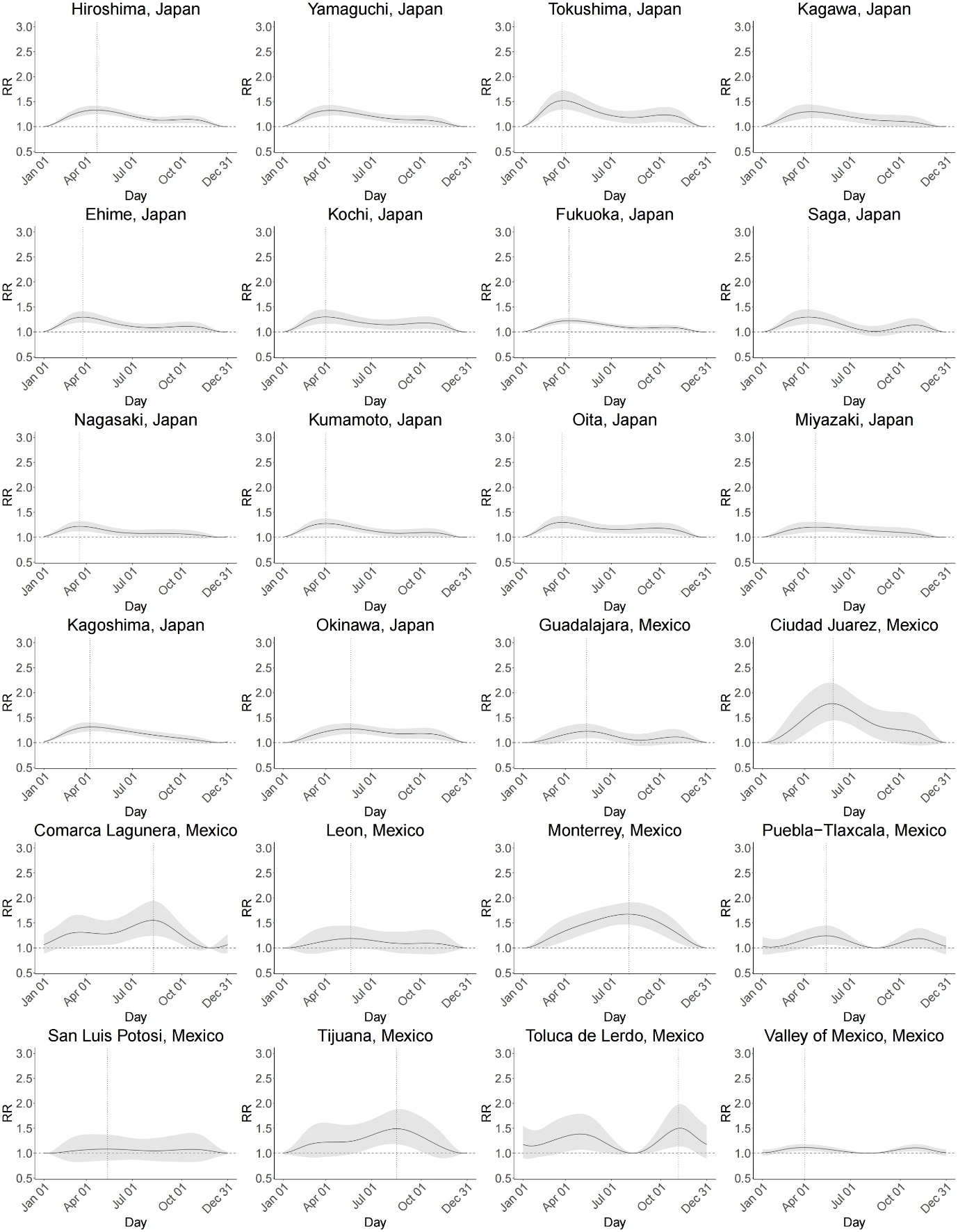

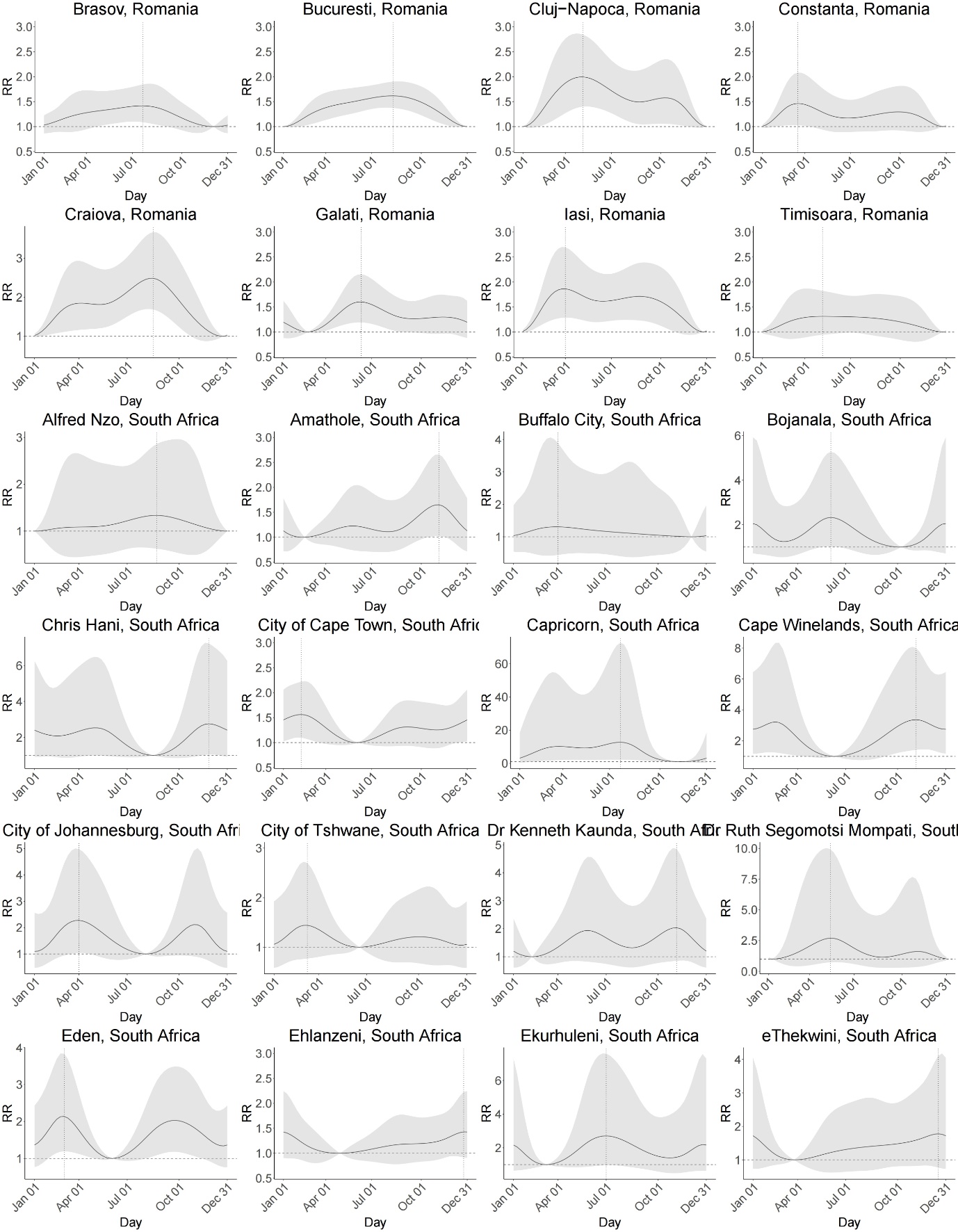

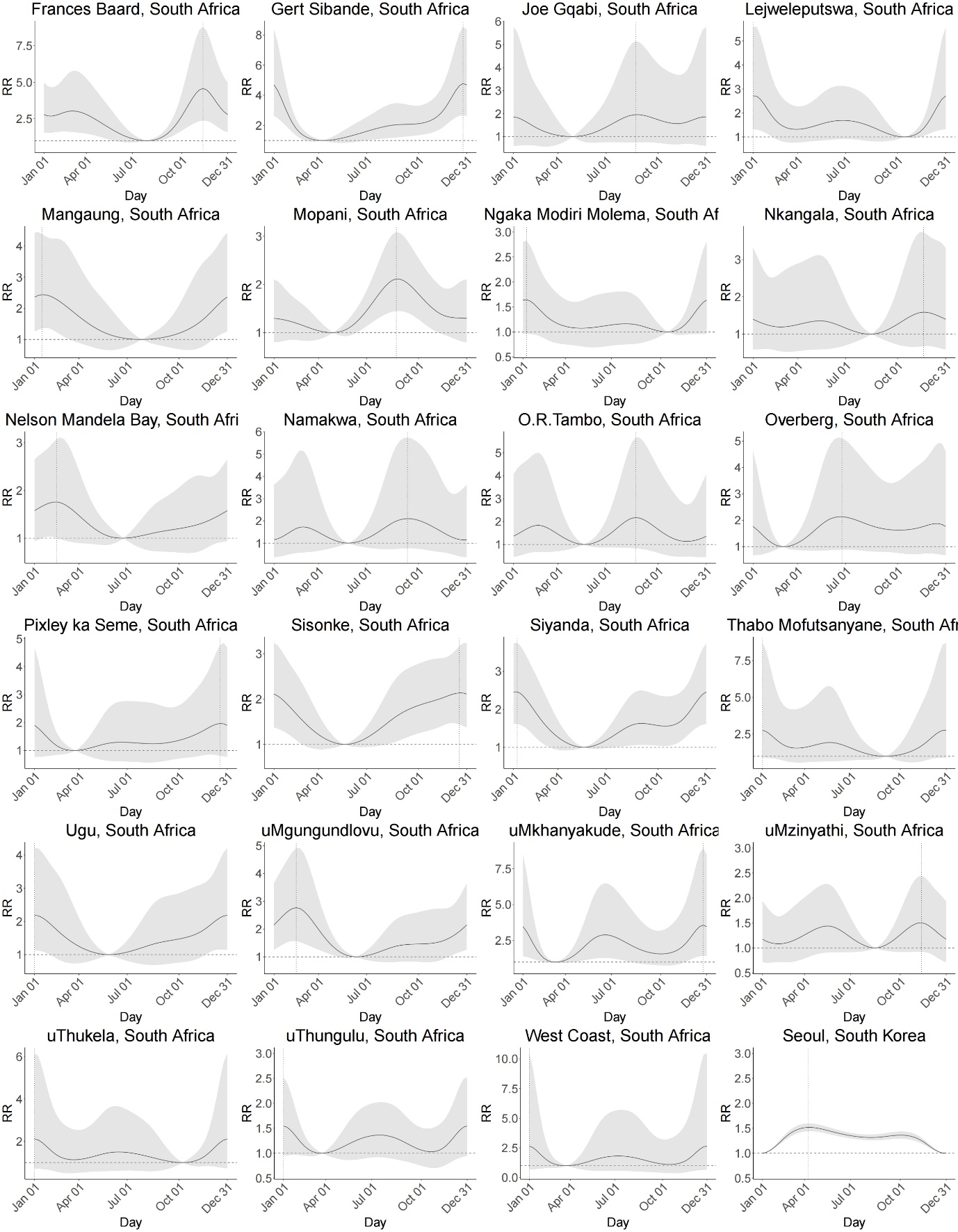

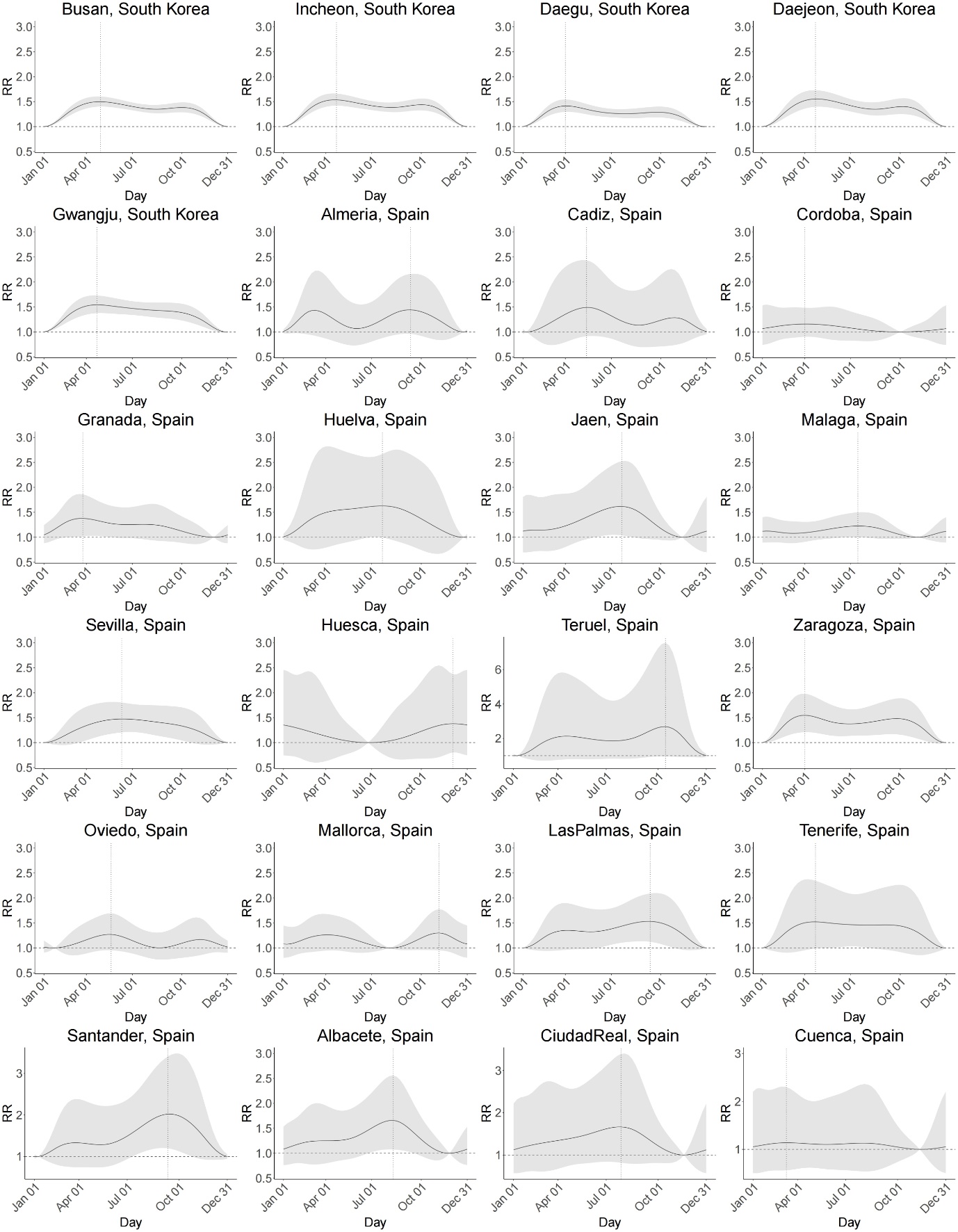

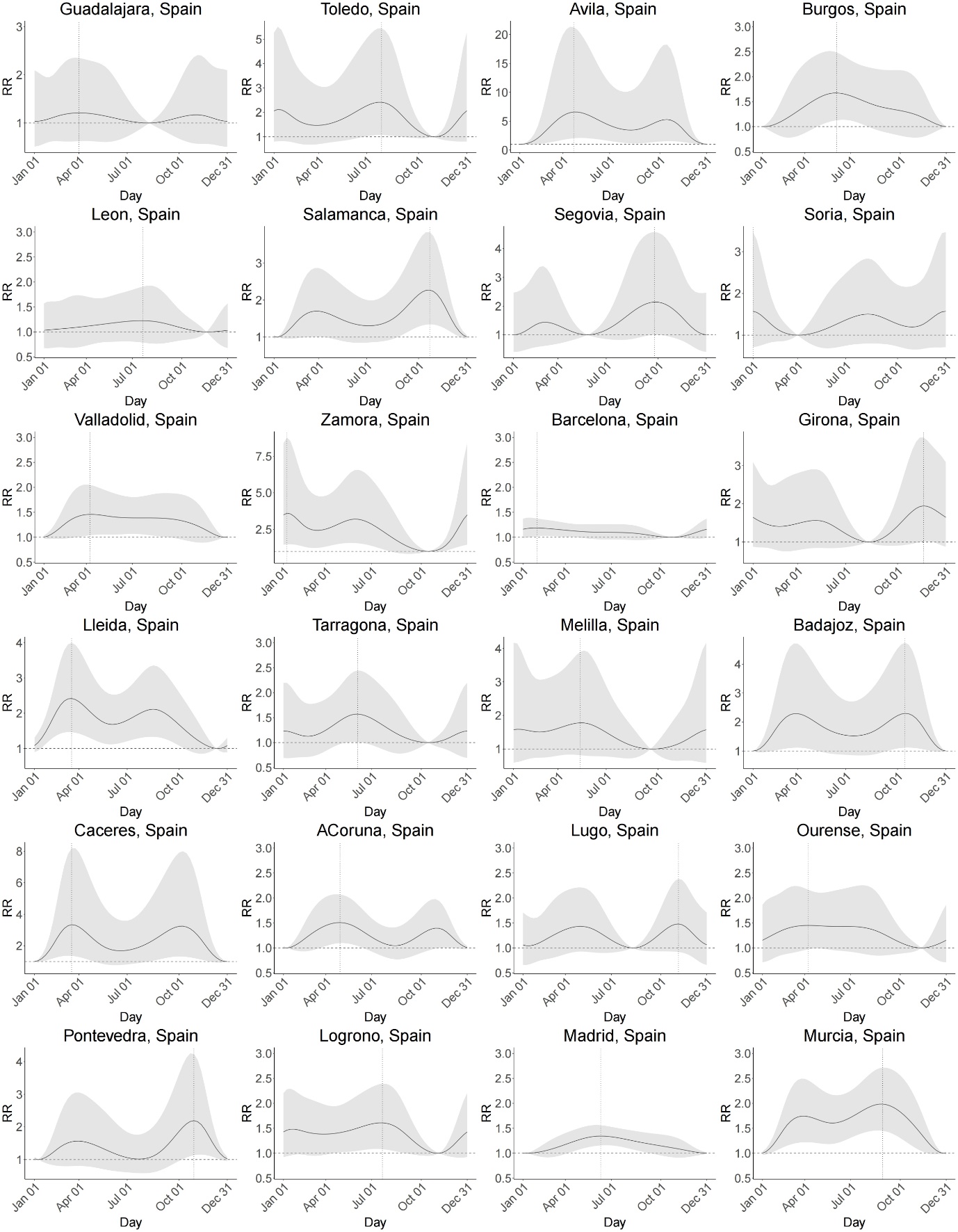

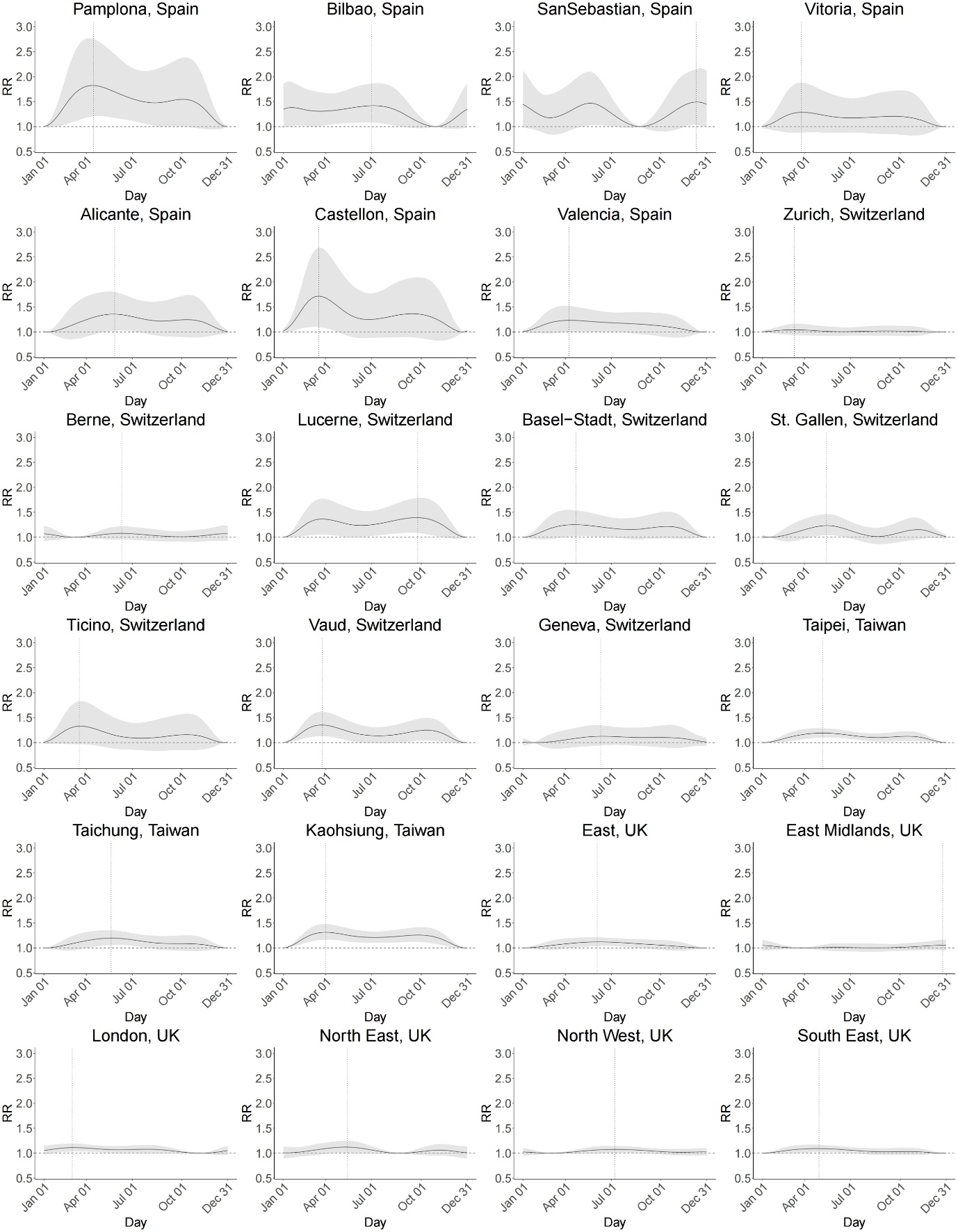

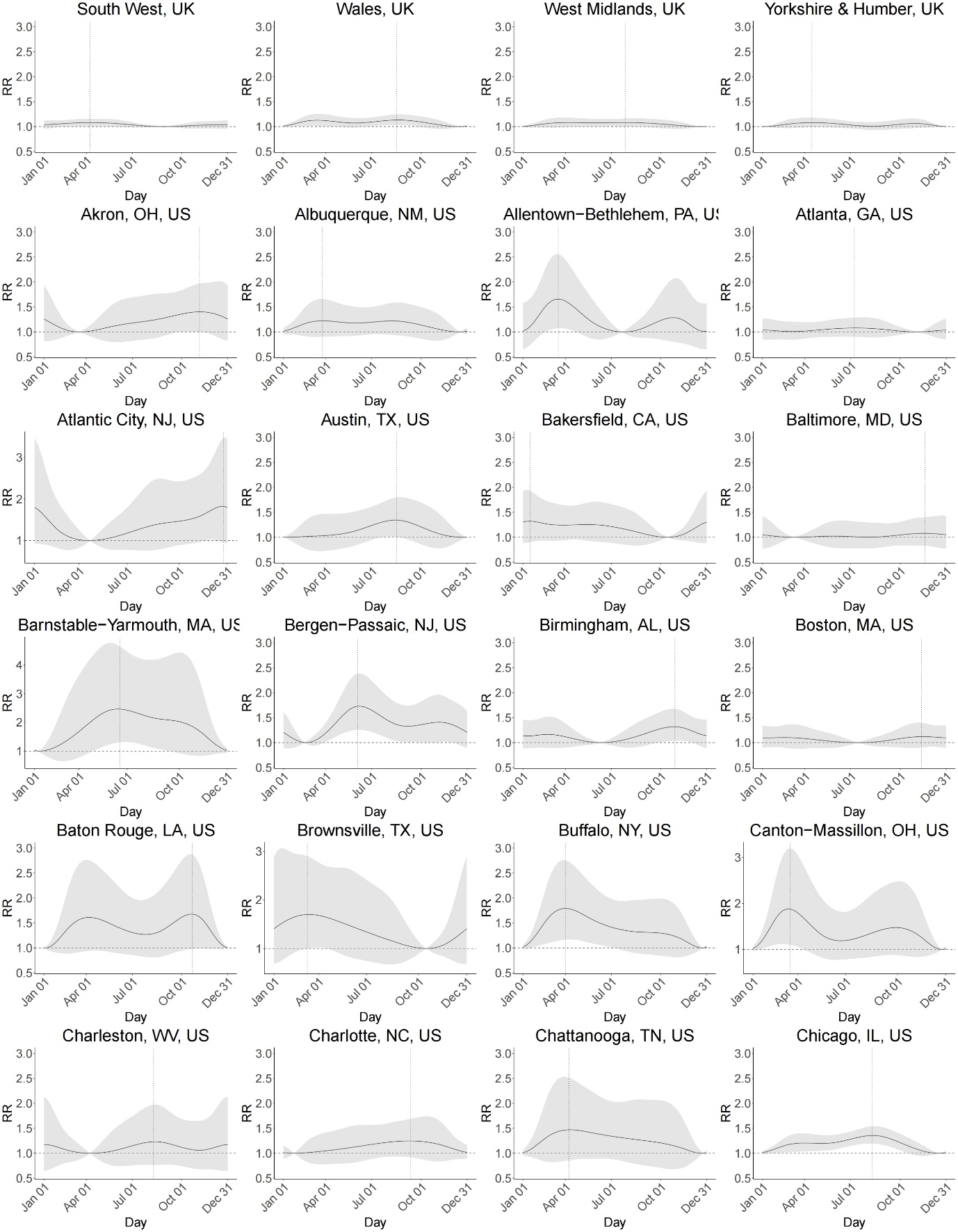

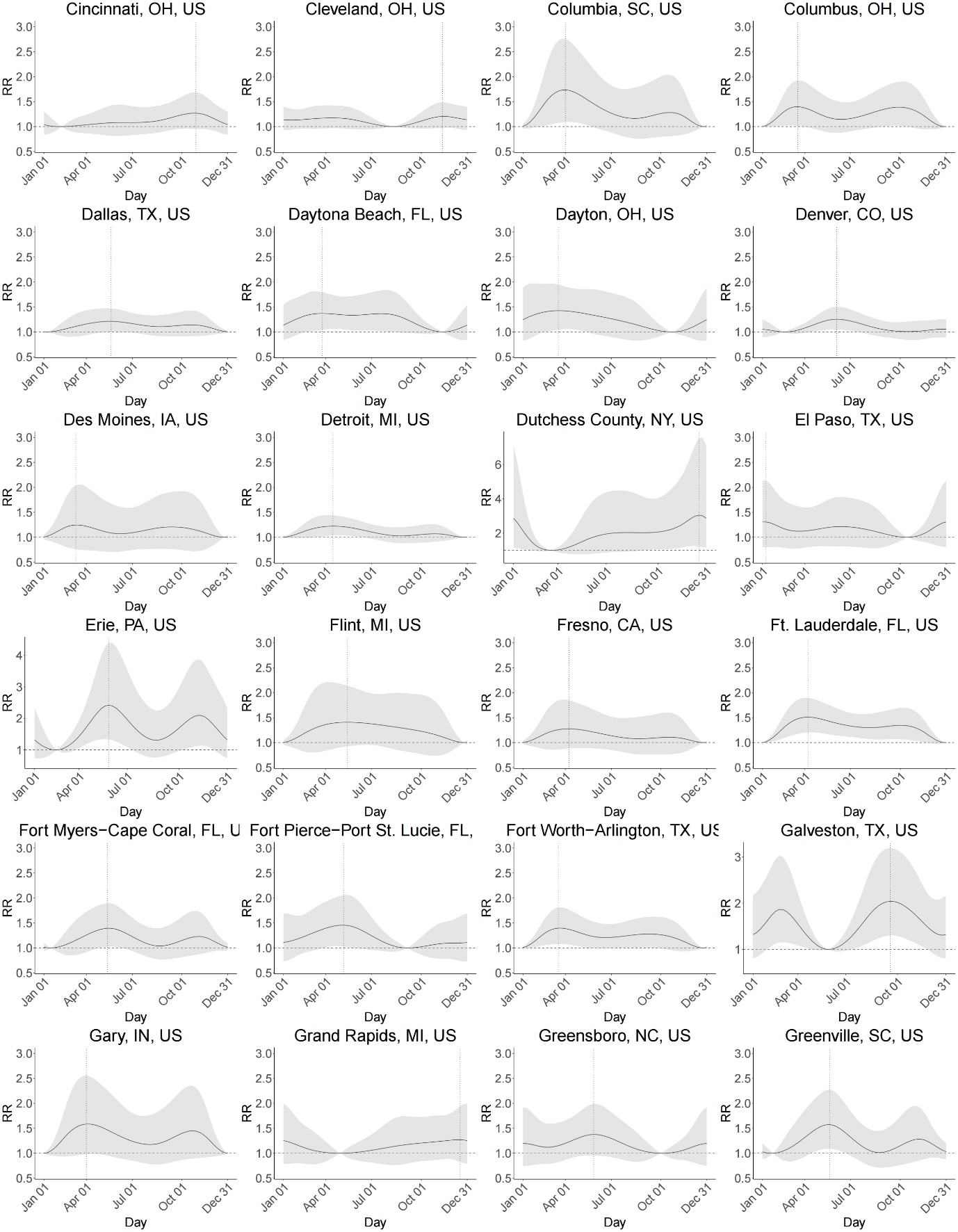

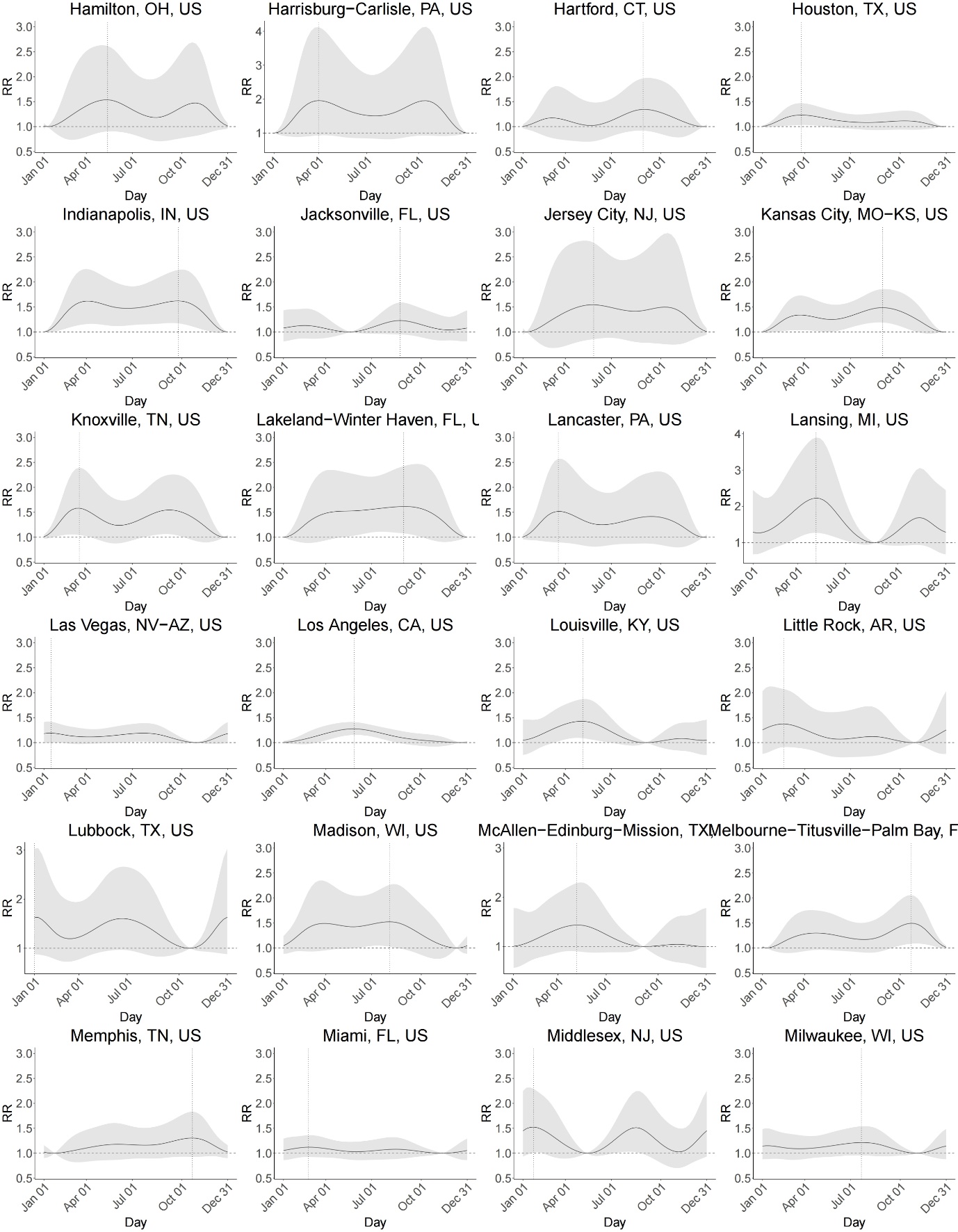

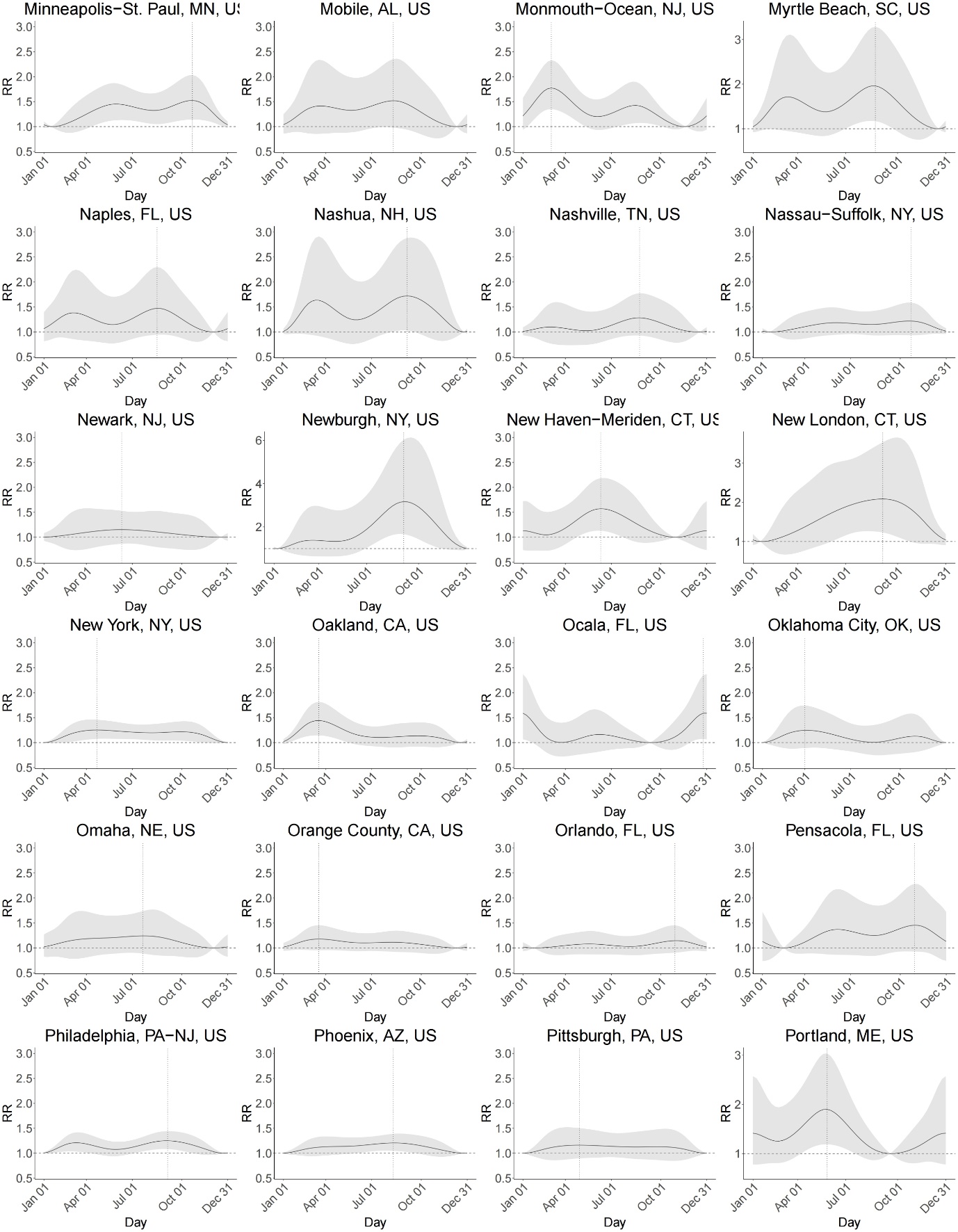

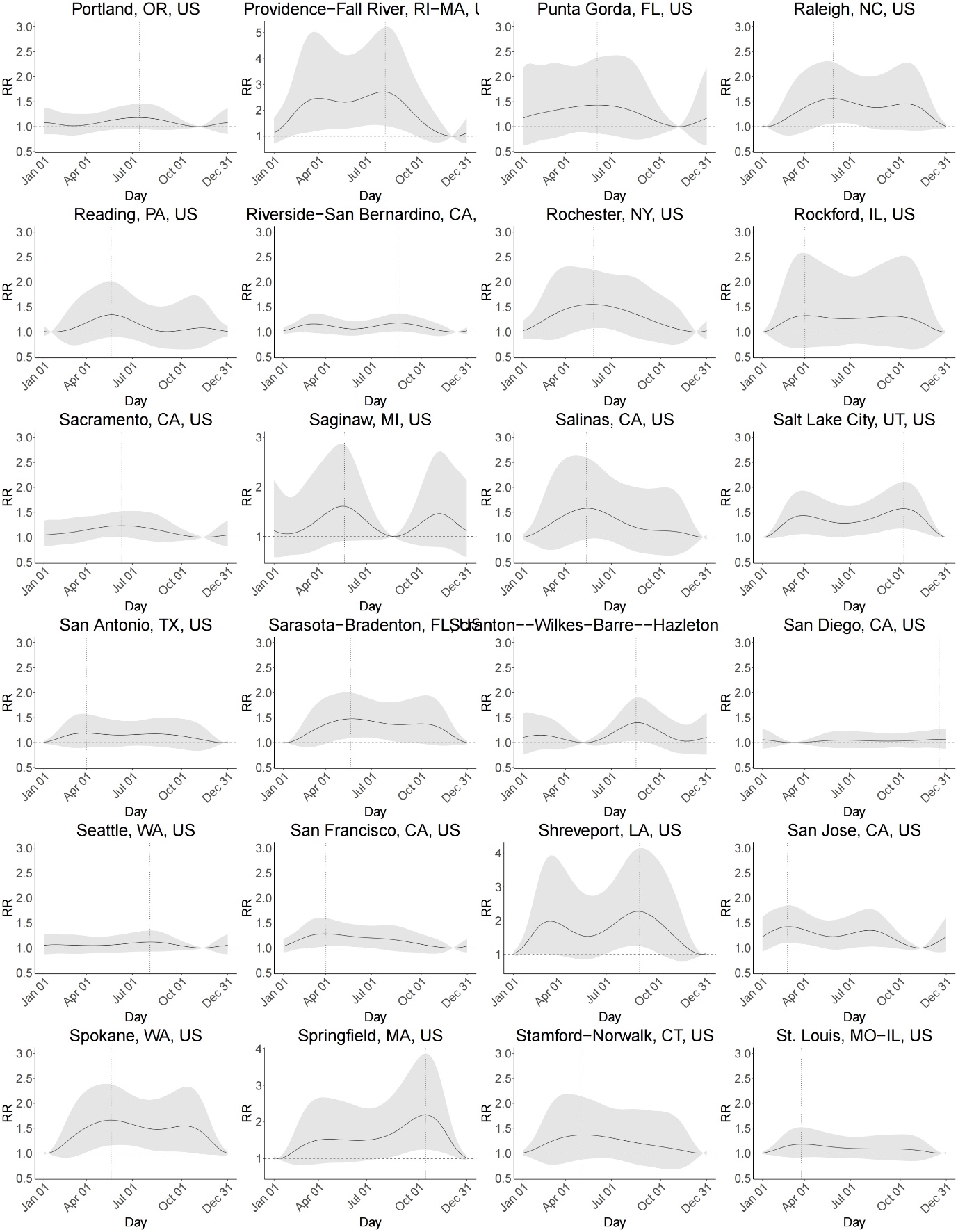

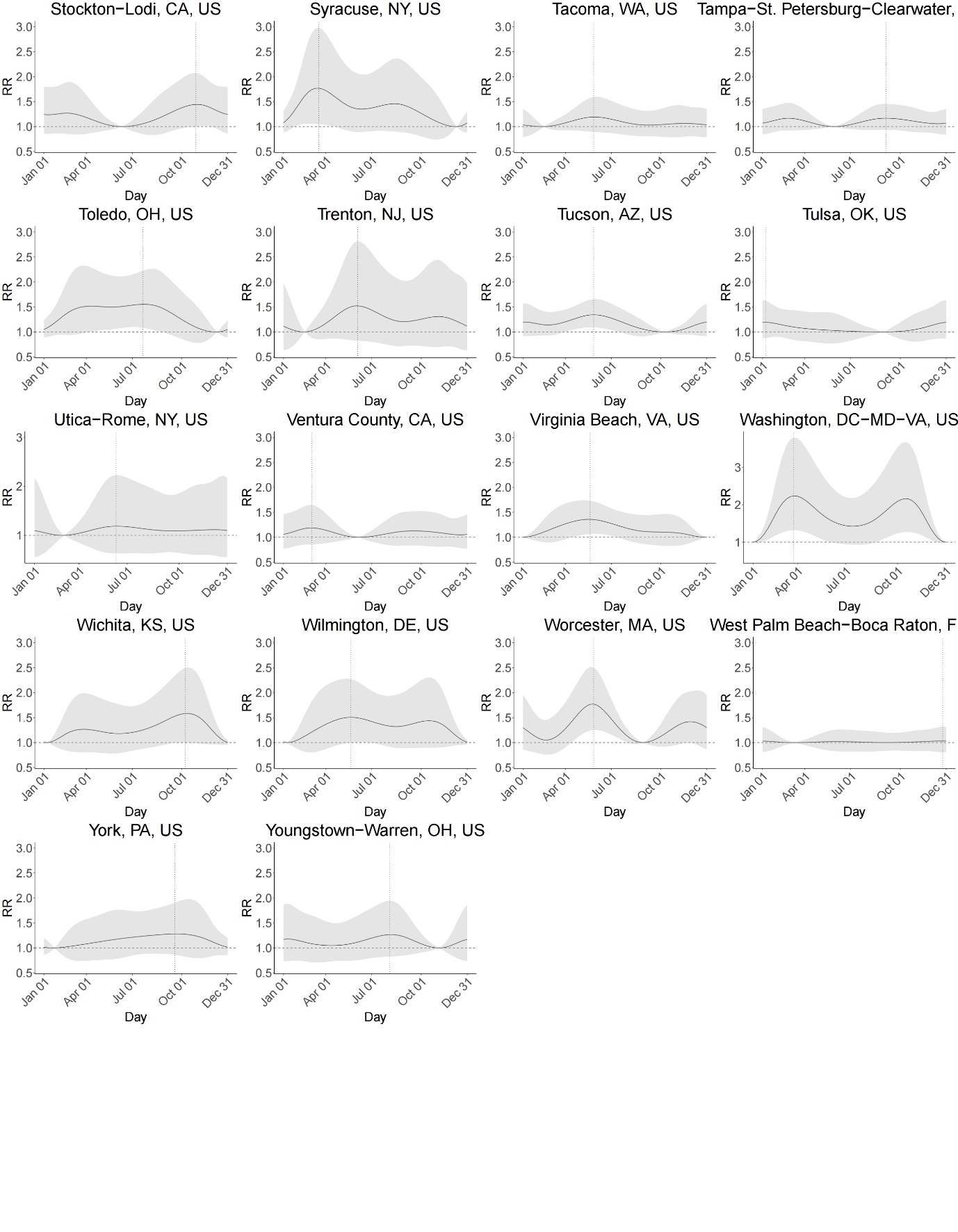


**eFigure 4.**


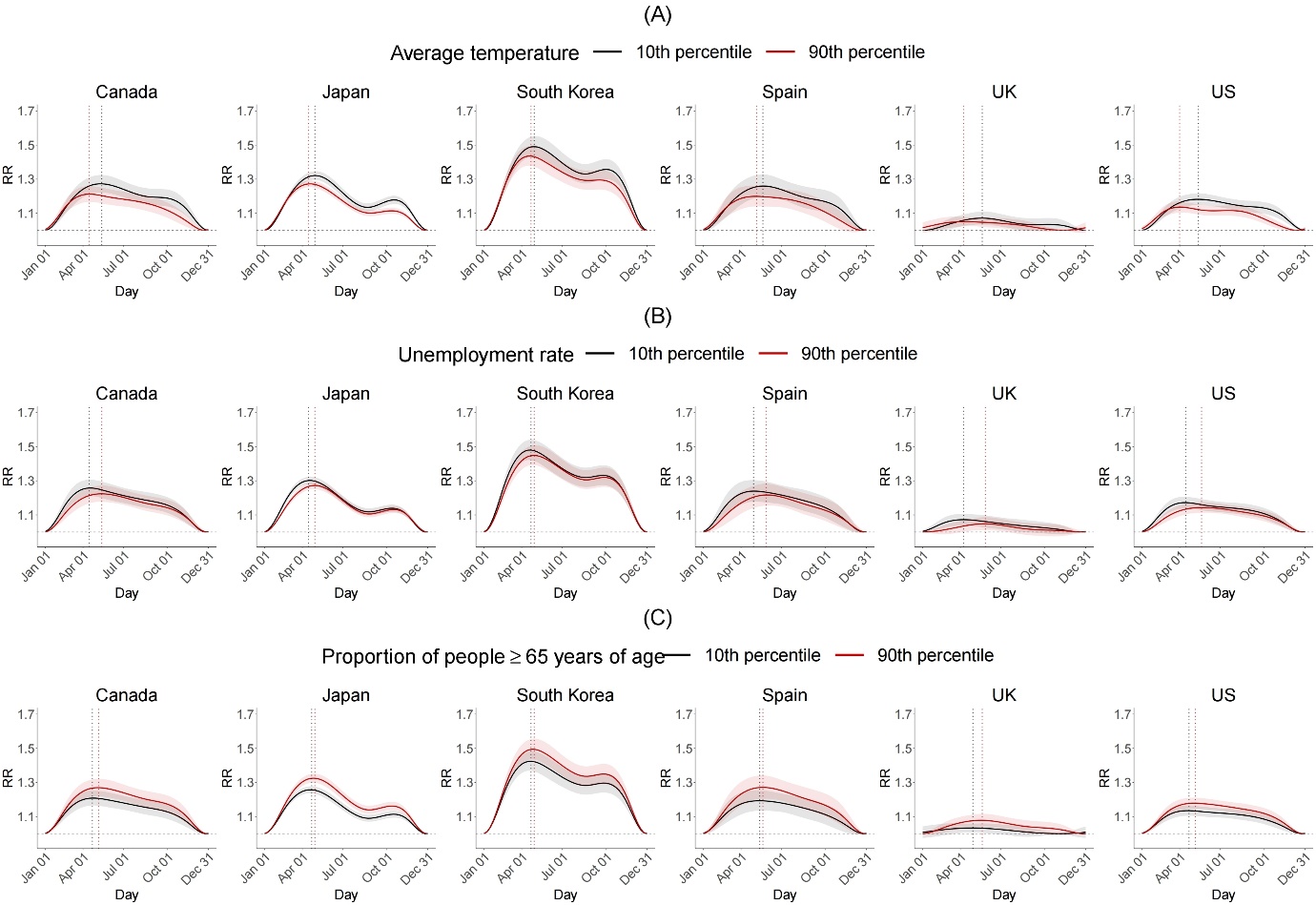


**eFigure 5.**

**
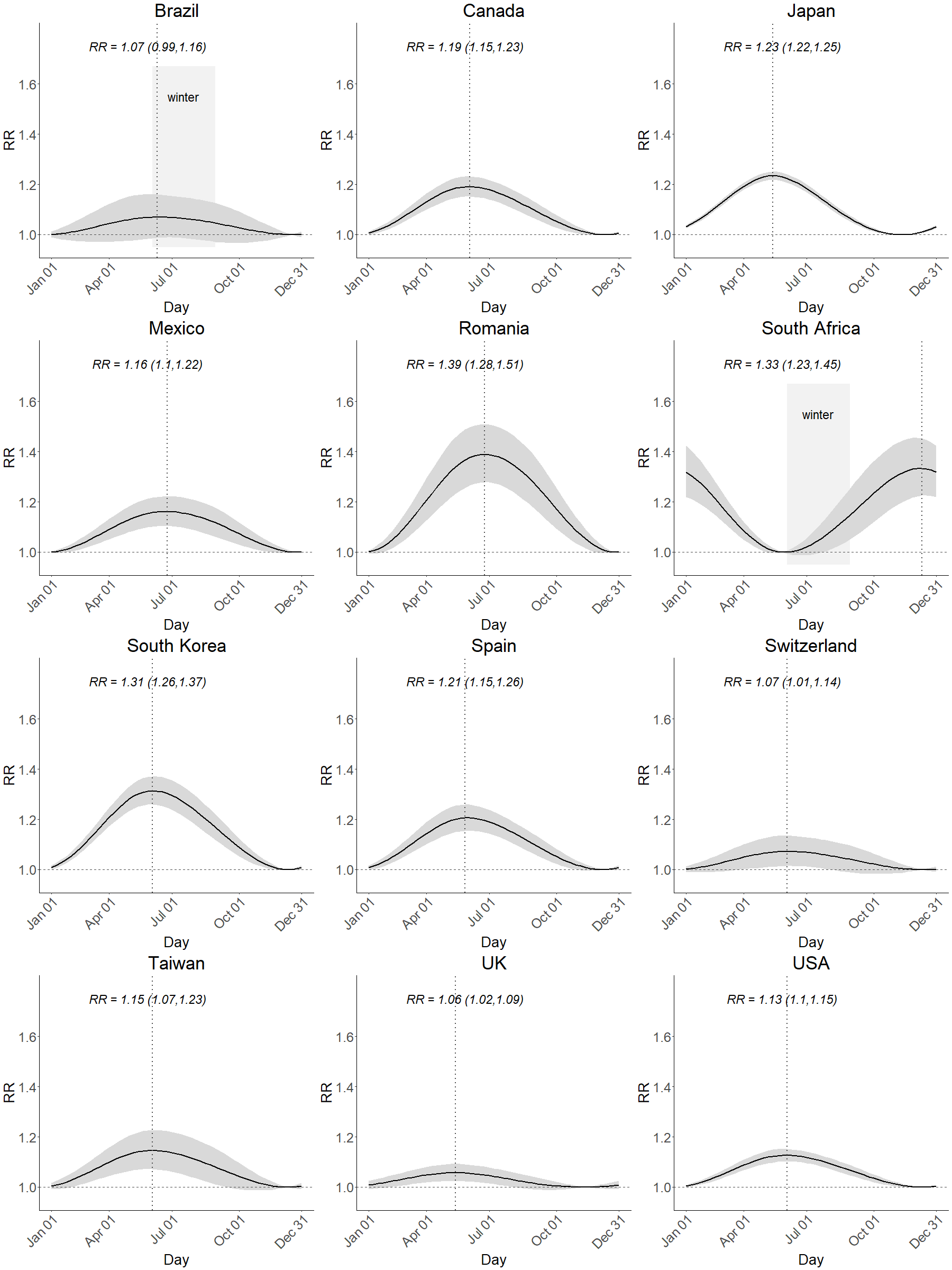
**

**eFigure 6.**


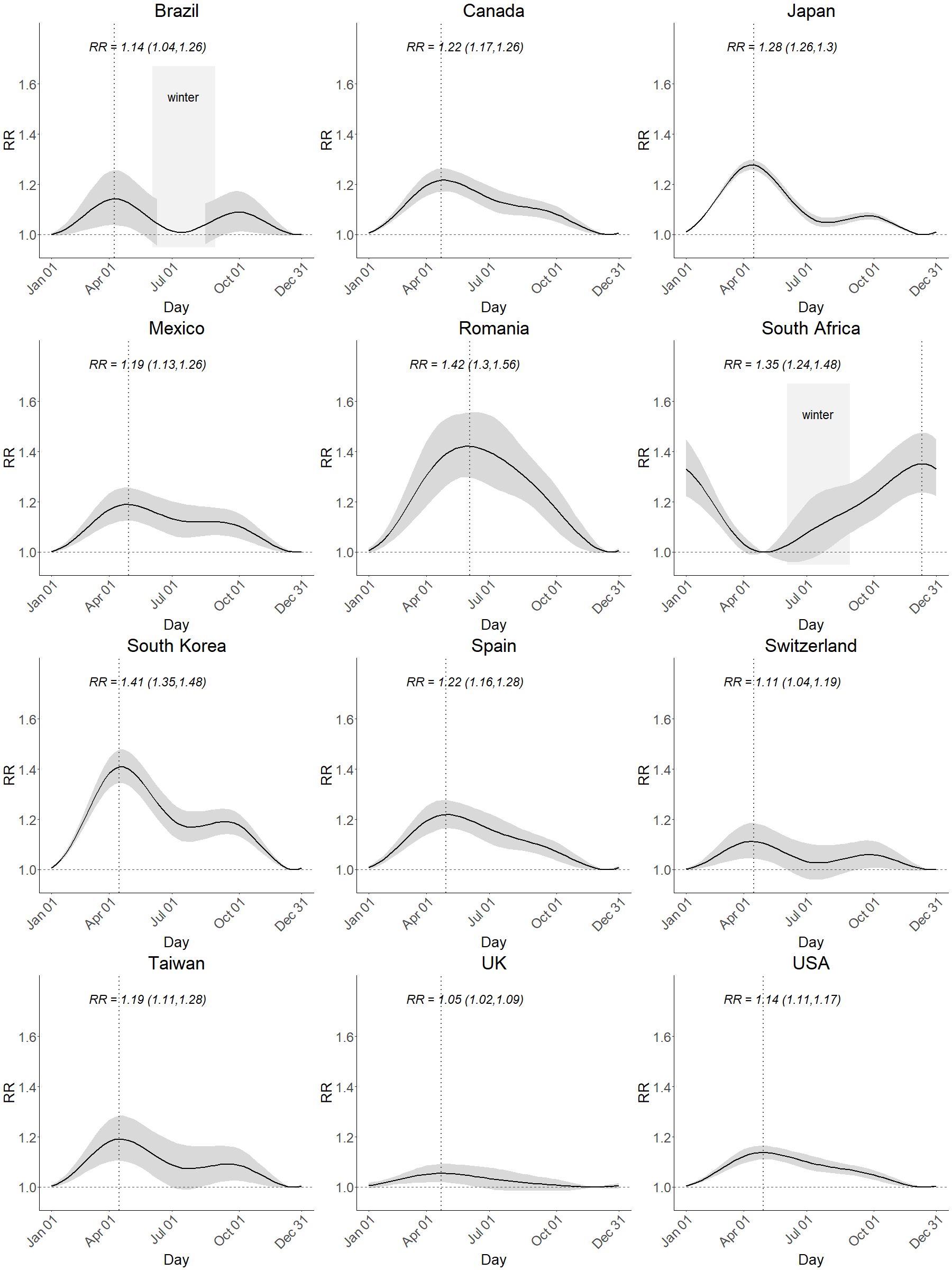


**eFigure 7.**

**
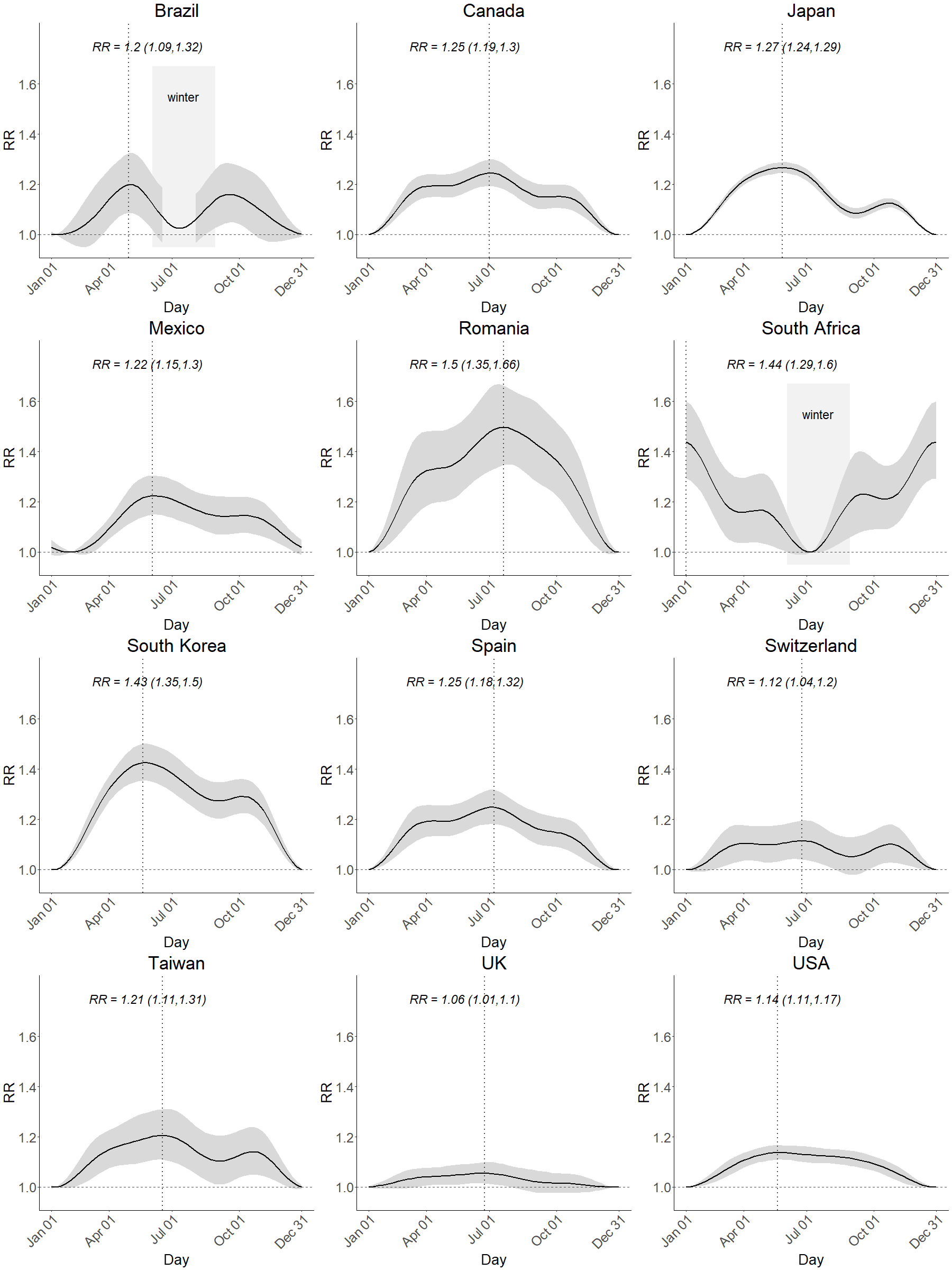
**

**eFigure 8.**


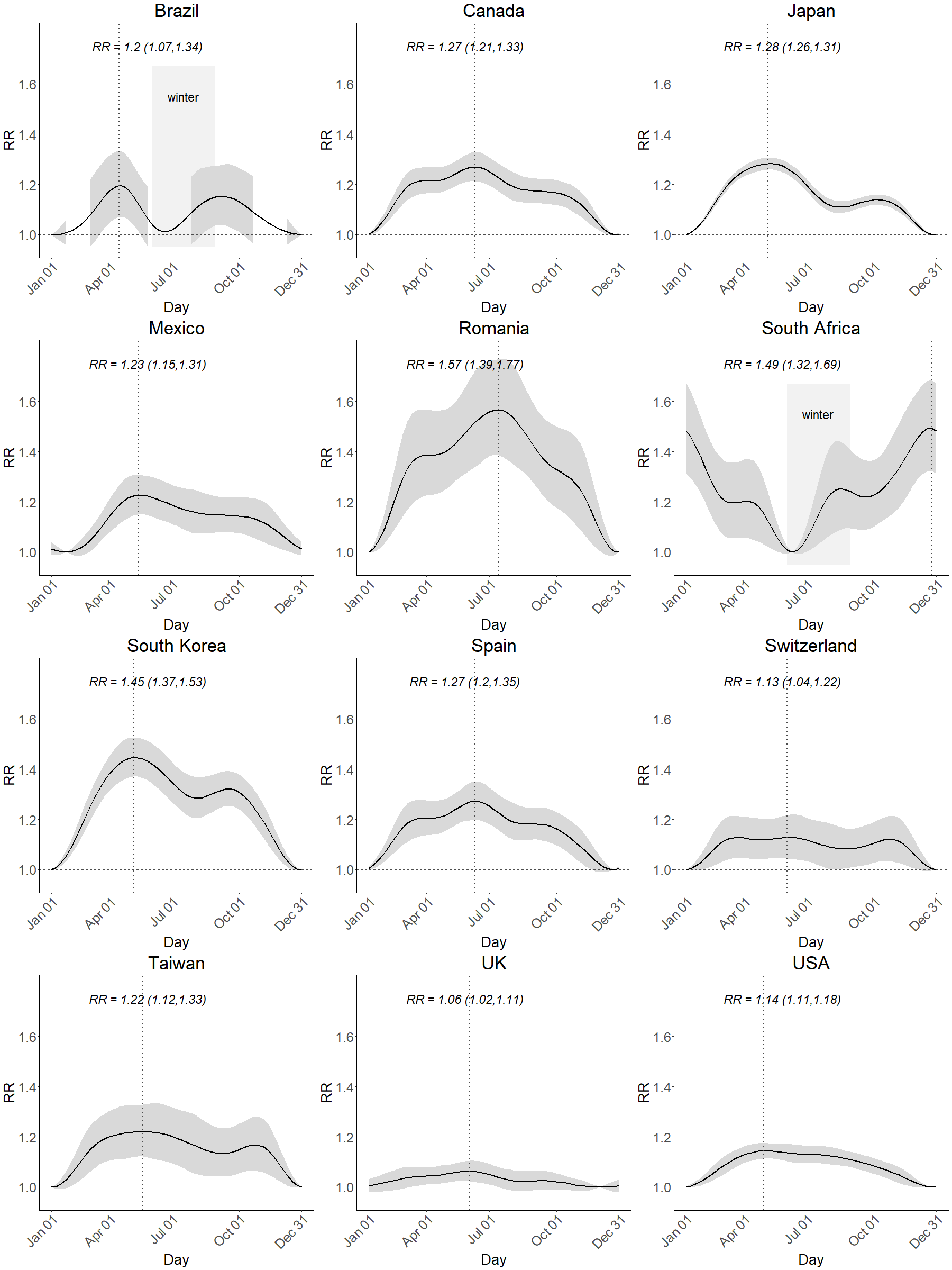


**eFigure 9.**


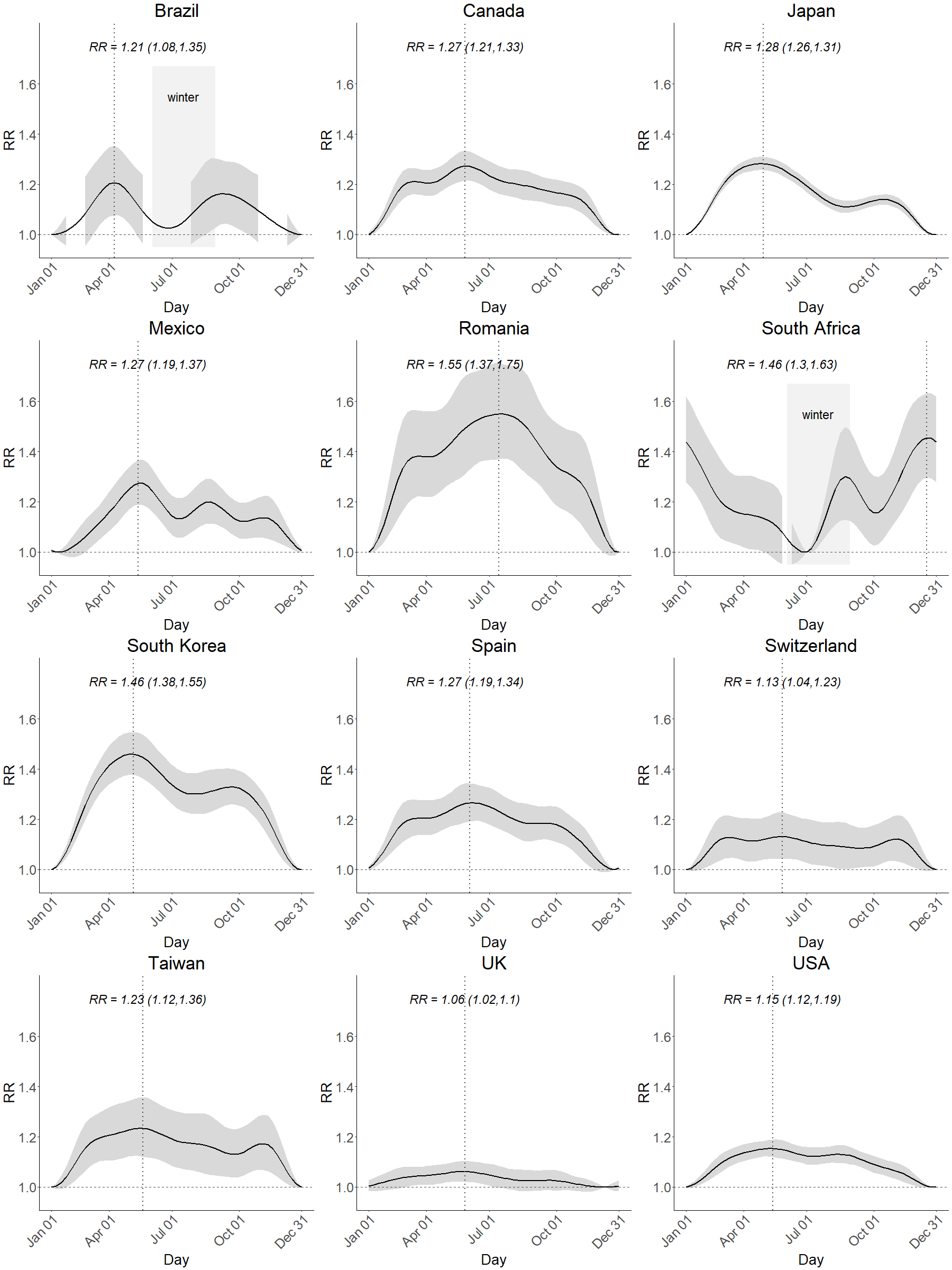

Supplement: Supplementary file 1 [file S2045796020000748sup001.docx]
